# Centrosome function is critical during terminal erythroid differentiation

**DOI:** 10.15252/embj.2021108739

**Published:** 2022-06-09

**Authors:** Péter Tátrai, Fanni Gergely

**Affiliations:** ^1^ Cancer Research UK Cambridge Institute Li Ka Shing Centre University of Cambridge Cambridge UK; ^2^ Department of Biochemistry University of Oxford Oxford UK; ^3^ Present address: Solvo Biotechnology Budapest Hungary

**Keywords:** blood, centrosome, enucleation, erythropoiesis, mitotic spindle, Cell Cycle, Development, Haematology

## Abstract

Red blood cells are produced by terminal erythroid differentiation, which involves the dramatic morphological transformation of erythroblasts into enucleated reticulocytes. Microtubules are important for enucleation, but it is not known if the centrosome, a key microtubule‐organizing center, is required as well. Mice lacking the conserved centrosome component, CDK5RAP2, are likely to have defective erythroid differentiation because they develop macrocytic anemia. Here, we show that fetal liver‐derived, CDK5RAP2‐deficient erythroid progenitors generate fewer and larger reticulocytes, hence recapitulating features of macrocytic anemia. In erythroblasts, but not in embryonic fibroblasts, loss of CDK5RAP2 or pharmacological depletion of centrosomes leads to highly aberrant spindle morphologies. Consistent with such cells exiting mitosis without chromosome segregation, tetraploidy is frequent in late‐stage erythroblasts, thereby giving rise to fewer but larger reticulocytes than normal. Our results define a critical role for CDK5RAP2 and centrosomes in spindle formation specifically during blood production. We propose that disruption of centrosome and spindle function could contribute to the emergence of macrocytic anemias, for instance, due to nutritional deficiency or exposure to chemotherapy.

## Graphical abstract

While centrosomal CDK5RAP2 is not essential for gamma‐tubulin recruitment during the mammalian cell cycle, its absence causes erythroid enucleation defects leading to macrocytic anemia.

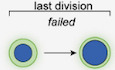

## Introduction

The centrosome is a small non‐membranous subcellular organelle, which comprises two cylindrical centrioles that are embedded in a protein‐rich matrix called the pericentriolar material (PCM). Through nucleating and tethering microtubules (MT), the centrosome acts as an important microtubule organizing center (MTOC) in both proliferating and non‐proliferating cells. Centrosomes undergo a duplication cycle that coincides with DNA replication in the S‐phase and involves the templated assembly of a single procentriole per each old centriole. The master regulator of centriole biogenesis is Polo‐like kinase 4 (PLK4) that acts together with the essential structural proteins SAS‐6 and STIL (Yamamoto & Kitagawa, 2020). During G2, centrosomes mature by increasing the size and nucleating capacity of the PCM, and upon entry into mitosis, the two centrosomes separate to facilitate bipolar spindle assembly. γ‐tubulin‐mediated MT nucleation and anchorage at the centrosome is mainly driven by CEP192 and further enhanced by AURORA‐A and PLK1 kinase cascade in mitosis (Gomez‐Ferreria *et al*, 2007; Lee & Rhee, 2011; Joukov *et al*, 2014; O'Rourke *et al*, 2014). The tight bond between parental centrioles and their procentriole is dissolved in mitosis, thereby allowing daughter centrioles to accumulate PCM in the following cell cycle. CEP215/CDK5RAP2 (cyclin‐dependent kinase 5 regulatory subunit‐associated protein 2) and pericentrin (PCNT) are important for PCM assembly and form the mitotic PCM scaffold (Megraw *et al*, 1999; Sawin *et al*, 2004; Fong *et al*, 2008; Lee & Rhee, 2011; Conduit *et al*, 2014; Woodruff *et al*, 2015; Feng *et al*, 2017). CDK5RAP2 and PCNT are interdependent for their centrosomal localization in mitosis but CDK5RAP2 seems non‐essential for γ‐tubulin recruitment during the mammalian cell cycle (Haren *et al*, 2009; Kim & Rhee, 2014; Gavilan *et al*, 2018). In addition, CDK5RAP2 connects centrioles with the PCM at mitotic spindle poles (Lucas & Raff, 2007; Barr *et al*, 2010; Chavali *et al*, 2016) and promotes centrosome cohesion from G1‐ through S‐ and G2‐phases (Graser *et al*, 2007).

Although the majority of proliferating animal cells contain centrosomes, these organelles have been suggested to play cell‐type‐specific roles such as their contribution to T‐cell‐mediated killing (Stinchcombe *et al*, 2006). Whether centrosomes have additional cell‐type‐specific functions in the hematopoietic lineage, and in particular, during red blood cell development, is not known. The latter is especially of interest because adult mice with mutations in the PCM component *Cdk5rap2* have fewer but bigger red blood cells (RBC), which is defined as macrocytic anemia (Russell, 1979; Lizarraga *et al*, 2010).

**Figure 1:**
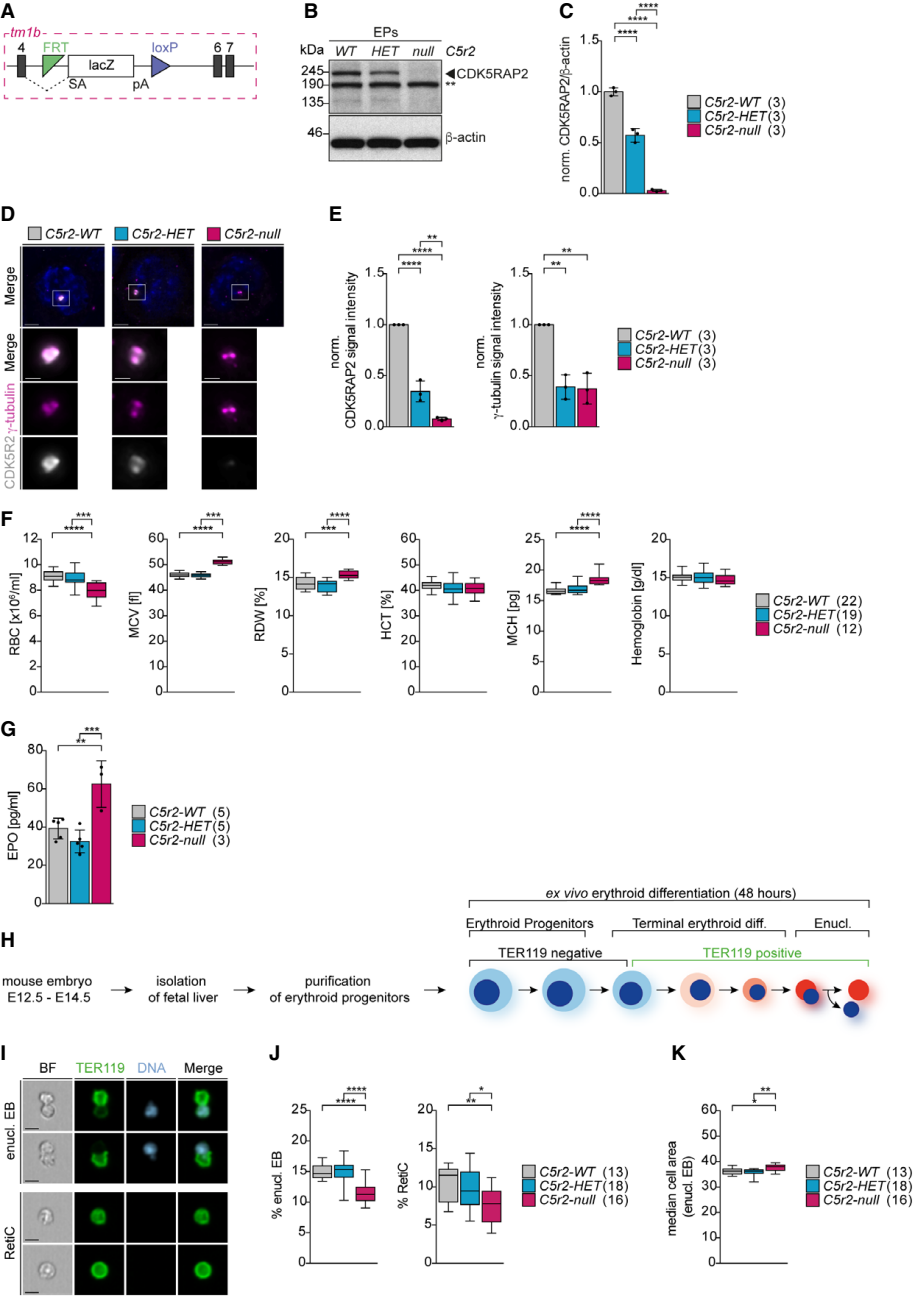
*Ex vivo* differentiation of *Cdk5rap2^null^* erythroblasts recapitulates key features of macrocytic anemia Schematic of Cdk5rap2^null^ (Cdk5rap2^tm1b^) allele generated from the EUCOMM‐knockout first allele by Cre‐mediated deletion of exon 5.Immunoblot showing CDK5RAP2 levels in *Cdk5rap2 wild‐type* (*WT*), *heterozygous* (*HET*), and *null* erythroid progenitors (EPs) isolated from fetal livers. Actin was used as loading control. ** indicates non‐specific band.Quantification of mean protein levels from (B). Numbers in brackets correspond to number of embryos analyzed.Immunofluorescence images of *Cdk5rap2 WT*, *HET*, and *null* erythroid progenitors isolated from fetal livers. Progenitors were stained for CDK5RAP2 (grey), γ‐tubulin (magenta), and DNA (Hoechst, blue). Images are maximum intensity projections of deconvolved z‐stacks. Scale bar, 3 μm. Insets show higher magnification of centrosomes. Scale bar, 1 μm.Quantification of mean centrosomal signal intensities of CDK5RAP2 and γ‐tubulin from (D). Numbers in brackets correspond to number of embryos analyzed with a total number of 470 (*WT*), 406 (*HET*), and 379 (*null*) progenitors.Complete blood count analysis from adult mice with genotypes as indicated. The number of mice analyzed is shown in brackets. RBC = red blood cell. MCV = mean corpuscular volume. RDW = red blood cell distribution width. HCT = hematocrit. MCH = mean corpuscular hemoglobin.Quantification of serum erythropoietin (EPO) levels from adult mice with genotypes as indicated. The number of mice analyzed is shown in brackets.Schematic of the *ex vivo* differentiation culture system.ImageStream images of *ex vivo* cultured enucleating EBs and reticulocytes. Cells were stained for TER119 (erythroid marker, green) and DNA (Hoechst, blue). BF: bright field. Scale bar, 5 μm.Quantification of enucleating EBs and reticulocytes after 48 h (T48) in *ex vivo* culture. Genotypes are as indicated. The numbers in brackets correspond to the number of embryos analyzed.Quantification of enucleating EB size from (I). The numbers in brackets refer to the number of embryos analyzed. Data information: Box plots show 5^th^ and 95^th^ (whiskers) and 25^th^, 50^th^, and 75^th^ percentiles (boxes). Bar graphs display mean ± s.d. Statistical analysis was based on the number of embryos (C, E, I, and J) or number of mice (F and G). Statistical significances were determined by one‐way ANOVA test with Tukey’s (C, E, F, G, J) or Kruskal–Wallis test with Dunn's (K) multiple comparisons. *P ≤ 0.05, **P ≤ 0.01, ***P ≤ 0.001, ****P ≤ 0.0001.

Erythropoiesis describes the process of red blood cell development. In mice, definitive erythropoiesis, the process whereby erythroid precursors differentiate into mature enucleated red blood cells, begins in the fetal liver. In the adult, the main site of steady‐state erythropoiesis is the bone marrow, however, under anemic stress, red blood cells can be produced in the spleen (stress erythropoiesis). Hematopoietic stem cells differentiate into committed erythroid progenitors called BFU‐E (burst‐forming unit) and CFU‐E (colony‐forming unit) after their ability to form morphologically distinct colonies in semi‐solid media (Koury, 2016). During terminal erythroid differentiation, progenitors undergo four to five cell divisions until they eject their nucleus to become reticulocytes (Zhang *et al*, 2003; Sankaran *et al*, 2012). These terminal cell divisions are unusual as they yield daughter cells that are morphologically and functionally different from their mothers. In addition, as they differentiate, erythroblasts (EBs) progressively decrease in cell size, condense their chromatin, and accumulate hemoglobin. Before enucleation, EBs exit the cell cycle, which has been attributed to accumulation of the cyclin‐dependent kinase (CDK) inhibitors P27 (Hsieh *et al*, 2000; Li *et al*, 2006) and P18 (Han *et al*, 2017). P27 and P18 expression is controlled by the erythroid‐specific transcription factor EKLF/KLF1 (Tallack *et al*, 2007; Gnanapragasam *et al*, 2016). The different stages of terminal erythroid differentiation can be distinguished based on expression of surface markers such as the transferrin receptor CD71/CD44 and the erythroid‐specific marker glycophorin A (TER119). TER119 is present throughout terminal differentiation from early erythroblasts to mature red blood cells but absent in erythroid progenitors (Fig 1H) (Chen *et al*, 2009).

Erythroid enucleation can be divided into three stages: nuclear polarization, extrusion, and physical cell separation. In preparation for erythroid enucleation, histone deacetylation promotes chromatin condensation leading to reduced nuclear size and transcriptional inactivation (Popova *et al*, 2009; Ji *et al*, 2010). During polarization, the nucleus migrates toward one side of the cell in an MT‐dependent manner and gets ejected by F‐actin polymerization and actomyosin contraction forces (Konstantinidis *et al*, 2012; Ubukawa *et al*, 2012; Wang *et al*, 2012; Kobayashi *et al*, 2016; Nowak *et al*, 2017). Organelles, including centrosomes, are cleared during and following enucleation by autophagy‐dependent and ‐independent degradation (Watanabe *et al*, 2016; Moras *et al*, 2017). The final step of enucleation, the separation of the nucleus from the nascent reticulocyte, is mediated by vesicle and vacuole trafficking (Keerthivasan *et al*, 2010; Konstantinidis *et al*, 2012). Macrophages, which associate with differentiating EBs in the erythroblastic island, engulf the extruded nuclei (pyrenocytes) and enable release of reticulocytes into the bloodstream where they further mature into erythrocytes.

Several signaling and cytoskeletal components have already been assigned roles during enucleation. Indeed, it has been previously reported that late‐stage EBs (i.e., EBs that undergo one last division with their daughters subsequently enucleating) contain one or two γ‐tubulin‐positive foci, indicative of the presence of MTOCs (Konstantinidis *et al*, 2012; Wang *et al*, 2012; Kobayashi *et al*, 2016). Furthermore, classical electron microscopy studies identified centrioles in enucleating EBs from rabbit bone marrow (Skutelsky & Danon, 1970). However, the functional relevance of these MTOCs/centrosomes in terminal erythroid differentiation and enucleation is not known. Previous studies using small molecule inhibitors of centrosome‐associated mitotic kinases (e.g., PLK1, AURORA‐A) and MT motors (e.g., EG5) in a human erythroid culture system concluded that MTOCs/centrosomes were dispensable for EB enucleation (Ubukawa *et al*, 2012; Kobayashi *et al*, 2016). Inhibiting these pleiotropic regulators may not fully block centrosome function, and thus, contribution by the MTOC/centrosome remains unclear.

Here, we employ an *ex vivo* differentiation system of erythroid progenitors isolated from mouse fetal liver to probe the function of centrosomes, and the PCM in particular, during erythroid differentiation and enucleation. Using a small molecule inhibitor to induce centrosome depletion or by genetic removal of the PCM component *Cdk5rap2*, we show that faithful regulation of spindle assembly in late‐stage EBs is a prerequisite for efficient enucleation. Together, our findings elucidate the underlying cellular mechanism for the macrocytic anemia observed in mice in the absence of CDK5RAP2.

## Results

### *Cdk5rap2^null^* mice exhibit macrocytic anemia

We set out to interrogate the role of centrosomes during erythropoiesis. The starting point for this project was the *Cdk5rap2/Cep215^tm1a^* mouse strain, generated by EUCOMM, which carries a LacZ gene‐trapping cassette (Skarnes *et al*, 2011). By crossing these mice to PGK‐Cre mice, we generated a strain where exon 5 of *Cdk5rap2* is deleted resulting in a LacZ‐tagged null allele (also called *tm1b*) (Fig 1A).

**Figure EV1:**
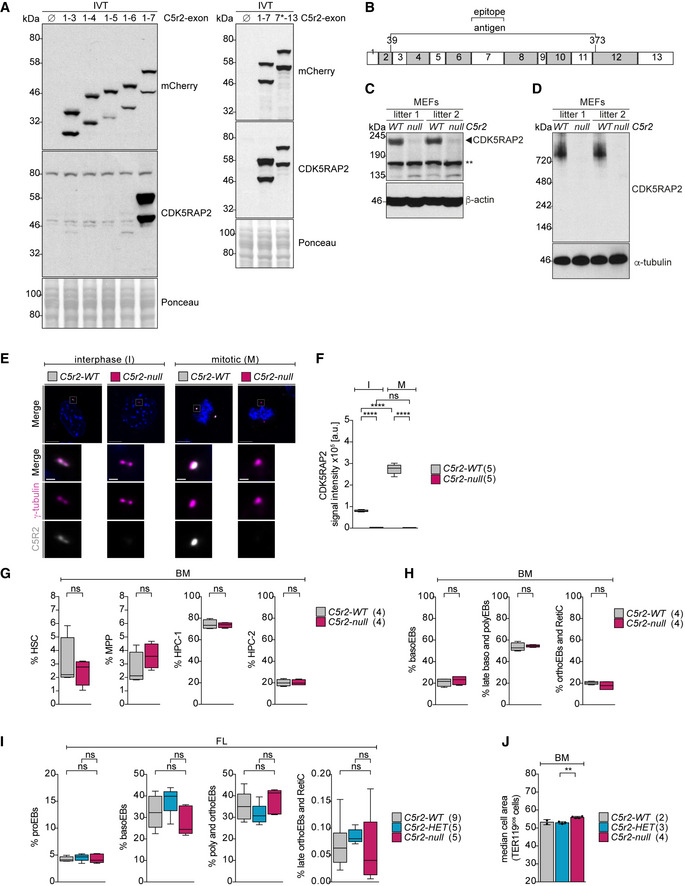
Hematopoietic progenitor pools are largely normal in *Cdk5rap2^null^* mice AImmunoblot showing the detection of mCherry‐tagged IVT. Ponceau‐S staining was used to compare equal loading. 7* marks the alternative start site in exon 7.BSchematic representation showing the antigen and the epitope of CDK5RAP2 N‐terminal antibody as suggested from IVT experiments in (A).CImmunoblot showing CDK5RAP2 levels in *Cdk5rap2 wild‐type* (*WT*) and *null* mouse embryonic fibroblasts (MEFs). Actin was used as loading control. ** indicates unspecific band.DImmunoblot of native gel showing CDK5RAP2 levels in *Cdk5rap2 wild‐type* (*WT*) and *null* mouse embryonic fibroblasts (MEFs). Tubulin was used as loading control.EImmunofluorescence images of interphase (I) or mitotic (M) *Cdk5rap2 WT* and *null* mouse embryonic fibroblasts (MEFs). MEFs were stained for CDK5RAP2 (grey), γ‐tubulin (magenta), and DNA (Hoechst, blue). Images are maximum‐intensity projections of deconvolved z‐stacks. Scale bar, 4 μm. Insets show higher magnification of centrosomes. Scale bar, 500 nm.FQuantification of mean centrosomal signal intensities of CDK5RAP2 from (E). Numbers in brackets correspond to number of MEF lines analyzed with 168 (*WT*) and 154 (*null*) interphase cells and 59 (*WT*) and 56 (*null*) mitotic cells.G, HQuantification of hematopoietic stem and progenitor cells (G) and erythroblast stages (H) in bone marrow (BM) of 10‐ to 13‐week‐old mice. Genotypes are as indicated. The number in brackets refers to the number of mice analyzed. HSC = hematopoietic stem cells. MPP = multipotent hematopoietic progenitors. HPC = hematopoietic progenitor cells.IQuantification of erythroblast stages in E13.5 fetal livers. Genotypes are as indicated. The number in brackets refers to the number of embryos analyzed.JQuantification of cell size of TER119^pos^ cells in bone marrow (BM) of 10‐week‐old mice. Genotypes are as indicated. The number in brackets refers to the number of mice analyzed. Data information: Box plots show 5^th^ and 95^th^ (whiskers) and 25^th^, 50^th^, and 75^th^ percentiles (boxes). Bar graph in J displays mean ± s.d. Statistical analysis was based on the number of MEF lines (F), the number of mice (G, H, and J), or the number of embryos (I). Statistical significance was determined by one‐way ANOVA with Tukey’s multiple comparisons test (F and I), Mann–Whitney test (G and H), or two‐tailed unpaired Student's *t*‐test (J). **P ≤ 0.01.

Using our previously published polyclonal N‐terminal antibody against human CDK5RAP2 (Barr *et al*, 2010), we could not detect a protein product of the expected size in cell lysates of *Cdk5rap2^tm1b^* erythroid progenitors, the cell population representing the majority of hematopoietic progenitors cells in the fetal liver (Zhang *et al*, 2003). Likewise, no signal was visible in the centrosomes of these progenitors (Fig 1B–E). *In vitro* transcription/translation (IVT) from cDNA spanning different mouse exons revealed that the protein sequence encoded by exon 7 of mouse *Cdk5rap2* is the main recognition site of this antibody (Fig EV1A and B). On immunoblots of cell lysates, this antibody recognized an additional band below 190 kDa, which appeared identical across all *Cdk5rap2* genotypes both in erythroid progenitors and mouse embryonic fibroblasts (MEFs) (Figs 1B and EV1C). To assess the specificity of this band, we tested three commercial antibodies against C‐terminal sequences of human CDK5RAP2 but none recognized murine CDK5RAP2. In native gel electrophoresis, our antibody recognized a single band in cell lysates of *wild‐type* MEFs, which was missing from *Cdk5rap2^tm1b^* MEFs (Fig EV1D), suggesting that the band below 190 kDa is unique to denaturing conditions and may be non‐specific. Consistently, in immunofluorescence, our antibody stained interphase and mitotic centrosomes of wild‐type but not CDK5RAP2‐deficient MEFs (Fig EV1E and F). We cannot exclude that N‐terminally truncated protein products lacking the first 220 amino acids (corresponding to exons 1–7) are expressed in the mutants, but from genomic databases we found no evidence for splice variants of *Cdk5rap2* that lack exon 7 or where translation starts downstream of exon 7, and so we refer to this strain as *Cdk5rap2^null^* hereafter.

*Cdk5rap2^null^* mice exhibit mild macrocytic normochromic anemia similarly to their parental Cdk5rap2^tm1a(EUCOMM)Wtsi^ strain (International Mouse Phenotypic Service (IMPC), mousephenotype.org). Anemia is defined as a decrease in total amount of red blood cells (RBC) or hemoglobin levels. *Cdk5rap2^null^* mice have fewer but bigger red blood cells (RBC), characteristic of macrocytic anemia (Fig 1F). Their red blood cell size is also more variable, as indicated by a higher red cell distribution width (RDW) value. Because mice lacking CDK5RAP2 have fewer but larger RBC, their hematocrit (HCT) levels are normal. Likewise, total hemoglobin levels in blood are also unaffected (described as normochromic) because CDK5RAP2‐deficient RBC accumulate greater hemoglobin mass per cell (mean corpuscular hemoglobin, MCH) but are reduced in numbers (Fig 1F). The same phenotype was observed in the Hertwig’s anemia (*an/an*) mouse model (Russell, 1979), which was generated by mutagenesis and subsequently shown to carry an in‐frame deletion of exon 4 in *Cdk5rap2* (Lizarraga *et al*, 2010).

Anemia can trigger a compensatory mechanism through increased release of the cytokine erythropoietin (EPO) into the blood to stimulate stress erythropoiesis in the spleen. Consistent with anemic stress, we found elevated EPO levels in *Cdk5rap2^null^* mice (Fig 1G). Additionally, mild‐to‐moderate hyperplasia and extramedullary (i.e., outside of the bone marrow) hematopoiesis were observed in the spleen of homozygous Cdk5rap2^tm1a(EUCOMM)Wtsi^ mice (IMPC, mousephenotype.org). In *Cdk5rap2^null^* adult bone marrow, hematopoietic stem cell and progenitor populations appeared normal (Fig EV1G) with no evidence for an erythroid differentiation block in either the bone marrow or the fetal liver (Fig EV1H and I). Nonetheless, consistent with macrocytic anemia, TER119‐positive cells in *Cdk5rap2^null^* bone marrow were larger than control (Fig EV1J). We therefore reasoned that the defect responsible for macrocytic anemia in *Cdk5rap2^null^* was likely to arise in late terminal erythroid differentiation, possibly during enucleation.

To investigate the underlying mechanism, we employed an *ex vivo* differentiation system that recapitulates key stages of terminal erythroid differentiation (Zhang *et al*, 2003). Erythroid progenitors were isolated from the fetal liver, the site of fetal definitive erythropoiesis, and differentiated *ex vivo* over 48 h (T48) (Fig 1H). We found that EBs lacking CDK5RAP2 are impaired in enucleation; both enucleating EB and reticulocyte populations were reduced at the end point of the culture (Fig 1I and J). In addition, we observed an increase in the size of the enucleating EBs (Fig 1K). These results are in complete agreement with the anemia observed in adult *Cdk5rap2^null^* mice (Fig 1F). In summary, mice lacking CDK5RAP2 show a mild macrocytic anemia and this phenotype can be recapitulated using an *ex vivo* erythroid differentiation system.

### Centrosomes persist during enucleation

**Figure 2:**
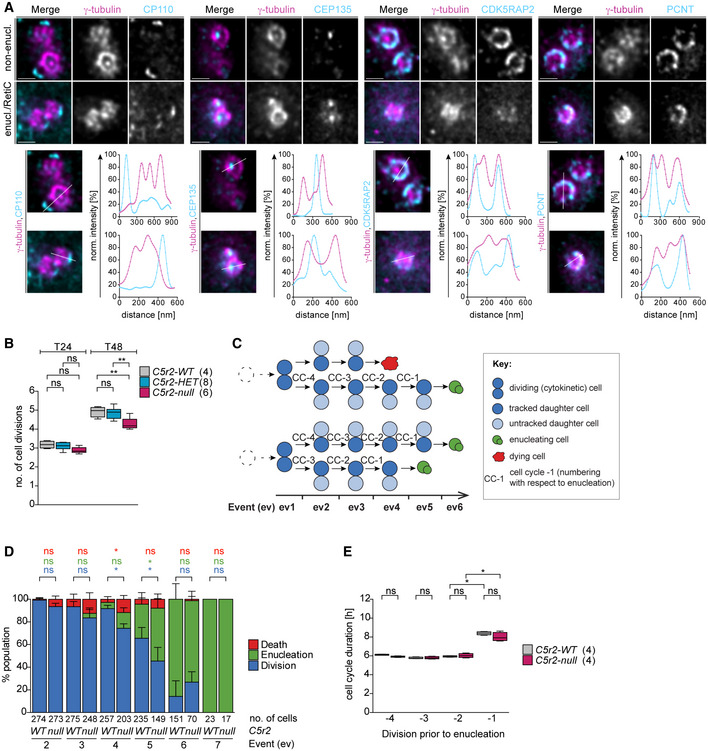
*Cdk5rap2^null^* erythroblasts undergo fewer divisions and enucleate prematurely Deconvolved STED images of centrosomes in *ex vivo* cultured *wild‐type* non‐enucleating EBs (non‐enucl.) or enucleating EBs/reticulocytes (enucl./RetiC). Cells were stained for γ‐tubulin (magenta) and centriolar (CP110, CEP135) or PCM (CDK5RAP2, PCNT) proteins (cyan). Scale bar, 400 nm.Quantification of number of cell divisions after 24 (T24) and 48 (T48) hours. *Ex vivo* cultured erythroid progenitors with indicated genotypes were labeled with PKH26 to measure cell divisions. The numbers in brackets refer to the number of embryos analyzed.Schematic showing how *ex vivo* cultured erythroid progenitors were tracked in bright‐field time‐lapse microscopy experiments. Briefly, the two daughter cells produced by the first cytokinesis and their progeny were followed through terminal erythroid differentiation. Whenever a cell performed cytokinesis, enucleation, or death, this was recorded as an event (ev). Examples depict paths taken by four daughter cells.Quantification of the frequency of cytokinesis, enucleation, and death at each event (ev) in *Cdk5rap2 wild‐type* or *null* erythroid progenitors as shown in (C). Four embryos for each genotype were analyzed.Quantification of cell cycle duration of *Cdk5rap2 wild‐type* or *null* erythroid progenitors. The enucleation event was used as reference point to align previous divisions (see schematic in C). The number in brackets refers to the number of embryos analyzed. Data information: Box plots show 5^th^ and 95^th^ (whiskers) and 25^th^, 50^th^, and 75^th^ percentiles (boxes). Bar graphs display mean ± s.d. All statistical analysis was based on the number of embryos. Statistical significance was determined by one‐way ANOVA with Tukey’s multiple comparisons test (B) or Mann–Whitney *U*‐test (D and E). *P ≤ 0.05, **P ≤ 0.01.

**Figure EV2:**
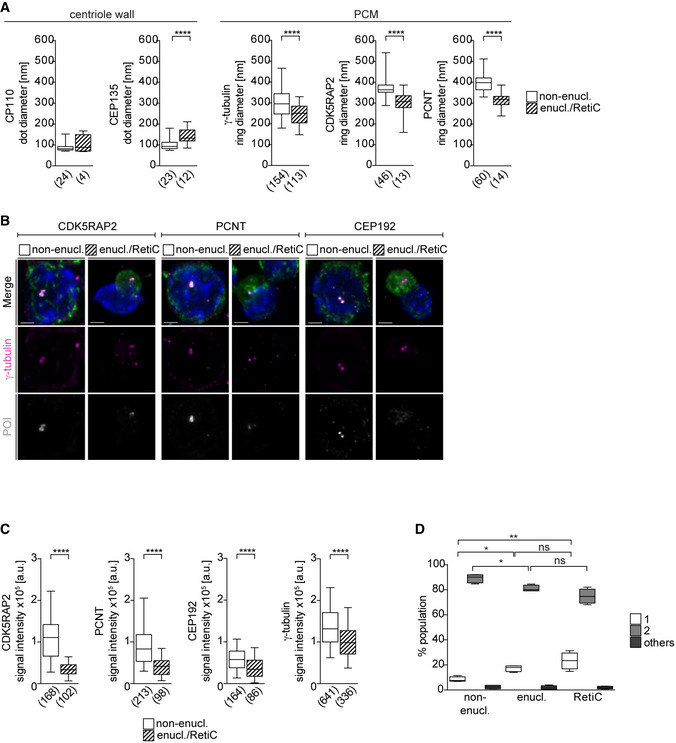
Intact centrosomes are present in enucleating erythroblasts but their PCM is reduced in size Quantification of signal diameters for centriolar (dot) and PCM proteins (ring) from Fig 2A. The numbers in brackets correspond to the number of centrosomes analyzed in one experiment.Immunofluorescence images of *ex vivo* cultured *wild‐type* non‐enucleating EBs (non‐enucl.) or enucleating EBs/reticulocytes (enucl./RetiC). Cells were stained for γ‐tubulin (magenta), protein of interest (POI, CDK5RAP2, PCNT, or CEP192 in grey), TER119 (erythroid marker, green), and DNA (Hoechst, blue). Scale bar, 2 μm.Quantification of mean centrosomal signal intensities of PCM proteins from (B). The numbers in brackets refer to the number of cells analyzed in one experiment.Quantification of centrosome number in *ex vivo* cultured non‐enucleating and enucleating erythroblasts as well as reticulocytes. Four litters with a total number of 2,618 (non‐enucl.), 715 (enucl.), and 877 (RetiC) cells were analyzed. Data information: Box plots show 5^th^ and 95^th^ (whiskers) and 25^th^, 50^th^, and 75^th^ percentiles (boxes). Statistical analysis was based on the number of centrosomes (A), the number of cells (C), or the number of litters (D). Statistical significances were determined by Mann–Whitney test (A and C) or One‐way ANOVA with Tukey's multiple comparisons test (D). *P ≤ 0.05, **P ≤ 0.01, ****P ≤ 0.0001.

CDK5RAP2 is a highly conserved centrosomal protein, and therefore, its function in terminal erythroid differentiation is likely to be linked to centrosomes. However, little is known of what happens to centrosomes during this process. We therefore characterized levels and distribution of key PCM proteins including CDK5RAP2 during terminal erythroid differentiation in *wild‐type* EBs using stimulated emission depletion (STED) super‐resolution microscopy. Several PCM proteins are known to adopt a ring‐shaped pattern corresponding to toroidal protein assembly around the cylindrical wall of an intact centriole (Lawo *et al*, 2012; Sonnen *et al*, 2012). Consistent with continued presence of intact centrioles during erythroid differentiation, such ring‐shaped patterns of PCM proteins were detectable in both non‐enucleating and enucleating EBs/reticulocytes (Fig 2A). However, as EBs progressed through differentiation, the PCM became smaller, whereas centriole diameter remained constant (Fig EV2A). CDK5RAP2 together with other PCM components PCNT and CEP192 co‐localized with γ‐tubulin in interphase centrosomes throughout erythroid differentiation (Fig EV2B). In line with a decrease in PCM size, levels of CDK5RAP2, PCNT, CEP192, and γ‐tubulin were reduced in enucleating EBs and reticulocytes compared to non‐enucleating EBs (Fig EV2A and C). We next determined the number of centrosomes in these populations. Consistent with the presence of two loosely linked parental centrioles, most non‐enucleating and enucleating EBs, and reticulocytes, contained two γ‐tubulin foci. We also noted a small increase in single centriole‐containing cells in the enucleating and reticulocyte population (Fig EV2D). In these cases, the two centrioles may be too close to be resolved but it is also feasible that some cells contain a single centriole or two centrioles with only one incorporating PCM.

In summary, we find that intact centrosomes are maintained during erythroid differentiation. Because cell size of EBs reduces during differentiation, interphase PCM size also decreases, consistent with a previously reported link between centrosome and cell size in *C. elegans* embryos (Decker *et al*, 2011).

### *Cdk5rap2^null^* erythroblasts undergo fewer divisions and enucleate prematurely

**Figure EV3:**
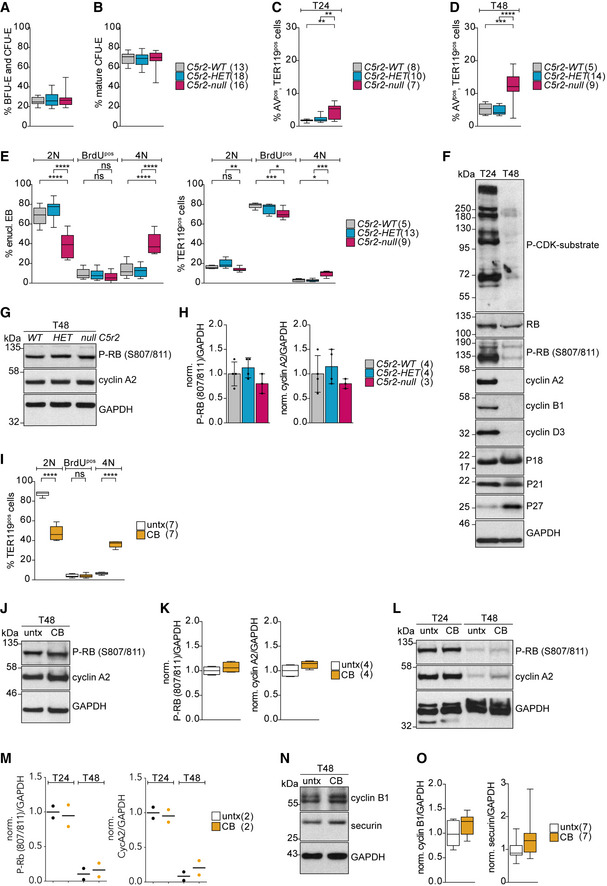
Cell cycle analysis of *Cdk5rap2^null^* erythroid progenitors during *ex vivo* differentiation A, BQuantification of *ex vivo* cultured BFU‐E and CFU‐E (A) as well as mature CFU‐E (B) progenitor populations after 24 h (T24). Genotypes are as indicated. Number of embryos analyzed is shown in brackets.C, DQuantification of apoptotic (AnnexinV^pos^) TER119^pos^ cells at T24 (C) and T48 (D) of *ex vivo* culture. Genotypes are as indicated. Number of embryos analyzed is shown in brackets. T24 = 24 h. T48 = 48 h.EQuantification of cell cycle profiles of *ex vivo* cultured enucleating EBs and TER119^pos^ cells after BrdU pulse for 30 min at 24 h (T24). Genotypes are as indicated. Number of embryos analyzed is shown in brackets.FImmunoblot showing levels of cell cycle markers in *wild‐type* erythroid progenitors after 24 (T24) and 48 (T48) hours of *ex vivo* culture.GImmunoblot showing phospho‐RB (S807/811) and cyclin A2 levels in *Cdk5rap2 wild‐type* (WT), *heterozygous* (HET), and *null* erythroid progenitors at 48 h (T48) of *ex vivo* culture. GAPDH was used as loading control. ** indicates unspecific band.HQuantification of mean protein levels from (G). Number of embryos analyzed is shown in brackets.IQuantification of cell cycle profiles of *ex vivo* cultured TER119^pos^ cells upon CB treatment from Fig 4E. Number of litters analyzed is shown in brackets.JImmunoblot showing phospho‐RB (S807/811) and cyclin A2 levels in CB‐treated erythroid progenitors at 48 h (T48) of *ex vivo* culture. GAPDH was used as loading control.KQuantification of mean protein levels from (J). Number of litters analyzed is shown in brackets.LImmunoblot showing phospho‐RB (S807/811) and cyclin A2 levels in CB‐treated erythroid progenitors at 24 (T24) and 48 (T48) hours of *ex vivo* culture. GAPDH was used as loading control.MQuantification of mean protein levels from (L). Number of litters analyzed is shown in brackets.NImmunoblot sowing cyclin B1 and securin levels in CB‐treated erythroid progenitors at 48 h (T48) of *ex vivo* culture. GAPDH was used as loading control.OQuantification of mean protein levels from (N). Number of litters analyzed is shown in brackets. Data information: Box plots show 5^th^ and 95^th^ (whiskers) and 25^th^, 50^th^, and 75^th^ percentiles (boxes). Bar graphs display mean ± s.d. Statistical analysis was based on the number of embryos (A‐E) or number of litters (F and L). All statistical significances were determined by one‐way ANOVA with Tukey’s multiple comparisons test. *P* ≤ 0.05, **P ≤ 0.01, ***P ≤ 0.001, ****P ≤ 0.0001.

Having established that CDK5RAP2 localization is sustained throughout erythroid differentiation, we next sought to identify its functional contribution to the process. In cells lacking CDK5RAP2, we found no difference in the number of committed erythroid progenitor cells (BFU‐E and CFU‐E) (Fig EV3A and B), suggesting that the abnormalities in red blood cell number and size are more likely to arise from defects in terminal erythroid differentiation or enucleation. We speculated that the appearance of bigger cells in the absence of CDK5RAP2 might result from a reduction in the number of cell divisions during differentiation. To address this, we used the PKH26 membrane dye, which gets diluted with each division, and therefore its intensity inversely correlates with the number of cell divisions. Progenitors are known to divide four to five times before enucleation (Zhang *et al*, 2003; Sankaran *et al*, 2012). Indeed, PKH26 labeling of *wild‐type* erythroid progenitors revealed that cells complete on average five divisions by the 48‐h time point (T48). By contrast, during the same period, erythroid progenitors lacking CDK5RAP2 undergo only four divisions on average. This difference becomes apparent only at T48 because by the 24‐h time point (T24) cells complete on average three divisions independent of their genotype (Fig 2B).

To monitor the behavior of erythroid progenitors, we tracked cells for 48 h by time‐lapse bright‐field microscopy imaging (Movie EV1). We identified cells undergoing division (cytokinesis) and tracked the two daughter cells and their progeny through differentiation. In particular, when a cell underwent division, enucleation, or death, we recorded these as events (ev) (Fig 2C). Although the first event by default was cytokinesis, at subsequent events cells followed different paths. Most WT erythroid progenitors completed five divisions (ev1–5) and enucleated at ev6 (Fig 2D). Indeed, only 5.5 ± 2.6% of wild‐type cells enucleated at ev4 and 30.0 ± 10.2% at ev5. By contrast, 13.9 ± 4.2% of CDK5RAP2‐deficient cells completed three divisions before enucleating at ev4 and 46.7 ± 8.4% completed four divisions before enucleating at ev5 (Fig 2D). These observations are in agreement with PKH‐26 labeling (Fig 2B). Cells lacking CDK5RAP2 also showed an increase in percentage of dead cells, which were confirmed to be apoptotic by Annexin‐V (AV) staining (Fig EV3C and D).

Time‐lapse microscopy enabled us to determine duration of up to four cell division cycles (from cytokinesis to cytokinesis) during terminal erythroid differentiation. From the dataset in Fig 2D, we selected enucleating cells and recorded duration of their cell cycles (CC) preceding enucleation. Intriguingly, we noted that duration of the terminal cell cycle preceding enucleation (CC‐1) was consistently longer in both genotypes (Fig 2C and E). The biological significance of this increase is unclear, but it could reflect reduced cyclin levels or increased activity of CDK inhibitors.

Altogether these data indicate that at least half of CDK5RAP2‐deficient erythroid progenitors complete one fewer cell division than their wild‐type counterparts and hence enucleate prematurely, which would produce fewer reticulocytes. Furthermore, the observed increase in cell death could also contribute to cell loss.

### *Cdk5rap2^null^* erythroblasts enucleate with a 4N DNA content

**Figure 3:**
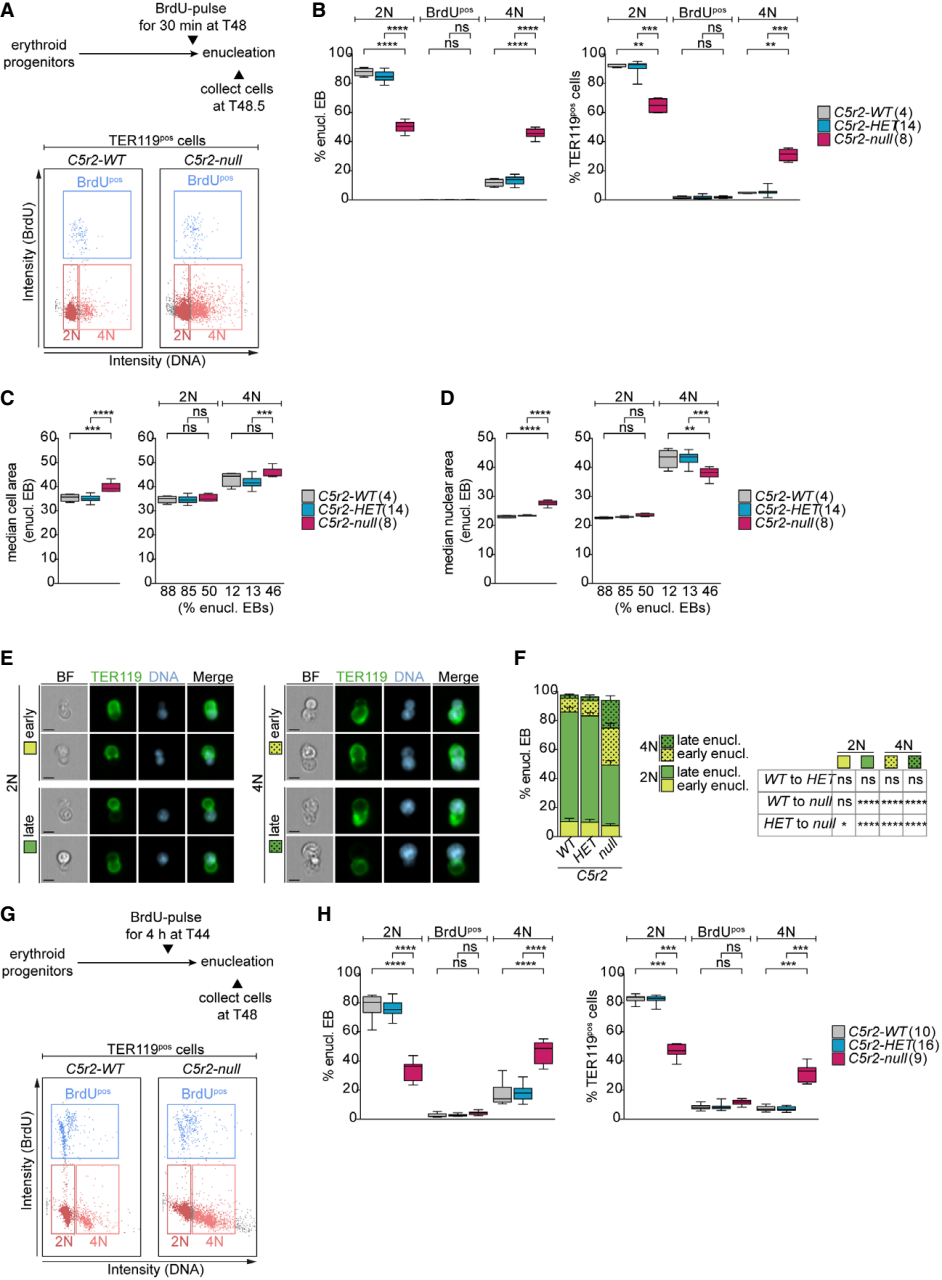
*Cdk5rap2^null^* erythroblasts enucleate with 4N DNA content ASchematic showing the experimental design for BrdU labeling of erythroid progenitors. Exemplary gating profiles of TER119^pos^ cells are shown. The same gating strategy was applied to all samples.BQuantification of cell cycle profiles of enucleating EBs and TER119^pos^ cells from (A). Number of embryos analyzed is shown in brackets.C, DQuantification of cell (C) and nuclear (D) size in enucleating EBs according to DNA content. Percentage of enucleating EBs in each category from (B) is shown below the X‐axis. Number of embryos analyzed is shown in brackets.EImageStream images showing EBs enucleating with 2N or 4N DNA content at different stages from (B). EBs were stained for DNA (Hoechst, blue) and TER119 (erythroid marker, green). Scale bar, 5 μm. BF: bright field.FQuantification of enucleation stages according to DNA content from (B). A total of 4 (*WT*), 14 (*HET*), and 8 (*null*) embryos were analyzed.GSchematic showing the experimental design for BrdU labeling of erythroid progenitors. Exemplary gating profiles of TER119^pos^ cells are shown. The same gating strategy was applied to all samples.HQuantification of cell cycle profiles of enucleating EBs and TER119^pos^ cells from (G). Number of embryos analyzed is shown in brackets. Data information: Box plots show 5^th^ and 95^th^ (whiskers) and 25^th^, 50^th^, and 75^th^ percentiles (boxes). Bar graphs display mean ± s.d. All statistical analysis was based on the number of embryos. Statistical significance was determined by multiple comparisons tests: One‐way ANOVA with Tukey's (B and H left, C–D), Kruskal–Wallis with Dunn's (B and H right), and two‐way ANOVA with Tukey’s (F) multiple comparisons. **P ≤ 0.01, ***P ≤ 0.001, ****P ≤ 0.0001.

EBs are known to exit the cell cycle before enucleation (Hsieh *et al*, 2000). To investigate if this is also the case for *Cdk5rap2^null^* EBs that enucleate prematurely, we performed a BrdU‐labeling experiment. EBs were pulsed with BrdU for 30 min at the end of the 48‐h time (T48) course (Fig 3A). As expected, and in agreement with previous reports (Kinross *et al*, 2006), the BrdU^pos^ population was low at this time point in all genotypes. However, while the vast majority of *Cdk5rap2^WT^* and *Cdk5rap2^HET^* EBs enucleated with a 2N DNA content, we found that a remarkable 45.6 ± 3.2% of *Cdk5rap2^null^* EBs enucleated with a 4N BrdU^neg^ DNA content (enucleating 4N‐EBs) (Fig 3B). This increase in the proportion of 4N cells is not only observed in enucleating EBs but also in the TER119^pos^ population (Fig 3B). In *Cdk5rap2* knockouts, 4N cells were infrequent at 24 h (T24) of culture with the majority of TER119^pos^ cells being in S‐phase, indicating that the 4N population arises later during differentiation (Fig EV3E). Importantly, tetraploidy during enucleation can explain why *Cdk5rap2^null^* EBs are larger than normal. Indeed, increased cell size was also evident in the rare cases of *wild‐type* enucleating 4N‐EBs (Fig 3C). Nuclear size was larger in enucleating 4N‐EBs regardless of their genotype (Fig 3D), suggesting that nuclear condensation is not affected by CDK5RAP2 deficiency. Based on these results, we propose that the high proportion of tetraploid EBs is responsible for the increase and variation in cell size of RBCs observed in *Cdk5rap2^null^* mice (Fig 1F).

To evaluate if increased DNA content affects enucleation dynamics, we determined enucleation stage of EBs based on their nuclear shape (Fig 3E). The nucleus adopts an elongated, dumbbell‐like shape at early stages of enucleation and a spherical shape later when the nucleus is completely extruded (Wang *et al*, 2012; Nowak *et al*, 2017). In the absence of CDK5RAP2, the proportion of EBs in early stages of enucleation was significantly higher than in *wild‐type* EBs (Fig 3F). By correlating enucleation stage with DNA content, we found that it is the 4N population that appears most affected with 25.6 ± 3.3% of *Cdk5rap2^null^* 4N‐EBs being in early stages of enucleation in contrast to 7.5 ± 1.5% of diploid EBs (Fig 3F). These data suggest that 4N‐EBs might take longer to complete enucleation.

Finally, because a 4N DNA content is indicative of cells in G2/M‐phase, we wanted to establish whether tetraploid *Cdk5rap2^null^* EBs enucleate from G2/M rather than from G1 following cell cycle exit like *wild‐type* EBs. To address if enucleating 4N‐EBs had recently passed through S‐phase, EBs were pulsed with BrdU for the final 4 h of the 48‐h time course (Fig 3G). Due to the 6‐ to 8‐h cell cycle duration, this 4‐h pulse was expected to label at least 50% of cycling EBs; however, the percentage of BrdU^pos^ enucleating EBs was very low in all genotypes (Fig 3H). In fact, there was hardly any difference between BrdU^pos^ populations of enucleating *Cdk5rap2^null^* EBs pulsed with BrdU for 4 h (Fig 3H) or for 30 min (Fig 3B). The same results were observed in the total TER119^pos^ population (Fig 3H). Therefore, *Cdk5rap2^null^* 4N‐EBs do not perform DNA replication in the 4 h preceding enucleation.

To further test if tetraploidy in *Cdk5rap2^null^* EBs arises from a G2/M arrest, we analyzed expression of several cell cycle markers. Immunoblots of *wild‐type* EBs collected at T24 and T48 confirmed expected expression patterns of phosphorylated retinoblastoma protein (phospho‐RB), cyclins, and CDK inhibitors. In line with the majority of EBs exiting the cell cycle by T48, CDK activity as indicated by phospho‐RB (S807/811) and P‐CDK substrate levels are decreased. Cyclin levels were much reduced while levels of CDK inhibitors P27 and P18 were elevated (Fig EV3F). Phosphorylation of RB at S807/811 indicates a hyper‐phosphorylated status (Chung *et al*, 2019) that is sustained from late G1‐phase until anaphase when RB gets dephosphorylated by PP1 (Ludlow *et al*, 1993). Interestingly, signal intensities of phospho‐RB and cyclin A2 at T48 remained comparable between *wild‐type* EBs and those lacking CDK5RAP2 (Fig EV3G and H).

Taken together, these data indicate that similarly to *wild‐type*, CDK5RAP2‐deficient EBs exit the cell cycle prior to enucleation.

### Centrosome loss during erythroid differentiation causes tetraploidy and impairs enucleation

**Figure 4:**
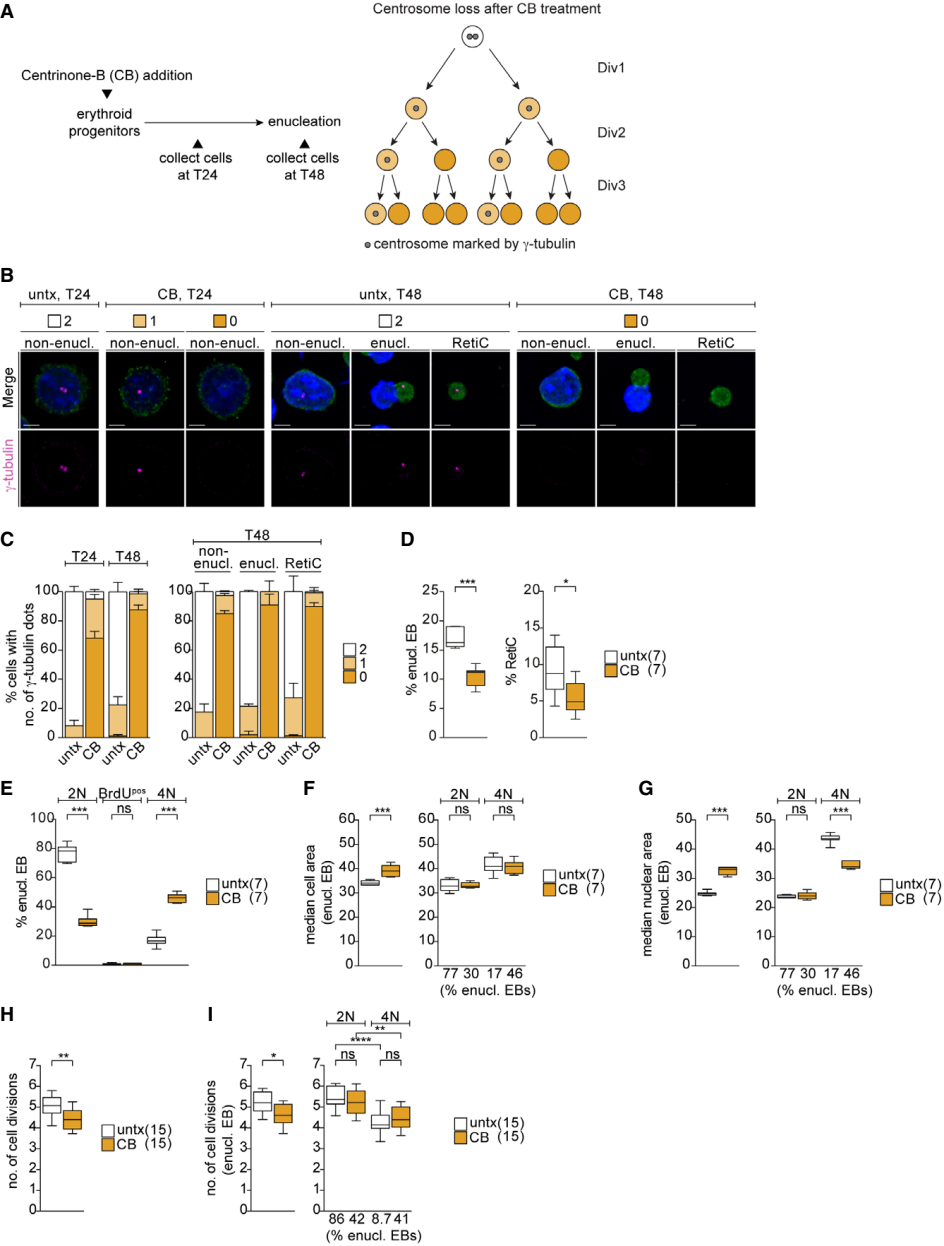
Centrosome depletion closely phenocopies impact of CDK5RAP2 loss on late‐stage erythroblasts ASchematic shows experimental outline of centrinone‐B (CB) treatment of *ex vivo* cultured erythroid progenitors. CB treatment results in centrosome loss over multiple divisions as depicted on the right.BImmunofluorescence images of untreated (untx) or CB‐treated erythroid progenitors at stated differentiation stages after 24 (T24) and 48 (T48) hours *in ex vivo* culture. Cells were stained for γ‐tubulin (magenta), TER119 (erythroid marker, green), and DNA (Hoechst, blue). Scale bar, 3 μm.CQuantification of centrosome numbers (marked by γ‐tubulin) upon CB treatment from (B). For the left panel, in total 632 (untx, T24), 637 (CB, T24), 544 (untx, T48), and 467 (CB, T48) cells from two litters were analyzed. T24 = 24 h. T48 = 48 h. For the right panel, in total 799 (untx, non‐enucl), 142 (untx, enucl), 235 (untx, RetiC), 839 (CB, non‐enucl), 127 (CB, enucl), and 131 (CB, RetiC) cells from two litters were analyzed.DQuantification of enucleating EBs and reticulocytes at T48 of *ex vivo* culture following CB treatment. Number of litters analyzed is shown in brackets.EQuantification of cell cycle profile of enucleating EBs at T48 of *ex vivo* culture following CB treatment. EBs were pulsed with BrdU according to Fig 3A. Number of embryos analyzed is shown in brackets. See Fig EV3I for cell cycle profile of TER119^pos^ cells.F, GQuantification of cell (F) and nuclear (G) size at T48 in all enucleating EBs (left panels in F and G) or according to their DNA content (right panels in F and G) following CB treatment of *ex vivo* culture. Percentage of enucleating EBs in each category from (E) is shown below X‐axis. Number of embryos analyzed is shown in brackets.HQuantification of number of cell divisions at T48 of *ex vivo* culture following CB treatment. *Ex vivo* cultured cells were labeled with the membrane dye PKH26 to monitor cell divisions. Number of embryos analyzed is shown in brackets.IQuantification of number of cell divisions at T48 performed as in (H) following CB treatment of *ex vivo* culture. Left panel shows all enucleating EBs, whereas right panel shows enucleating EBs according to their DNA content. Percentage of enucleating EBs in each category is shown below the X‐axis. Number of embryos analyzed is shown in brackets. Data information: Box plots show 5^th^ and 95^th^ (whiskers) and 25^th^, 50^th^, and 75^th^ percentiles (boxes). Bar graphs display mean ± s.d. Statistical analysis was based on the number of litters (C and D) or number of embryos (E–H). Statistical significance was determined by Mann–Whitney test (D–H and I left) or one‐way ANOVA with Tukey’s multiple comparisons test (I right). *P ≤ 0.05, **P ≤ 0.01, ***P ≤ 0.001, ****P ≤ 0.0001.

We next asked whether the CDK5RAP2 loss phenotype reflects the broader role of centrosomes during erythroid differentiation. To this end, we depleted centrosomes during terminal erythroid differentiation using centrinone‐B, an inhibitor of PLK4, the master regulator of centriole biogenesis (Fig 4A). By T48, centrinone‐B treatment achieved an 87.6 ± 3.3% reduction in centrosome‐containing cells, which is in line with previous reports that complete depletion of centrosomes is achieved only after multiple cell divisions (Wong *et al*, 2015). The degree of centrosome loss was comparable among non‐enucleating EBs, enucleating EBs, and reticulocytes (Fig 4B and C). We found that EBs lacking centrosomes were impaired in enucleation resulting in a reduction in both the enucleating EB and reticulocyte populations at T48 (Fig 4D). This phenotype closely mimics our results with CDK5RAP2‐deficient EBs (Fig 1J and K). Likewise, the proportion of centrinone‐B‐treated EBs enucleating with a 4N DNA content (Fig 4E) was similar to that of EBs lacking CDK5RAP2 (Fig 3B). Again, this change in DNA content was also observed in the TER119^pos^ population (Fig EV3I). In agreement with our previous observations, phospho‐Rb and cyclin A2 levels decrease from T24 and T48 but are comparable between untreated and centrosome‐deficient EBs at T48 (Fig EV3J and K). Levels of the mitotic markers cyclin B1 and securin are similar between untreated and centrinone‐B‐treated cells (Fig EV3N and O). Altogether these observations argue against centrosome‐depleted 4N‐EBs enucleating from G2/M.

Furthermore, similar to *Cdk5rap^null^* enucleating EBs (Fig 3C and D), centrinone‐B treated enucleating EBs displayed increased cell and nuclear size consistent with greater DNA content (Fig 4F and G) and completed fewer cell divisions during differentiation (Fig 4H). We also correlated the number of cell divisions with the DNA content and found enucleating 4N‐EBs to have completed fewer cell divisions when compared to their diploid counterparts, regardless of centrinone‐B treatment (Fig 4I). Intriguingly, we noted a reduction in the nuclear area in both *Cdk5rap2^null^* and centrosome‐depleted 4N‐EBs when compared to untreated 4N‐EBs (Figs 3D and 4G). The reason for this phenomenon is unclear, but we speculate that it could highlight differences in chromatin condensation states. In summary, depletion of centrosomes during terminal erythroid differentiation closely phenocopies loss of CDK5RAP2.

### Erythroblasts lacking CDK5RAP2 or centrosomes fail to establish a bipolar spindle and to initiate anaphase

Our data so far suggest that both CDK5RAP2‐ and centrosome‐deficient EBs develop tetraploidy, which does not correspond to an arrest in G2 (Fig EV3F–M). Thus, the most likely explanation for the 4N phenotype is a cell division failure in these EBs, which is consistent with our PKH26‐labeling results because if cells fail to complete cytokinesis, the label does not get diluted (Figs 2B and 4H).

**Figure 5:**
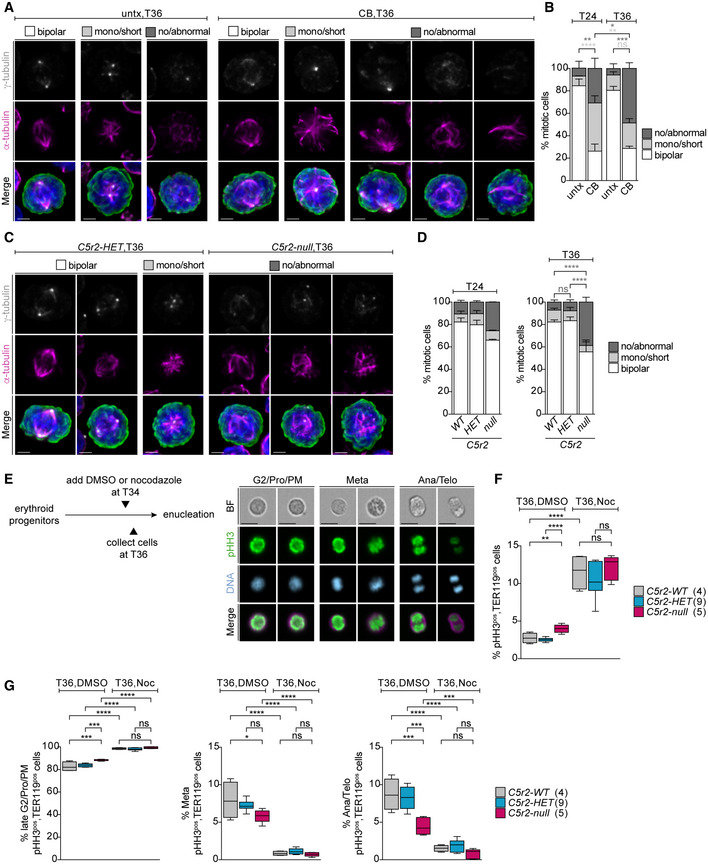
Mitotic spindle assembly is severely impaired in erythroblasts when centrosomes or CDK5RAP2 are absent AImmunofluorescence images of untreated (untx) or centrinone‐B (CB)‐treated cells at 36 h (T36) of *ex vivo* culture. Representative examples for different mitotic spindle morphologies are shown. Cells were stained for α‐tubulin (magenta), γ‐tubulin (grey), phospho‐histone H3 (pHH3, green), and DNA (Hoechst, blue). Images are maximum intensity projections of deconvolved z‐stacks. Scale bar, 2 μm.BQuantification of mitotic spindle morphologies in untreated (untx) or centrinone‐B (CB)‐treated cells at T24 or T36 of *ex vivo* culture. Graph depicts percentage of spindle phenotypes. In total, 315 (untx, T24), 367 (CB, T24), 295 (untx, T36), and 282 (CB, T36) cells were analyzed from three litters.CImmunofluorescence images of *Cdk5rap2^HET^* and *Cdk5rap2^null^* cells at 36 h (T36) of *ex vivo* culture. Representative examples for different mitotic spindle morphologies are shown. Cells were stained for α‐tubulin (magenta), γ‐tubulin (grey), pHH3 (green), and DNA (Hoechst, blue). Images are maximum intensity projections of deconvolved z‐stacks. Scale bar, 2 μm.DQuantification of mitotic spindle morphology at T24 or T36 of *ex vivo* culture. Graph depicts percentage of spindle phenotypes. At T24, three *Cdk5rap2^WT^* (323 cells), three *Cdk5rap2^HET^* (296 cells), and two *Cdk5rap2^null^* (197 cells) embryos were analyzed. At T36, five *Cdk5rap2^WT^* (387 cells), six *Cdk5rap2^HET^* (384 cells), and three *Cdk5rap2^null^* (202 cells) embryos were analyzed.ESchematic (left) shows experimental outline for nocodazole treatment of *ex vivo* cultured erythroid progenitors at indicated time points. Representative ImageStream images of *ex vivo* cultured cells at different mitotic stages (right). Cells were stained for phospho‐Histone H3 (pHH3, green), TER119 (erythroid marker, magenta), and DNA (Hoechst, blue). BF: bright field. Scale bar, 10 μm.F, GQuantification of pHH3^pos^, TER119^pos^, cells and mitotic stages (see text for details) after 36 h (T36) upon DMSO or nocodazole treatment according to (E) using ImageStream. Number of embryos analyzed is shown in brackets. Data information: Box plots show 5^th^ and 95^th^ (whiskers) and 25^th^, 50^th^, and 75^th^ percentiles (boxes). Bar graphs display mean ± s.d. Statistical analysis was based on the number of litters (B) or number of embryos (D, F, and G). Statistical significance was determined by one‐way ANOVA with Tukey's multiple comparisons test (B, D, F, and G). *P ≤ 0.05, **P ≤ 0.01, ***P ≤ 0.001, ****P ≤ 0.0001.

**Figure EV4:**
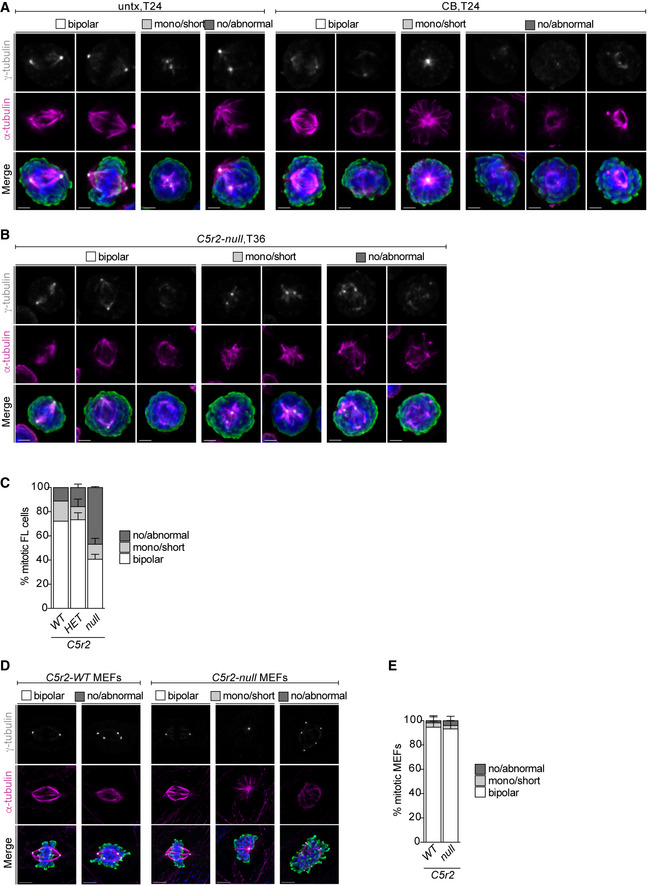
Mitotic spindle morphology in erythroid progenitors lacking centrosomes or CDK5RAP2 Immunofluorescence images of mitotic cells (untx and CB) with different spindle morphologies at 24 h (T24) of *ex vivo* culture. Cells were stained for α‐tubulin (magenta), γ‐tubulin (grey), pHH3 (green), and DNA (Hoechst, blue). Images are maximum‐intensity projections of deconvolved z‐stacks. Scale bar, 2 μm. See Fig 5B for quantification of spindle morphology.Immunofluorescence images of *Cdk5rap2^null^* mitotic cells with different spindle morphologies at 36 h (T36) of *ex vivo* culture. Cells were stained for α‐tubulin (magenta), γ‐tubulin (grey), pHH3 (green), and DNA (Hoechst, blue). Images are maximum‐intensity projections of deconvolved z‐stacks. Scale bar, 2 μm. See Fig 5D for quantification of spindle morphology.Quantification of spindle morphology of mitotic *Cdk5rap2 WT*, *HET*, and *null* cells freshly isolated from E14.5 fetal liver (FL). One *Cdk5rap2^WT^* embryo (72 mitotic cells), three *Cdk5rap2^HET^* embryos (192 mitotic cells), and two *Cdk5rap2^null^* embryos (147 mitotic cells) were analyzed.Immunofluorescence images mitotic mouse embryonic fibroblasts (MEFs) with different spindle morphologies. MEFs were stained for α‐tubulin (magenta), γ‐tubulin (grey), pHH3 (green), and DNA (Hoechst, blue). Images are maximum‐intensity projections of deconvolved z‐stacks. Scale bar, 4 μm.Quantification of spindle morphology in mitotic *Cdk5rap2 WT* and *null* mouse embryonic fibroblasts (MEFs) from (C). Per genotype 5 MEF lines with 58 (WT) or 70 (null) mitotic cells were analyzed. Data information: Bar graphs display mean ± s.d.

Although neither centrosomes nor CDK5RAP2 is considered essential for mitosis in mammalian cells, they can facilitate mitotic spindle assembly. We therefore first investigated spindle morphology in centrosome‐depleted mitotic erythroid progenitors at 24 h (T24) and 36 h (T36). While most untreated cells established a bipolar spindle, the majority of centrinone‐B‐treated cells failed to do so (Fig 5A and B). Abnormal spindle morphologies ranged from multipolar spindles to disorganized MTs that do not emanate from γ‐tubulin‐positive foci. Centrosome‐depleted mitotic cells showed an increase in the percentage of no/abnormal spindles already at T24 (30.8 ± 9.1%) and this phenotype became even more prevalent by T36 (48.5 ± 5.2%) (Figs 5A and B, and EV4A). We next scored spindle morphology in CDK5RAP2‐deficient cells at T24 and T36. Whereas most *Cdk5rap2^WT^* and *Cdk5rap2^HET^* cells established a bipolar spindle by T36, 38.8 ± 4.3 of *Cdk5rap^null^* cells failed to do so (Figs 5C and D, and EV4B). Similar spindle defects were also seen in freshly harvested fetal liver cells (E14.5) (Fig EV4C). This penetrant spindle phenotype is unique to the hematopoietic system, as no such abnormalities were detected in *Cdk5rap2^null^* MEFs (Fig EV4D and E). Previous reports also suggest that CDK5RAP2 is dispensable for bipolar spindle formation and function (Fong *et al*, 2008; Barr *et al*, 2010; Watanabe *et al*, 2020).

To address if spindle assembly failure precluded chromosome segregation and progression through anaphase, we tried to perform time‐lapse microcopy experiments using the live‐cell compatible dye SiR‐DNA but labeled progenitors failed to differentiate and exhibited increased cell death. Instead, we decided to score frequency of anaphase cells in fixed populations, arguing that a failure to initiate chromosome segregation should result in a reduction in anaphase cells. Using ImageStream analysis of phospho‐histone H3‐positive (pHH3^pos^) cells, we determined number of cells at different mitotic phases at T36 (Fig 5E). Consistent with aberrant spindle formation, we noted an increase in the percentage of pHH3^pos^, TER119^pos^ EBs in the absence of CDK5RAP2 (Fig 5F). Based on DNA morphology, mitotic cells were grouped into three categories: late G2/prophase/prometaphase, metaphase, and anaphase/telophase (Fig 5E). While mitotic EBs lacking CDK5RAP2 displayed a small but significant increase in the late G2/prophase/prometaphase population (83.7 ± 3.4% for *Cdk5rap2^WT^* and 88.4 ± 0.7% for *Cdk5rap2^null^*), percentage of EBs in anaphase/telophase was reduced by more than half (8.0 ± 1.2% for *Cdk5rap2^WT^* and 4.5 ± 1.1% for *Cdk5rap2^null^*) (Fig 5G). Thus, CDK5RAP2‐deficient EBs display a small mitotic delay and a reduction in anaphase cells, indicative of a defect in initiating anaphase. Because inactivation of the spindle assembly checkpoint (SAC) normally serves as the trigger for anaphase onset, we tested if SAC signaling was intact in EBs lacking CDK5RAP2 by treating cells for 2 h at the 34‐h time point with nocodazole (Fig 5E). Nocodazole increased the percentage of pHH3^pos^, TER119^pos^ cells fourfold in all genotypes, consistent with unperturbed SAC signaling in *Cdk5rap2^null^* EBs (Fig 5F). As expected, nocodazole‐arrested cells were predominantly in the G2/prophase/prometaphase category based on DNA morphology (Fig 5G).

Our findings suggest that abnormal spindles in EBs lacking CDK5RAP2 or centrosomes trigger the SAC and cells exit mitosis after a short mitotic delay. We speculate that this occurs via mitotic slippage where a premature drop in cyclin B1 levels brings CDK1 activity below the mitotic threshold, hence forcing cells to initiate anaphase without chromosome segregation (Brito & Rieder, 2006).

### Mitotic erythroblasts fail both to expand their centrosomal PCM and recruit PCM components to acentriolar foci

We next asked why mitotic spindle formation fails so frequently in CDK5RAP2‐deficient late‐stage EBs but not in MEFs of the same genotype (Fig EV4D and E). According to recent publications, combined removal of centrosomes and CDK5RAP2 is necessary for spindle disruption in established human cell lines, and in certain cell lines, even such conditions remain permissive to bipolar spindle formation (Watanabe *et al*, 2020; Chinen *et al*, 2021).

**Figure 6:**
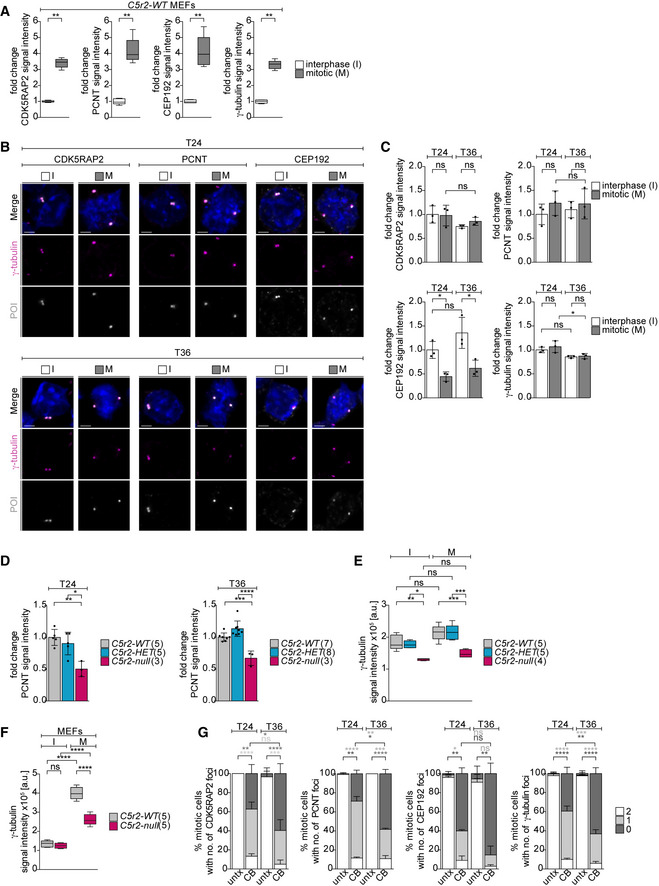
Erythroblasts, unlike MEFs, show no sign of centrosome maturation Quantification of mean centrosomal signal intensities of PCM proteins in wild‐type MEFs. MEF lines (*n* = 5; independently derived) were analyzed with a total of 168 (CDK5RAP2), 158 (CEP192), 164 (PCNT), and 332 (γ‐tubulin) interphase cells and 59 (CDK5RAP2), 68 (CEP192), 61 (PCNT), and 120 (γ‐tubulin) mitotic cells. Note that the same CDK5RAP2 data also form part of Fig EV1F.Immunofluorescence images of cells in interphase (I) and mitosis (M) at 24 (T24) and 36 (T36) hours of *ex vivo culture*. Cells were stained for γ‐tubulin (magenta), protein of interest (POI, CDK5RAP2, PCNT, or CEP192 in grey), and DNA (Hoechst, blue). Images are maximum‐intensity projections of deconvolved z‐stacks. Scale bar, 2 μm.Quantification of mean centrosomal signal intensities of PCM proteins from (B). Three litters were analyzed with the following number of cells for I, T24; M, T24; I, T36; and M, T36: 290, 157, 259, and 174 (CDK5RAP2); 291, 190, 203, and 181 (PCNT); 314, 122, 283, and 152 (CEP192); and 895, 465, 720, and 493 (γ‐tubulin).Quantification of mean centrosomal PCNT signal intensities during mitosis at 24 (T24) and 36 (T36) hours of *ex vivo* culture. Genotypes are as indicated. Number of embryos analyzed is shown in brackets. A total of 227 (*WT*), 282 (*HET*), and 158 (*null*) cells were analyzed for T24 and 370 (*WT*), 348 (*HET*), and 196 (*null*) cells were analyzed for T36.Quantification of mean γ‐tubulin signal intensities at interphase or mitotic centrosomes after 36 h (T36) in *ex vivo* culture. Genotypes are as indicated. Number of embryos analyzed is shown in brackets. A total of 326 (*WT*), 243 (*HET*), and 195 (*null*) interphase cells and 292 (*WT*), 281 (*HET*), and 299 (*null*) mitotic cells were analyzed. See Fig EV6B for representative immunofluorescence images.Quantification of mean centrosomal signal intensities of γ‐tubulin from (EV1E). Numbers in brackets correspond to number of MEF lines analyzed with 168 (*WT*) and 154 (*null*) interphase cells and 59 (*WT*) and 56 (*null*) mitotic cells.Quantification of PCM foci numbers in CB‐treated mitotic erythroid progenitors after 24 (T24) and 36 (T36) hours of *ex vivo* culture. Three litters were analyzed with the following total number of cells for untx, T24; CB, T24; untx, T36; and CB, T36: 141, 221, 174, and 150 (CDK5RAP2); 112, 122, 116, and 85 (PCNT); 165, 183, 145, and 106 (CEP192); and 403, 527, 440, and 411 (γ‐tubulin). See Fig EV5B for representative immunofluorescence images. Data information: Bar graphs display mean ± s.d. Statistical analysis was based on the number of MEF lines (A and F), number of litters (C and G), or number of embryos (D and E). Statistical significance was determined by Mann–Whitney test (A) or one‐way ANOVA with Tukey’s multiple comparisons test (C–G). *P ≤ 0.05, **P ≤ 0.01, ***P ≤ 0.001, ****P ≤ 0.0001.

**Figure EV5:**
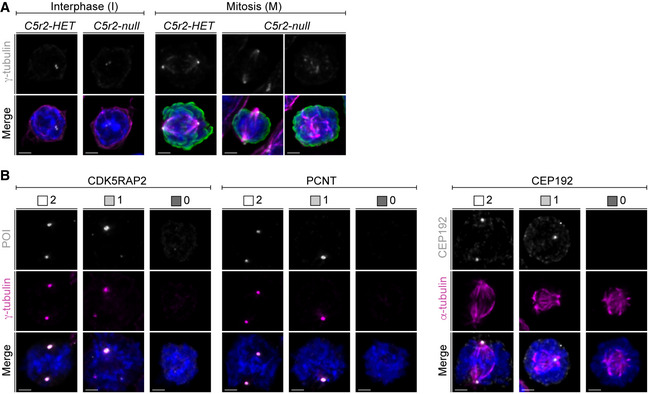
Effect of centrinone treatment on PCM foci formation in erythroid progenitors during *ex vivo* differentiation Representative immunofluorescence images of *ex vivo* cultured *Cdk5rap2 HET* and *null* cells in interphase (I) and mitosis (M). Cells were stained for α‐tubulin (magenta), γ‐tubulin (grey), pHH3 (green), and DNA (Hoechst, blue). Images are maximum‐intensity projections of deconvolved z‐stacks. Scale bar, 2 μm. See Fig 6F for quantification of mean centrosomal signal intensities of γ‐tubulin.Representative immunofluorescence images of *ex vivo* cultured mitotic cells (untx and CB) with different PCM foci numbers. Cells were stained for γ‐tubulin or α‐tubulin (magenta), protein of interest (POI, CDK5RAP2, PCNT, or CEP192 in grey), and DNA (Hoechst, blue). Images are maximum‐intensity projections of deconvolved z‐stacks. Scale bar, 2 μm. See Fig 6G for quantification of PCM foci numbers.

In most model systems, prior to mitotic entry, centrosomes are known to mature by expanding their PCM size and MT nucleation capacity. Indeed, in mitotic *wild‐type* MEFs, mean signal intensities of CDK5RAP2, PCNT, CEP192, and γ‐tubulin all increased three‐ to fourfold (Fig 6A). These results are in stark contrast with EBs where signal intensities of these PCM components remained similar between interphase and mitotic centrosomes at both T24 and T36 (Fig 6B and C). In CDK5RAP2*‐*deficient EBs, levels of centrosomal PCNT decreased (Fig 6D) in line with co‐dependency of CDK5RAP2 and PCNT for centrosomal recruitment (Haren *et al*, 2009; Kim & Rhee, 2014). Absence of CDK5RAP2 also impaired γ‐tubulin recruitment to interphase and mitotic centrosomes of EBs at T36 (Figs 6E and EV5A). While CDK5RAP2‐deficient MEFs exhibited a decrease in γ‐tubulin at mitotic centrosomes, signal levels were still over twofold greater than in interphase, and spindle assembly was unperturbed (Figs 6F and EV4D and E). Thus, CDK5RAP2‐dependent γ‐tubulin recruitment has cell‐type‐specific consequences to spindle assembly. In particular, CDK5RAP2 appears to play a prominent role in γ‐tubulin recruitment in EBs whose mitotic centrosomes harbor little CEP192 potentially due to limited centrosome expansion.

Centrosome‐depleted cells have been shown to establish a bipolar spindle through CEP192‐mediated coalescence of acentrosomal PCM foci, a process also dependent on CDK5RAP2 and PCNT (Meitinger *et al*, 2020; Watanabe *et al*, 2020; Yeow *et al*, 2020; Chinen *et al*, 2021). This prompted us to analyze acentrosomal PCM foci formation in centrinone‐B‐treated EBs. Number of PCM foci positive for PCNT, CDK5RAP2, and γ‐tubulin remained constant in untreated EBs, whereas ~60% of centrinone‐B‐treated cells were lacking these markers by T36 (Fig 6G). CEP192 signal was even more severely impacted because nearly 90% of centrinone‐B‐treated cells contained no foci at T36 (Figs 6G and EV5B). Therefore, in comparison to established human epithelial cell lines, EBs appear particularly poor at forming CEP192‐containing acentrosomal PCM foci, which could explain why their spindle assembly is so sensitive to centrinone‐B treatment (Meitinger *et al*, 2020; Watanabe *et al*, 2020; Yeow *et al*, 2020; Chinen *et al*, 2021).

Altogether our results suggest that cell‐type dependency of centrosome expansion/maturation and PCM assembly could underlie cell‐type‐specific differences in mitotic spindle assembly pathways and their vulnerability to internal and external insults.

### TP53 activity is dispensable for defects in erythroblast differentiation caused by absence of CDK5RAP2 and centrosomes

Several recent reports showed that mitotic delay and/or centrosome loss activate the so‐called mitotic surveillance pathway, which leads to TP53‐dependent cell cycle arrest or apoptosis both *in vitro* and *in vivo* (Fong *et al*, 2016; Lambrus *et al*, 2016; Meitinger *et al*, 2016; Phan *et al*, 2021; Xiao *et al*, 2021). We therefore asked if the mitotic delay seen in *Cdk5rap2^null^* EBs triggered TP53 activation, and if so, whether the phenotypes observed during erythroid differentiation were TP53 dependent.

**Figure 7:**
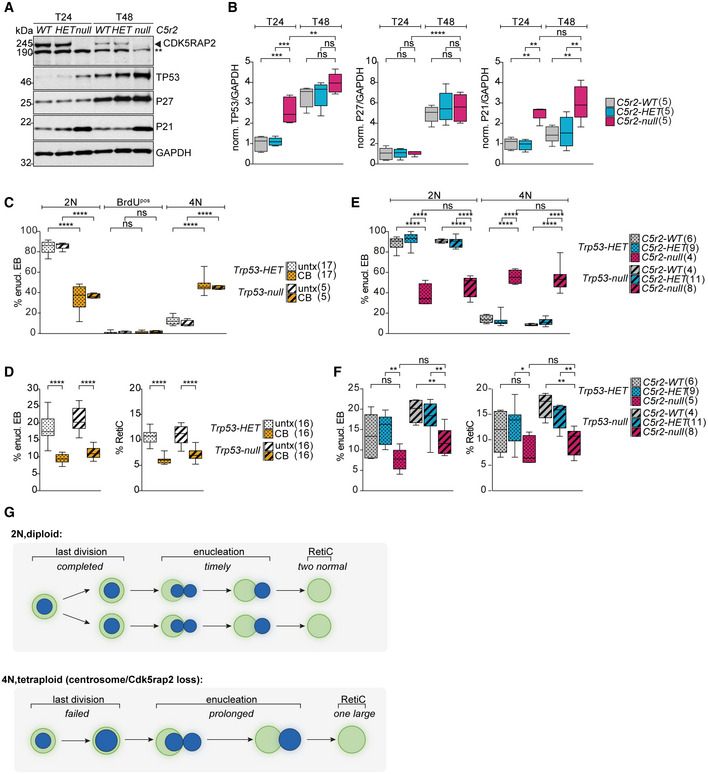
Elevated TP53 activity resulting from centrosome or CDK5RAP2 loss is not responsible for abnormal erythroblast differentiation Immunoblot analysis of TP53, P27, and P21 levels in *ex vivo* cultured Cdk5rap2 *wild‐type* (*WT*), *heterozygous* (*HET*), and *null* EBs after 24 (T24) and 48 (T48) hours. GAPDH was used as loading control. ** Indicates unspecific band.Quantification of mean protein levels from (A). Numbers in brackets represent number of embryos analyzed.Quantification of cell cycle profiles of *ex vivo* cultured *Trp53^HET^* or *Trp53^null^* enucleating EBs with or without CB treatment. EBs were pulsed with BrdU according to Fig 3A. Numbers in brackets represent number of embryos analyzed. See Fig EV6C for cell cycle profiles of TER119^pos^ cells.Quantification of *ex vivo* cultured *Trp53^HET^* or *Trp53^null^* enucleating EBs and reticulocytes after 48 h (T48) upon CB treatment. Numbers in brackets represent number of embryos analyzed.Quantification of cell cycle profiles of *ex vivo* cultured enucleating EBs with the indicated genotypes after 48 h (T48). Number of embryos analyzed is shown in brackets. See Fig EV6F for cell cycle profiles of TER119^pos^ cells.Quantification of *ex vivo* cultured enucleating EBs and reticulocytes with the indicated genotypes after 48 h (T48). Number of embryos analyzed is shown in brackets.Model shows the origin of EBs enucleating with 2N versus 4N DNA content and the consequences on reticulocyte production. Data information: Box plots show 5^th^ and 95^th^ (whiskers) and 25^th^, 50^th^, and 75^th^ percentiles (boxes). All statistical analysis was based on number of embryos. All statistical significances were determined by one‐way ANOVA with Tukey's multiple comparisons test. *P ≤ 0.05, **P ≤ 0.01, ***P ≤ 0.001, ****P ≤ 0.0001.

**Figure EV6:**
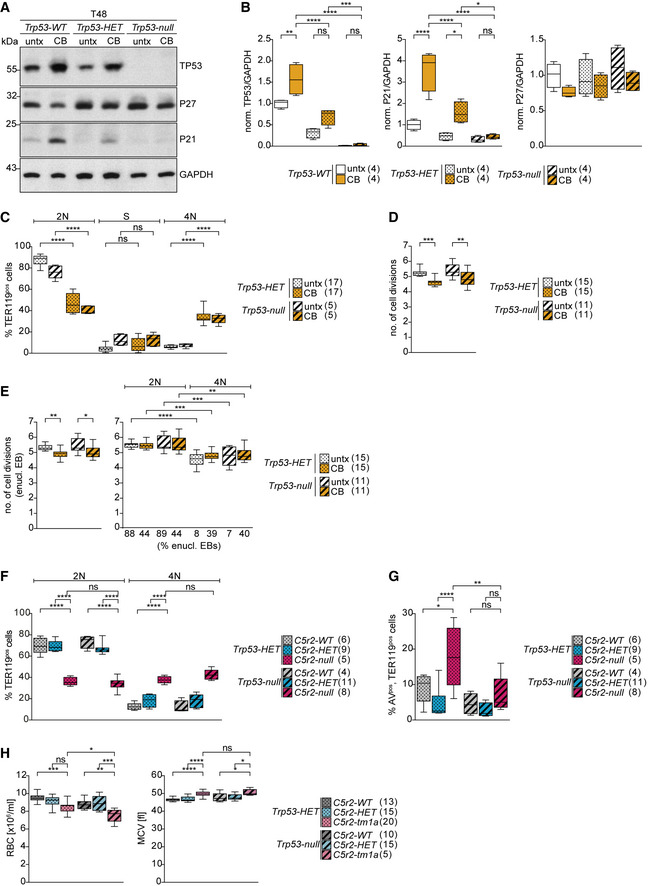
Terminal erythroid differentiation is impaired in *Trp53* and *Cdk5rap2* double mutants both *ex vivo* and *in vivo* Immunoblot showing levels of TP53, P27, and P21 *ex vivo* cultured erythroid progenitors upon CB treatment. GAPDH was used as loading control.Quantification of mean protein levels from (A). Numbers in brackets represent number of embryos analyzed.Quantification of cell cycle profiles of *ex vivo* cultured *Trp53^HET^* or *Trp53^null^* TER119^pos^ cells following CB treatment from Fig 7C. Numbers in brackets represent number of embryos analyzed.Quantification of number of cell divisions at 48 h (T48) of *ex vivo* culture following CB treatment. *Ex vivo* cultured *Trp53^HET^* or *Trp53^null^* EBs were labeled with PKH26 to measure cell divisions. Number of embryos analyzed is shown in brackets.Quantification of number of cell divisions of CB‐treated enucleating EBs with indicated DNA content from (D). Percentage of enucleating EBs in each category is shown below X‐axis. Number of embryos analyzed is shown in brackets.Quantification of cell cycle profiles of *ex vivo* cultured TER119^pos^ cells from Fig 7E. Number of embryos analyzed is shown in brackets.Quantification of *ex vivo* cultured apoptotic (AnnexinV^pos^) TER119^pos^ cells with indicated genotypes after 48 h (T48). Number of embryos analyzed is shown in brackets.Complete blood count analysis from adult mice with genotypes as indicated. The number of mice analyzed is shown in brackets. RBC = red blood cell. MCV = mean corpuscular volume. Data information: Box plots show 5^th^ and 95^th^ (whiskers) and 25^th^, 50^th^, and 75^th^ percentiles (boxes). Statistical analysis was based on the number of embryos (B‐G) or number of mice (H). All statistical significances were determined by one‐way ANOVA with Tukey's multiple comparisons test. *P ≤ 0.05, **P ≤ 0.01, ***P ≤ 0.001, ****P ≤ 0.0001.

Previous studies demonstrated that the CDK inhibitor P27 accumulates during terminal erythroid differentiation, while levels of P21, another CDK inhibitor, do not change (Hsieh *et al*, 2000; Han *et al*, 2017). Indeed, P27 accumulated over time in *wild‐type* EBs (Fig 7A, T24 vs. T48) and its levels were indistinguishable between CDK5RAP2‐deficient and *Cdk5rap2^WT^* or *Cdk5rap2^HET^* EBs at T48. By contrast, levels of TP53 and P21 were significantly elevated *in Cdk5rap2^null^* EBs at T24 (Fig 7A and B). The same effects were seen in centrinone‐B‐treated EBs (Fig EV6A and B), indicating that CDK5RAP2 deficiency and centrosome loss both trigger TP53 and P21 accumulation.

To test if increased TP53 activity drives the phenotypes observed in centrosome‐deficient EBs, *ex vivo* cultures of erythroid progenitors isolated from *Trp53^HET^ and Trp53^null^* fetal livers were treated with centrinone‐B. Remarkably, centrosome‐deficient EBs initiated enucleation with 4N DNA content regardless of their *Trp53* genotype, and 4N cells were also detectable in the TER119^pos^ population (Figs 7C and EV6C). When assaying enucleating EBs, centrinone‐B‐treated cells completed fewer cell divisions independent of their *Trp53* genotype (Fig EV6D). As in Fig 4I, only enucleating 4N‐EBs and not 2N‐EBs showed a decrease in cell division numbers (Fig EV6E). In addition, TP53‐deficient EBs lacking centrosomes were impaired in enucleation with both the enucleating EB and reticulocyte populations reduced (Fig 7D). As expected, *Trp53^null^* EBs did not accumulate TP53 or P21 in response to centrosome loss, whereas P27 levels were unaffected (Fig EV6A and B).

Finally, we asked whether the phenotypes observed in CDK5RAP2^null^ EBs were also independent of TP53. To this end, we generated a *Cdk5rap2; Trp53* double‐knockout strain. The combined absence of TP53 and CDK5RAP2 did not decrease the proportion of enucleating 4N‐EBs when compared to *Cdk5rap2^null^*; *Trp53^HET^* (Figs 7E and EV6F). Consistently, enucleation remained impaired in double‐knockout EBs (Fig 7F). Deletion of *Trp53* in *Cdk5rap2^null^* EBs restored apoptosis (Annexin V positivity) to levels seen in *Cdk5rap2^WT^Trp53^null^* EBs (Figs EV3D and EV6G). Similar to results from the *ex vivo* culture, TP53 loss failed to restore normal red blood cell number or size in *Cdk5rap2^tm1a(EUCOMM)Wtsi^* mice, albeit the number of double null animals obtained was low (Fig EV6H). Taken together, these data indicate that the phenotypes resulting from CDK5RAP2 deficiency and centrosome loss are independent of TP53 and P21 activity.

## Discussion

In this study, we set out to understand the role of the PCM component CDK5RAP2 and the centrosome itself during terminal erythroid differentiation. Using an *ex vivo* differentiation system, we found that CDK5RAP2‐deficient erythroblasts are impaired in enucleation: they have fewer but larger enucleating erythroblasts, which recapitulates the observed macrocytic anemia in adult mice lacking CDK5RAP2. Nearly half of the CDK5RAP2‐deficient erythroblasts undergo one fewer cell division than *wild‐type* erythroblasts and enucleate with a 4N DNA content. Pharmacological depletion of centrosomes phenocopied these *Cdk5rap2* knockout phenotypes. Despite elevated TP53 levels in *Cdk5rap2^null^* and centrinone‐B‐treated EBs, the phenotypes persisted in the absence of TP53, suggesting that the macrocytic anemia in adult mice might also be independent of TP53. This would be in contrast to the well‐established TP53 dependency of centrosome‐related microcephalies (smaller brain size) (Insolera *et al*, 2014; Marjanovic *et al*, 2015; Phan *et al*, 2021). Based on our results, we propose that late‐stage EBs lacking CDK5RAP2 or centrosomes fail to establish a bipolar spindle in time to allow faithful chromosome segregation and consequently enucleate with a 4N DNA content from a pseudo‐G1/G0 state. The absence of one cell division together with a negative effect of tetraploidy on enucleation culminates in fewer, but larger reticulocytes (Fig 7G).

Previous work on the Hertwig’s anemia (*an/an*), mouse model revealed that while the pluripotent and multipotent stem cell populations were unaffected, the relative number of committed erythroid progenitors (BFU‐E and CFU‐E) was reduced in the *an/an* bone marrow compared to normal littermate controls (Barker *et al*, 1982; Barker & Bernstein, 1983). These data were obtained from colony‐forming assays, and could therefore reflect impaired proliferation, differentiation, or a combination of the two. While we did not observe a difference in the overall number of committed progenitors between CDK5RAP2‐deficient and wild‐type cultures, it is possible that abnormal progenitor proliferation and/or differentiation contribute to the overall reduction in red blood cells in CDK5RAP2‐deficient mice. Nonetheless, such defects cannot explain the macrocytosis phenotype. In addition, the absence of 4N‐TER119^pos^ cells at 24 h indicates that tetraploidy arises during later stages of terminal erythroid differentiation and is not a direct consequence of abnormally dividing erythroid progenitors.

A number of individuals carrying mutations in the *Cdk5rap2* gene have been identified to date. Primary microcephaly and growth retardation are the main phenotypes of these patients; however, no hematological analyses have yet been published (Bond *et al*, 2005; Hassan *et al*, 2007; Pagnamenta *et al*, 2012; Issa *et al*, 2013; Lancaster *et al*, 2013; Tan *et al*, 2014; Yigit *et al*, 2015). Therefore, further studies will be necessary to establish if CDK5RAP2 deficiency also impairs blood development in humans. Intriguingly, one report identified spindle abnormalities in patient lymphocytes, consistent with our observation in late‐stage EBs lacking CDK5RAP2 (Issa *et al*, 2013).

Our study raises the important question as to why the final mitosis of differentiating EBs is so vulnerable. We speculate that due to the ultimate loss of the nucleus by enucleation, there is less evolutionary pressure to maintain genome integrity during the final cell cycle of erythroid differentiation. Indeed, previous reports suggest that premature cell cycle exit or a decrease in spindle gene expression due to excess of free heme might contribute to the emergence of macrocytic anemia (Sankaran *et al*, 2012; Ludwig *et al*, 2015; Doty *et al*, 2019). Furthermore, as gene and protein expression decline (An *et al*, 2014; Gautier *et al*, 2016; Karayel *et al*, 2020), imbalance in PCM components could result in suboptimal centrosomal MT nucleation, thus reducing overall robustness of spindle formation in these late‐stage EBs. Indeed, ~ 15% of wild‐type cells displayed abnormal spindle morphology at T36 (Fig 5D). However, spindle aberrancies were already prevalent in E14.5 fetal liver and at T24 of the *ex vivo* culture (Figs 5D and EV4C), when EBs are at earlier stages of differentiation. Because tetraploidy was rare among EBs at T24 (Fig EV3E), it is feasible that such cells can sustain a mitotic arrest long enough to aid recovery of the spindle. By contrast, their more differentiated counterparts could be more prone to mitotic slippage, hence developing tetraploidy.

Although mitotic spindle architecture is highly conserved through evolution, it was recently shown that both density and stability of spindle MTs increase in a TPX2‐dependent process during neurogenesis in mice (Vargas‐Hurtado *et al*, 2019). Spindle MTs can be generated by multiple mechanisms that are thought to collude in building a robust spindle (Petry, 2016). Increased centrosomal MT nucleation by centrosome maturation is considered important for spindle formation. While mitotic MEFs showed robust centrosome maturation, PCM protein levels were comparable between interphase and mitotic centrosomes of wild‐type EBs (Fig 6A–C). Importantly, we assayed PCM expansion purely by measuring centrosomal levels of key PCM components and not the MT‐nucleating activity of centrosomes. Although a reasonable degree of correlation should exist, we cannot exclude that γ‐tubulin complexes are more active in mitotic EBs. It is also feasible that the lack of PCM expansion is a consequence of interphase centrosomes retaining more PCM in EBs. Nevertheless, γ‐tubulin levels were nearly halved in mitotic centrosomes of CDK5RAP2^null^ cells, suggesting that lack of centrosome maturation in EBs could render mitotic centrosomal MT production and thus spindle assembly more dependent on CDK5RAP2. Indeed, in contrast to CDK5RAP2‐deficient EBs, spindle morphology was normal and centrosomes continued to mature in MEFs of the same genotype (Figs 5D, 6E and F, and EV4D and E).

γ‐tubulin recruitment, and thus MT nucleation, are dictated by the total and phosphorylated amount of CEP192 at mitotic centrosomes (Decker *et al*, 2011; Joukov *et al*, 2014; Yang & Feldman, 2015). In turn, centrosomal recruitment of CEP192 is achieved by two redundant pathways involving centrioles or the PCM components CDK5RAP2 and PCNT (Lawo *et al*, 2012; Kim & Rhee, 2014; Watanabe *et al*, 2020; Chinen *et al*, 2021). As recently reported, mitotic spindle assembly in acentriolar cells is slow, and depends on CEP192 and the coalescence of multiple PCM foci by PCNT and CDK5RAP2 (Watanabe *et al*, 2020; Chinen *et al*, 2021). Combining these observations with ours, we propose that bipolar spindle formation in EBs requires both the centriole‐ and the CDK5RAP2‐dependent pathway of CEP192/γ‐tubulin enrichment. Consequently, EBs that are CDK5RAP2 deficient or depleted of centrosomes frequently fail to establish a bipolar spindle in the allocated time frame.

Our data suggest that defective spindle assembly in late‐stage EBs and the resulting tetraploidy can lead to macrocytic anemia. Importantly, multinucleated EBs have been described in myelodysplastic syndrome (MDS) and congenital dyserythropoietic anemias (CDA) (Cantu Rajnoldi *et al*, 2005; Iolascon *et al*, 2013). Impaired centrosome function and mitotic spindle assembly now join cytokinesis failure (Schwarz *et al*, 2009; Liljeholm *et al*, 2013; Ludwig *et al*, 2015; Seu *et al*, 2020) and mitochondrial dysfunction (Gonzalez‐Menendez *et al*, 2021) as a potential cause for these rare blood disorders.

## Materials and Methods

### Transgenic mice

All animal procedures were performed in accordance with the Animal Welfare and Ethical Body of the CRUK Cambridge Institute (CRUK CI, University of Cambridge) and UK Home Office regulations (in accordance with UK law, Animals Scientific Procedures Act 1986). Mice were housed under specific pathogen‐free conditions and cared for in the CRUK CI Biological Resource Unit.

Mice used in this study were of the C57BL/6 background. *Cdk5rap2^tm1a^* mice were kindly provided by David Adams (Wellcome Sanger Institute). *Cdk5rap2^tm1b^* mice were generated by crossing *Cdk5rap2^tm1a^* mice with PGK‐Cre mice, kindly provided by Prof. Doug Winton (CRUK CI). *Trp53^tm1Tyj^/J* to create *Cdk5rap2^tm1b^; Trp53* double‐mutant mice were also kindly provided by Prof. Doug Winton (CRUK CI). Mice were genotyped using the automated genotyping service from Transnetyx Inc.

Fetal livers (FL) were isolated from E12.5‐E13.5 embryos using timed matings of *Cdk5rap2^HET^* and *Cdk5rap2^HET^* mice, *Trp53^HET^* and *Trp53^HET^* mice, or *Trp53^HET^* and *Trp53^null^* mice. For centrinone‐B treatments, additional timed matings of *wild‐type* mice were used. Mouse embryonic fibroblasts were generated from E14.5 embryos using timed matings of *Cdk5rap2^HET^* and *Cdk5rap2^HET^* mice.

For experiments shown in Figs 7E and F, and EV6F and G, IVF experiments using *Cdk5rap2^HET^*, *Trp53^null^* sperms and *Cdk5rap2^HET^*, *Trp53^HET^* oocytes were used and performed by the GenEdit Core at the CRUK CI.

Genotyping of embryos was performed as follows: embryonic tail was washed once in PBS and added to a tube containing 100 μl Yolk‐Sac‐DirectPCR lysis reagent (Viagen Biotech). Additional 100 μl Yolk‐Sac‐DirectPCR lysis reagent including 50 μl/ml recombinant Proteinase K (EO0492, Thermo Fisher Scientific) was added and samples were digested at 55°C for 3–4 h until dissolved. Heat inactivation was performed for 1 h at 85°C and the crude lysate was stored at 4°C. Genotyping PCR was performed using HotStarTaq DNA Polymerase (Qiagen) with the following primers:

- Cdk5rap2_fwd (GCTGTACCCAACTCTCCACC),
- Cdk5rap2_tm1b_rev (CACAACGGGTTCTTCTGTTAGTCC),
- Cdk5rap2_wt_rev (TAAGGGGTCGTCAGGGGTAG),
- Trp53_fwd (ACAGCGTGGTGGTACCTTAT),
- Trp53_wt_rev (TATACTCAGAGCCGGCCT), and
- Trp53_mut_rev (CTATCAGGACATAGCGTTGG).

### Whole blood counts

Bleeds were taken from the tail vein of 2‐month‐old mice. Bleeds were collected in MiniCollect K3EDTA tubes (Greiner) and analyzed on a Mythic 18 Vet hematology analyzer (Woodley).

### Erythropoietin (EPO) levels

Blood was collected by cardiac puncture of 6‐ to 14‐week‐old mice. Blood was collected in Microvette 500 Z‐Gel tubes (20.1344, Sarstedt) and spun for 10,000 × *g* for 5 min. Serum (supernatant) was transferred into a fresh tube and stored at −80°C. Measurement of EPO levels in serum was performed in duplicate by the core biochemical assay laboratory (CBAL, Cambridge) using the MesoScale discovery U‐plex kit.

### RNA extraction, cDNA preparation, and IVT

Total RNA was isolated from fetal liver cells using Qiagen RNeasy Plus Micro kit (74034, Qiagen) according to manufacturer's protocol with following modifications: fetal liver cells were resuspended in 600 μl RLT buffer by vortexing and lysed by passing them through a 23G needle for several times. Total RNA was eluted in 30 μl RNase‐free water. cDNA was prepared using High‐Capacity RNA‐to‐cDNA Kit (4387406, Applied Bioscience) according to the manufacturer's protocol using 1 μg total RNA. PCR on cDNA was performed using Phusion High‐Fidelity DNA polymerase (M0530, NEB) to amplify exon constructs and cloned into pCS2‐T7‐mCherry (kind gift from T.U. Mayer, University of Konstanz, Germany) to generate IVT. Non‐radioactive IVT was performed using the TNT T7‐coupled wheat germ extract system (L4140, Promega), according to the manufacturer’s protocol, except that the reaction time was increased to 3 h. Five microliter IVT was used for immunoblotting as described below.

### *Ex vivo* culture system and drug treatments

Isolation of erythroid progenitors from E12.5–E13.5 fetal liver was performed as described by Zhang *et al* (2003) with the following modifications: fetal livers were mechanically dissociated in ice‐cold PBS/2%FBS/1 mM EDTA by passing through 18G needle and pipetting. Dissociated cells were passed through a 70 µm cell strainer (Greiner) to prepare a single‐cell suspension. Erythroid progenitors were purified by negative selection using EasySep Mouse Hematopoietic Progenitor Cell Isolation kit (StemCell Technologies) with following adjustments to the manufacturer’s protocol: single‐cell suspension was resuspended in 100 µl PBS/2%FBS/1 mM EDTA. Normal rat serum was used at 10 µl/100 µl cells and Easy Sep Mouse Hematopoietic Progenitor Cell Isolation Cocktail was added at 10 µl/100 µl cells. EasySep Streptavidin RapidSpheres were added at 15 µl/100 µl cells. Cells were incubated with Isolation Cocktail and RapidSpheres each for 20 min on ice. Cell suspension was brought up to a total volume of 500 μl with PBS/2%FBS/1 mM EDTA, and DynaMag‐2 magnet (Thermo Fisher Scientific) was used to collect the beads. The unbound fraction was transferred into a new tube by pipetting and the beads were washed once with 500 µl PBS/2%FBS/1 mM EDTA. The wash fraction was added to the tube that contained the corresponding unbound fraction.

Fetal livers from genetically modified embryos were treated separately, whereas purified erythroid progenitors from *wild‐type* fetal livers were pooled before seeding. 1–4 × 10^5^ cells/ml purified erythroid progenitors were seeded in differentiation medium (StemPro‐34 SFM media and 1× Nutrient supplement (Gibco), 2 mM L‐Glutamine (Gibco), 1% Penicillin‐Streptomycin (Gibco), 0.1 mM β‐Mercaptoethanol (31350, Gibco), 20% BIT 9500 Serum Substitute (Stem Cell Technologies), and 2 U/ml EPO (587102, BioLegend, in PBS/1% BSA) (A0281, Merck)) on fibronectin‐coated wells or cover slips (CS) and cultured at 5% CO_2_ and 37°C. After 24 h, media were replaced by fresh differentiation medium without EPO and cells were cultured for up to 48 h.

#### Fibronectin coating

Plates or CS were coated with 1.3–1.5 µg/cm^2^ human fibronectin (354008, Corning). Fibronectin was diluted in PBS, added to wells, and incubated for 1 h at RT. Fibronectin solution was aspirated and wells/CS washed once with PBS. PBS was aspirated and plates were stored at 4°C until further use (not longer than 2 weeks).

#### Drug treatment

Erythroid progenitors were seeded in centrinone‐B (Tocris, 5690) and the drug was re‐added, if necessary, with media exchange after 24 h. Cells were collected at the indicated time points and processed for respective analyses. For nocodazole treatment, erythroblasts were treated with 10 µM nocodazole (M1404, Merck) at T34 for 2 h.

### Generation of mouse embryonic fibroblasts (MEFs)

MEFs were generated as previously described (Jozefczuk *et al*, 2012) with the following adjustments: digestions were carried out with gentle rotation and DMEM GlutaMAX (Gibco) supplemented with 10% heat‐inactivated FBS (Thermo Fisher Scientific) and 1% Penicillin–Streptomycin (Gibco) was used as MEF medium. MEFs were used at passages 2–4 for experiments.

### Immunoblotting and intensity measurements

To collect cells at T24 and T30, the media were removed but collected and PBS/10%FBS/5 mM EDTA was added to lift the cells. Cells were resuspended and cell suspension was transferred into the corresponding tube. Each well was washed with PBS and PBS was transferred into the corresponding tube. To collect cells at T36 to T48, cells were resuspended and cell suspension was transferred into a tube. Each well was washed with PBS and PBS was transferred into the corresponding tube. Collected cells were washed twice with PBS to remove BSA. For Figs 1B and EV1C, cells were lysed in RIPA lysis buffer (150 mM NaCl, 1% NP40, 0.5% Na‐deoxycholate, 0.1% SDS, 50 mM Tris–HCl pH 8.0, 1 mM EDTA, and 1× cOmplete mini EDTA‐free Protease Inhibitor Cocktail (Roche)) for 20 min on ice. Cell lysates were cleared by centrifugation at 4°C for 15 min at 21,100 *g* and 1× NuPage LDS sample buffer including NuPage reducing agent was added. For crude lysate (Figs 7A, EV3F, G, J, L, and N, and EV6A), cell pellets were directly lysed in 1× NuPage LDS sample buffer containing NuPage reducing agent. Lysates were separated on Bolt 4–12% Bis‐Tris Mini Protein Gel (Thermo Fisher Scientific) using MOPS (B000102) or MES (B0002) SDS running buffer and transferred onto nitrocellulose membrane for immunoblot analysis.

For Fig EV1D, native gel electrophoresis was performed according to manufacturer’s protocol (NativePAGE Bis‐Tris Gel Manual, Thermo Fisher Scientific) with following adjustments; MEFs were lysed in 1× NativePAGE Sample Buffer, 1% digitonin, 2 mM MgCl_2_, 1 U/µl Benzonase (E1014, Merck), and 1× cOmplete mini EDTA‐free Protease Inhibitor Cocktail (Roche) for 30 min on ice. 1/10^th^ of G‐250 was used. Lysates were separated on NativePAGE 4–16% Bis‐Tris Mini Protein Gel (Thermo Fisher Scientific).

For immunoblot analyses, primary antibodies were used as follows: CDK5RAP2 (1:300 or 1:500, (Barr *et al*, 2010)), β‐actin (1:4,000, A1978, Merck), α‐tubulin (1:2,000, T9026, Merck) RB (1:250, 554136, BD), TP53 (1:250, 2524, CST), P27 (1:500, 610242, BD), P21 (1:300 or 1:500, 556431, BD), GAPDH (1:2,000, G8795, Merck), Phospho‐RB S807/811 (1:500, 8516, CST), cyclin D3 (1:500, 2936S, CST), cyclin A2 (1:1,000, ab32386, Abcam), cyclin B1 (1:500, 4135, CST), securin (1:1,400, 700791, Thermo Fisher Scientific), and mCherry (1:500, ab125096, Abcam). Horseradish peroxidase‐conjugated anti‐rabbit or anti‐mouse (1:3,000; GE Healthcare) was used as secondary antibodies.

Fiji was used to quantify the signal intensities and normalized to respective loading control.

### Immunofluorescence microscopy

Erythroid progenitors were seeded on fibronectin‐coated coverslips and fixed at indicated time points. MEFs were seeded on coverslips and fixed at confluency. Cells were either fixed with 4% methanol‐free formaldehyde (FA, 28908, Thermo Fisher Scientific) in PBS for 10 min at 37°C or 100% ice‐cold methanol (ACROS) for 5 min at −20°C. Fixed cells were washed once with PBS, permeabilized first with 0.1%Tween20/PBS for 5 min at RT and then with 0.5%TritonX‐100/0.5%Tween20/0.05%SDS in PBS for 3 min at RT, and blocked in 5% BSA (A9647, Merck) in PBS at 4°C. Primary antibodies were diluted in 5% BSA/PBS and incubated overnight at 4°C. Cells were washed three times for 10 min with 0.1%Tween20/PBS and then incubated with secondary antibodies diluted in 5% BSA/PBS for 1 h at 37°C. After washing three times for 10 min with 0.1%Tween20/PBS, DNA was stained with Hoechst 33342 (1:500, 1 mg/ml in RNase‐free water, Merck) in PBS for 10 min at RT. Cells were washed twice with PBS and once with Milli‐Q‐H_2_O. Coverslips were mounted onto glass slides in ProLong Diamond Antifade Mountant (Thermo Fisher Scientific).

For pHH3 staining in Figs 5, EV4, and EV5, the following adjustments were included: after secondary antibody staining, cells were washed as described, blocked with 5%BSA/PBS, and pHH3‐AF488 staining was performed in 5%BSA/PBS for 1 h at 37°C. Cells were washed and stained for DNA as described.

For Fig 1D and E, a cell suspension of 1 × 10^6^ erythroid progenitors/mL in PBS was prepared and 200 ul cell suspension was loaded into each Cytofunnel (A78710020, Thermo Fisher Scientific). Cells were cytocentrifuged onto glass slides with a Cytospin 4 cytocentrifuge (Thermo Fisher Scientific) at 1,000 rpm, medium acceleration for 3 min. The area around cytocentrifuged cells was marked with ImmEdge Hydrophobic Barrier PAP pen (Vector Laboratories). For Fig EV4C, a single‐cell suspension of freshly isolated fetal liver cells of E14.5 embryos was prepared and 2–3 × 10^5^ fetal liver cells were cytocentrifuged as described above. Fixation in methanol, permeabilization, and staining were performed as described above.

For immunofluorescence staining, the following antibodies were used: CDK5RAP2 (1:500, (Barr *et al*, 2010)), γ‐tubulin (1:1,000, T6557, Merck), CP110 (1:200, 12780‐1‐AP, Proteintech), CEP135 (1:300, ab75005, Abcam), PCNT (1:300–1:500, ab4448, Abcam), TER119‐FITC (1:200, 11‐5921‐82, eBioscience or 555915, BD), α‐tubulin (1:1,000, MCA786G, BioRad), CEP192 (1:300, kindly provided by R. Basto, Institute Curie, Paris, France), and pHH3‐AF488 (1:200, 3465, CST). Highly cross‐absorbed (rabbit and mouse) and cross‐absorbed (rat) AlexaFluor secondary antibodies (Thermo Fisher Scientific) were used. For imaging, a Plan‐Apochromat VC 100×/1.4 oil objective (Nikon) and 1.5× optovar were used. Z‐stacks with 0.3 μm or 0.5 μm distance were recorded on a Nikon Eclipse TE2000‐E inverted microscope with Neo 5.5 sCMOS camera (Andor) and pE‐300^White^ LED illumination system (CoolLED).

For STED staining and acquisition (Figs 2A and EV2A), cells were seeded on fibronectin‐coated high‐precision cover glasses (1.5 H, Paul Marienfeld) and fixed at the indicated time points. Cells were stained as described above with the following adjustments: both first and second antibodies were used at twice the concentration, AlexaFluor 568 and AlexaFluor 488 were used, and Hoechst staining was omitted. Cells were imaged using a Leica TCS SP8 3× gated STED confocal inverted microscope (Leica) coupled to a white light laser and three STED lines (592, 660, and 775 nm) and tunable spectral detectors. Images were acquired at a single focal plane using a HC PL APO 100×/1.4 oil STED WHITE objective.

### Image processing and quantification

Images were taken at identical exposure times within each experiment. They were imported into Fiji, converted into tiff files, and maximum intensity projections of z‐stacks were generated. To score centrosome number or spindle morphology, cells were counted using multipoint selection tool and assigned to the relevant category. For measuring signal intensities at the centrosome, a circle encompassing the centrosome signal was placed around each centrosome (marked by γ‐tubulin or CEP192). Raw integrated density was measured for required channels at the same position using the Fiji ROI manager. The measured intensity was divided by the area of the selection and the background was subtracted. STED images were deconvolved using Huygens Professional (Scientific Volume Imaging). To measure the signal diameter of centrosomes in STED images, a straight line across the dot or ring (at a position with similar intensities at opposite sites) was drawn. The full width half maxima (dot) or the distance between the intensity maxima (ring) was calculated using Fiji.

### Time‐lapse microscopy

Erythroid progenitors were seeded at 4–5 × 10^5^ cells/ml on fibronectin‐coated μl‐slide eight‐well ibiTreat (ibidi) and allowed to adhere for 1 h at 37°C, 5% CO_2_. Cells were imaged for 48 h at 37°C, 5% CO_2_ at a single focal plane in bright field using a Zeiss Axio observer Z1 inverted microscope equipped with an environmental chamber, and either an ORCA‐Flash 4.0 CMOS camera (Hamamatsu) and a Plan‐Apochromat 40×/0.95 Ph3 DIC objective (Zeiss) or an Axiocam 506 mono CCD camera (Zeiss) and a LD Plan‐Neofluar 40×/0.6 Ph2 DIC objective (Zeiss). Zen Blue software (Zeiss) was used for data collection and analysis. To quantify cell cycle timing, the duration between each cytokinesis was scored by following two daughter cells until they stopped dividing, died, or enucleated.

### ImageStream analysis and labeling experiments

To collect cells at T24 or T30, the media were removed but collected and PBS/10%FBS/5 mM EDTA was added to lift the cells. Cells were resuspended and cell suspension was transferred into corresponding tube. Each well was washed with PBS and PBS was transferred into the corresponding tube. To collect cells at T36 to T48, cells were resuspended and cell suspension was transferred into a tube. Each well was washed with PBS and PBS was transferred into the corresponding tube. Cells were pelleted by centrifugation for 5 min at 300 *g* and RT. The supernatant was removed and cells were fixed in 4% FA/PBS for 15 min at RT and washed once with PBS before they were stored in PBS/2% FBS at 4°C until further staining and imaging. Staining was performed in PBS/2% FBS for 20 min at RT or on ice. Stained cells were acquired on an ImageStream X Mark II Imaging Flow Cytometer (Amnis) using a 40× objective and controlled by INSPIRE software and fully ASSIST calibrated (Amnis). Single‐stained controls were collected with bright‐field (BF) illumination off but relevant excitation lasers on. Per sample 10,000 events per cell gate were recorded unless mentioned otherwise.

For ImageStream measurements, the following antibodies were used: TER119‐FITC (1:500, 11‐5921‐82, eBioscience or, 557915, BD), TER119‐APC (1:500, 116212, BioLegend), CD71‐PE (1:500, 553267, BD), cKit‐FITC (1:500, 11‐1171‐82, eBioscience), and pHH3‐AF488 (1:50, 3465, CST). DNA was either stained with Hoechst 33342 (1:500, 1 mg/ml in H_2_O, Merck) or Draq5 (1:5,000, 62254, Thermo Fisher Scientific). Fluorescence analysis was performed using IDEAS or FlowJo software. Analysis of the enucleating population was performed as described elsewhere (Konstantinidis *et al*, 2014).

#### PKH26‐labeling experiments

Erythroid progenitors were labeled with PKH26 red fluorescent cell linker kit (Merck) as described previously (Sankaran *et al*, 2012). Purified erythroid progenitors were washed once with pre‐warmed StemPro‐34 SFM medium (10639‐011, Thermo Fisher Scientific). Cells were resuspended in Diluent C and cell suspension was added to 4 μM PKH26 in Diluent C solution. Cells were resuspended and incubated for 1 min at RT. The reaction was stopped by adding an equal volume of heat‐inactivated FBS (Thermo Fisher Scientific) and incubation for 1 min at RT. Cells were washed with StemPro‐34 SFM medium and resuspended in differentiation medium. Labeled cells were seeded at 1–2 × 10^5^ cells/ml and cultured as described above. Subsequently, an aliquot of labeled erythroid progenitors was used to measure the PKH26 mean fluorescence intensity (MFI) at T0 and the number of cell divisions was calculated based on PKH26 MFI as described previously (Sankaran *et al*, 2012).

Labeled cells were collected at indicated time points and either stained for TER119‐APC, Hoechst, and 7AAD (5 μl/1 × 10^6^ cells, 420404, BioLegend) and analyzed immediately by LSRII flow cytometer (BD Pharmingen) (Fig 2B) or fixed, stained for TER119‐FITC and Draq5 and analyzed on ImageStream X as described above (Figs 4H and EV6D).

#### BrdU‐labeling experiments

*Ex vivo* cultured erythroblasts were pulsed with 10 μM BrdU for 30 min at T48 (Figs 3B, 4E and 7C) or 4 h at T44 (Fig 3H). Erythroblasts were collected at the indicated time points and BrdU incorporation was detected using the FITC BrdU Flow kit (BD Pharmingen) as described by the manufacturer’s protocol with the following adjustments: Pulsed cells were collected as described above and stained with TER119‐APC (1:250) in PBS/2%FBS for 15 min at RT. PBS/2%FBS was used as staining buffer. For short‐term storage, cells were resuspended in PBS/2%FBS and stored overnight at 4°C or washed once with PBS/2% FBS before staining was continued. Cells were treated with 300 μg/ml DNAse for 15 min at 37°C. Cells were stained for BrdU (1:77) and TER119‐APC (1:250) in PBS/2%FBS for 30 min on ice. After washing, stained cells were resuspended in PBS/2% FBS plus Hoechst (1:250) to stain for DNA and acquired using ImageStream X as described above.

#### Annexin V staining

*Ex vivo* cultured erythroblasts were collected at the indicated time points and stained for annexin V using Annexin V, Alexa Fluor 488 conjugate, and Annexin Binding Buffer (Thermo Fisher Scientific). Collected cells were first washed in ice‐cold PBS and then in ice‐cold 1× Annexin Binding buffer. Washed cells were resuspended in Annexin V staining solution (1:20 Annexin V Alexa Fluor 488 in 1× Annexin Binding Buffer) and incubated for 30 min at RT. Subsequently, the cells were washed with 1× Annexin Binding Buffer and pelleted by centrifugation. The cells were fixed in 2% FA in 1× Annexin Binding Buffer for 15 min at RT and washed with PBS. The samples were stored in PBS/2%FBS at 4°C until further staining and imaging. Cells were stained for TER119‐APC and Hoechst in PBS/2%FBS for 20 min at RT or 30 min on ice, washed with PBS/2%FBS and pelleted by centrifugation. Cells were resuspended in PBS/2%FBS and acquired using ImageStream X as described above.

#### pHH3 staining

*Ex vivo* cultured erythroblasts were collected at the indicated time points as described above. Before fixation, cells were stained for cell surface marker TER119‐APC in PBS/2%FBS for 15 min at RT. After washing, cells were fixed in 4% FA/PBS as described above and stored in PBS/2%FBS overnight at 4°C. Fixed cells were permeabilized by adding 200 µl 1× eBioscience Permeabilization Buffer (00‐8333‐56, Thermo Fisher Scientific) and pelleted by centrifugation for 5 min at 300 *g*. Cells were stained for TER119‐APC, Hoechst, and pHH3‐Alexa Fluor 488 in 1× eBioscience Permeabilization Buffer for 20 min at RT. After washing, cells were resuspended in PBS/2%FBS and acquired using ImageStream X as described above. Per sample, 30,000–40,000 events per cell gate were collected. Analysis of mitotic stages was performed as described by Filby *et al* (2011).

### Isolation of bone marrow cells

Mouse femora and tibiae from both hind limbs were harvested and kept on ice. Muscles and connective tissues were removed using forceps and Kim wipes. For Fig EV1G and H, bone marrow cells were isolated according to the following procedure: bones were placed in PBS/2%FBS in a mortar and carefully cracked open using the pestle. Bone marrow cells were mechanically dissociated by passing them through 18G needle and dissociated cells were passed through a 70 µm cell strainer (Greiner) to prepare a single cell suspension. Cells were pelleted by centrifugation and resuspended in 1 ml PBS/2% FBS. For hematopoietic stem and progenitor cell staining, RBC lysis was performed using 10 ml 1× RBC Lysis Buffer (BioLegend) according to the manufacturer's protocol. For Fig EV1H, bone marrow cells were isolated as described by Amend *et al* (2016) using only one femur and tibia. Bone marrow cells were resuspended in PBS and passed through a 70 µm cell strainer (Greiner) to prepare a single‐cell suspension. For Fig EV1G and H, bone marrow cells were stained and analyzed by flow cytometry as described below. For Fig EV1J, bone marrow cells were fixed, stained for CD71‐PE, TER119‐APC, cKit‐FITC, and Hoechst, and analyzed on ImageStream X as described above.

### Flow cytometry (FACS) analysis

For hematopoietic stem and progenitor cell staining in Fig EV1G, bone marrow cells were stained with mature lineage cocktail FITC (B220, CD3, CD4, CD8, Mac1, GR1, CD11c, FceRI, and TER119), CD41/CD48‐biotin, CD150‐PE, cKit‐PerCEP‐Cy5.5, and Sca1‐PE‐Cy7 in PBS/2% FBS for 30 min at RT. Secondary staining of CD41/CD48 biotin was performed in PBS/2% FBS for 30 min at RT using Streptavidin‐BV421. Stained cells were analyzed immediately by LSRII flow cytometer (BD Pharmigen) with 10^6^ events recorded. For erythroid cell staining in Fig EV1H, bone marrow cells were labeled with mature lineage cocktail FITC (B220, CD3, CD41, Mac1, and GR1), CD71‐PE, and TER119‐APC as described above. Streptavidin‐AlexaFluor488 was used as secondary staining for CD41 biotin and DNA was stained with Hoechst. After 7AAD staining (5 μl/1 × 10^6^ cells, 420404, BioLegend), cells were analyzed immediately by LSRII flow cytometer (BD Pharmingen) with 150.000 events recorded. For Fig EV1I, freshly isolated fetal liver cells were stained with CD71‐PE (1:1,000, 553267, BD), TER119‐APC (1:1,000, 553267, BD), and Hoechst and analyzed after 7AAD staining as described above.

The following antibodies were used for flow cytometry staining: B220‐FITC (1:300, 561877, BD), CD3e‐FITC (1:300, 11‐0031‐81, eBioscience), CD4‐FITC (1:300, 561831, BD), CD8a‐FITC (1:300, 561966, BD), Mac1‐FITC (1:300, 561688, BD), GR1‐FITC (1:300, 11‐5931‐81, eBioscience), CD11c‐FITC (1:300, 11‐0114‐81, eBioscience), FcεRIα‐FITC (1:300, 11‐5898‐81, eBioscience), TER119‐FITC (1:300, 561032, BD), CD41‐biotin (1:400, 13‐0411‐81, eBioscience), CD48‐biotin (1:400, 103409, BioLegend), CD150‐PE (1:200, 115903, BioLegend), cKit‐PerCEP‐Cy5.5 (1:200, 105823, BioLegend), Sca1‐PE‐Cy7 (1:200, 25‐5981‐81, eBioscience), CD71‐PE (1:200, 553267, BD), TER119‐APC (1:200, 116212, BioLegend), Streptavidin‐BV421 (1:200, 405226, BioLegend), and Streptavidin‐AlexaFluor 488 (1:200, 405235, Biolegend). DNA was stained with Hoechst 33342 (1:500, 1 mg/ml in H_2_O, Merck). Fluorescence analysis was performed using FlowJo. Gating for hematopoietic stem and progenitor cells was performed as described by Oguro *et al* (2013) and for erythroblast stages as described by Koulnis *et al* (2011).

### Statistics

Sample sizes (number of cells embryos or adult mice) are indicated in each figure legend. Experiments were neither blinded nor randomized but FACS and ImageStream data of fetal liver‐derived *ex vivo* cultures were collected simultaneously for each embryonic liver of a litter using identical gating strategies and often without prior knowledge of genotypes. Time‐lapse and confocal microscopy analyses were not blinded. GraphPad Prism 8.0 was used for all statistical analyses. To assess differences between multiple groups and one or more variables, we used ANOVA test with Tukey multiple comparison. Note that Prism performs Tukey–Kramer test, allowing for unequal sample sizes. Data with non‐normal distribution were analyzed with non‐parametric Mann–Whitney *U* test or Kruskal–Wallis test with Dunn's multiple comparisons as indicated. The statistical tests that were used in each graph are described in the corresponding figure legends. *P ≤ 0.05, ***P ≤ 0.01, ***P ≤ 0.001, ****P ≤ 0.0001.

## Author contributions

**Fanni Gergely:** Conceptualization; Resources; Supervision; Funding acquisition; Writing—original draft; Project administration; Writing—review & editing. **Péter Tátrai:** Conceptualization; Resources; Formal analysis; Investigation; Writing—review & editing.

In addition to the CRediT author contributions listed above, the contributions in detail are:

PT and FG conceived the study, and FG wrote the manuscript with input from PT. Experiments were performed by PT.

## Disclosure and competing interests statement

The authors declare that they have no conflicts of interest.

## Supporting information



Expanded View Figures PDF

Movie EV1

## Data Availability

This study includes no data deposited in external repositories.
