# Supplementary material for: Centrosome function is critical during terminal erythroid differentiation
Source: EMBO J. 2022 Jun 9;41(14):e108739. doi: 10.15252/embj.2021108739 (PMC9289712; doi:10.15252/embj.2021108739)
Supplement: Supplementary file 1 — Expanded View Figures PDF [file EMBJ-41-e108739-s001.pdf]

Expanded View Figures

Figure EV1. Hematopoietic progenitor pools are largely normal in *Cdk5rap2*^{null} mice.

A Immunoblot showing the detection of mCherry-tagged IVT. Ponceau-S staining was used to compare equal loading. 7* marks the alternative start site in exon 7.
B Schematic representation showing the antigen and the epitope of CDK5RAP2 N-terminal antibody as suggested from IVT experiments in (A).
C Immunoblot showing CDK5RAP2 levels in *Cdk5rap2 wild-type* (*WT*) and *null* mouse embryonic fibroblasts (MEFs). Actin was used as loading control. ** indicates unspecific band.
D Immunoblot of native gel showing CDK5RAP2 levels in *Cdk5rap2 wild-type* (*WT*) and *null* mouse embryonic fibroblasts (MEFs). Tubulin was used as loading control.
E Immunofluorescence images of interphase (I) or mitotic (M) *Cdk5rap2 WT* and *null* mouse embryonic fibroblasts (MEFs). MEFs were stained for CDK5RAP2 (grey), γ-tubulin (magenta), and DNA (Hoechst, blue). Images are maximum-intensity projections of deconvolved z-stacks. Scale bar, 4 µm. Insets show higher magnification of centrosomes. Scale bar, 500 nm.
F Quantification of mean centrosomal signal intensities of CDK5RAP2 from (E). Numbers in brackets correspond to number of MEF lines analyzed with 168 (*WT*) and 154 (*null*) interphase cells and 59 (*WT*) and 56 (*null*) mitotic cells.
G, H Quantification of hematopoietic stem and progenitor cells (G) and erythroblast stages (H) in bone marrow (BM) of 10- to 13-week-old mice. Genotypes are as indicated. The number in brackets refers to the number of mice analyzed. HSC = hematopoietic stem cells. MPP = multipotent hematopoietic progenitors. HPC = hematopoietic progenitor cells.
I Quantification of erythroblast stages in E13.5 fetal livers. Genotypes are as indicated. The number in brackets refers to the number of embryos analyzed.
J Quantification of cell size of TER119^{pos} cells in bone marrow (BM) of 10-week-old mice. Genotypes are as indicated. The number in brackets refers to the number of mice analyzed.

Data information: Box plots show 5th and 95th (whiskers) and 25th, 50th, and 75th percentiles (boxes). Bar graph in J displays mean ± s.d. Statistical analysis was based on the number of MEF lines (F), the number of mice (G, H, and J), or the number of embryos (I). Statistical significance was determined by one-way ANOVA with Tukey's multiple comparisons test (F and I), Mann–Whitney test (G and H), or two-tailed unpaired Student's *t*-test (J). $^{**}P \leq 0.01$.

 © 2022 The Authors

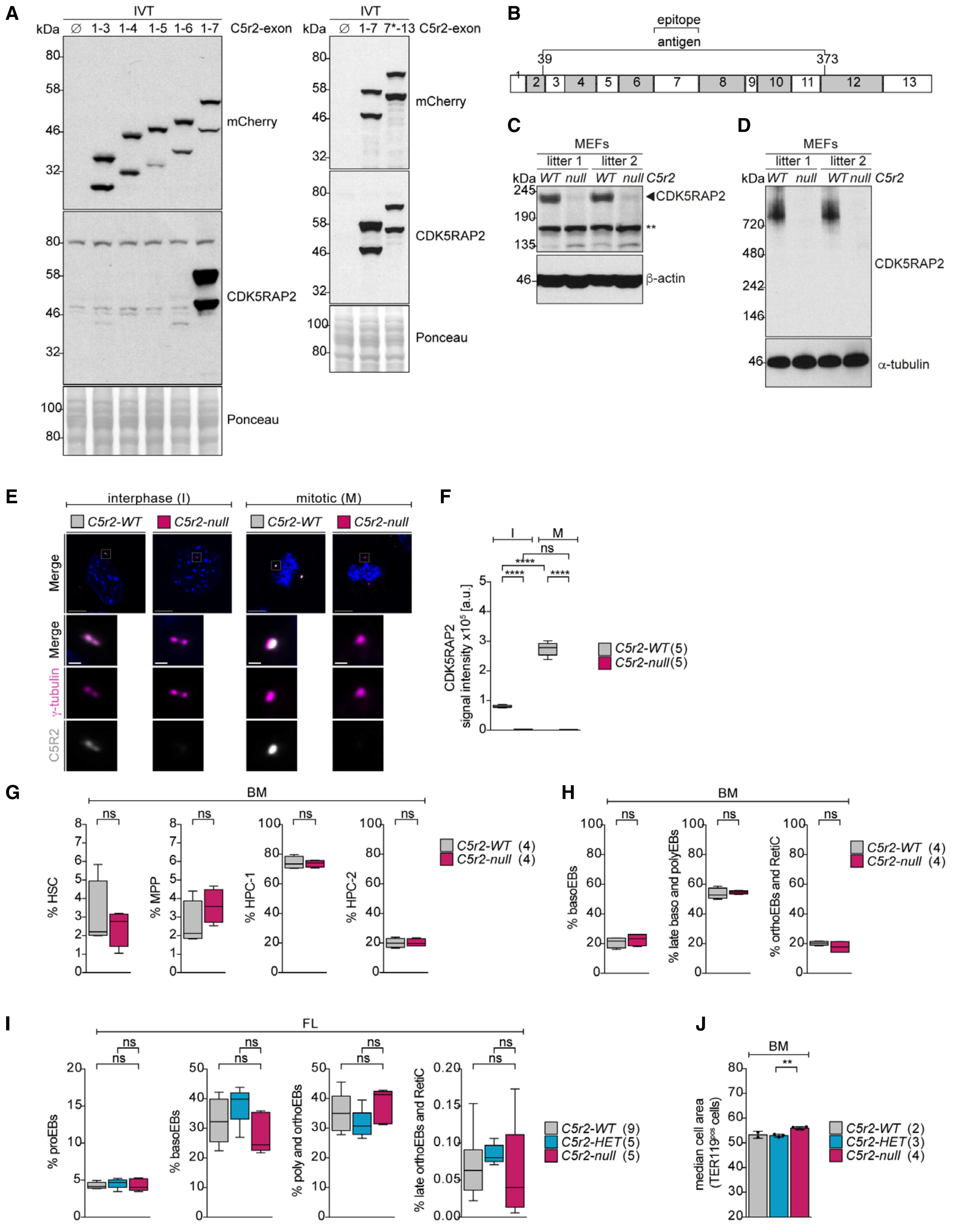
A
IVT
kDa
Ø
1-3
1-4
1-5
1-6
1-7
C5r2-exon
mCherry
CDK5RAP2
Ponceau
7*-13
B
epitope
antigen
39
373
C
MEFs
litter 1
litter 2
WT
null
C5r2
CDK5RAP2
β-actin
D
α-tubulin
E
interphase (I)
mitotic (M)
C5r2-WT
C5r2-null
Merge
γ-tubulin
C5R2
F
I
M
ns
CDK5RAP2 signal intensity x10^5 [a.u.]
C5r2-WT(5)
C5r2-null(5)
G
BM
% HSC
% MPP
% HPC-1
% HPC-2
C5r2-WT (4)
C5r2-null (4)
H
% basoEBs
% late baso and polyEBs
% orthoEBs and RetiC
I
FL
% proEBs
% poly and orthoEBs
% late orthoEBs and RetiC
C5r2-WT (9)
C5r2-HET(5)
C5r2-null (5)
J
median cell area (TER119pos cells)
C5r2-WT (2)
C5r2-HET(3)
C5r2-null (4)

Figure EV1.

Figure EV2. Intact centrosomes are present in enucleating erythroblasts but their PCM is reduced in size.

A Quantification of signal diameters for centriolar (dot) and PCM proteins (ring) from Fig 2A. The numbers in brackets correspond to the number of centrosomes analyzed in one experiment.

B Immunofluorescence images of *ex vivo* cultured *wild-type* non-enucleating EBs (non-enucl.) or enucleating EBs/reticulocytes (enucl./RetiC). Cells were stained for γ-tubulin (magenta), protein of interest (POI, CDK5RAP2, PCNT, or CEP192 in grey), TER119 (erythroid marker, green), and DNA (Hoechst, blue). Scale bar, 2 μm.

C Quantification of mean centrosomal signal intensities of PCM proteins from (B). The numbers in brackets refer to the number of cells analyzed in one experiment.

D Quantification of centrosome number in *ex vivo* cultured non-enucleating and enucleating erythroblasts as well as reticulocytes. Four litters with a total number of 2,618 (non-enucl.), 715 (enucl.), and 877 (RetiC) cells were analyzed.

Data information: Box plots show 5th and 95th (whiskers) and 25th, 50th, and 75th percentiles (boxes). Statistical analysis was based on the number of centrosomes (A), the number of cells (C), or the number of litters (D). Statistical significances were determined by Mann–Whitney test (A and C) or One-way ANOVA with Tukey's multiple comparisons test (D). *$P \leq 0.05$, **$P \leq 0.01$, ****$P \leq 0.0001$.

© 2022 The Authors

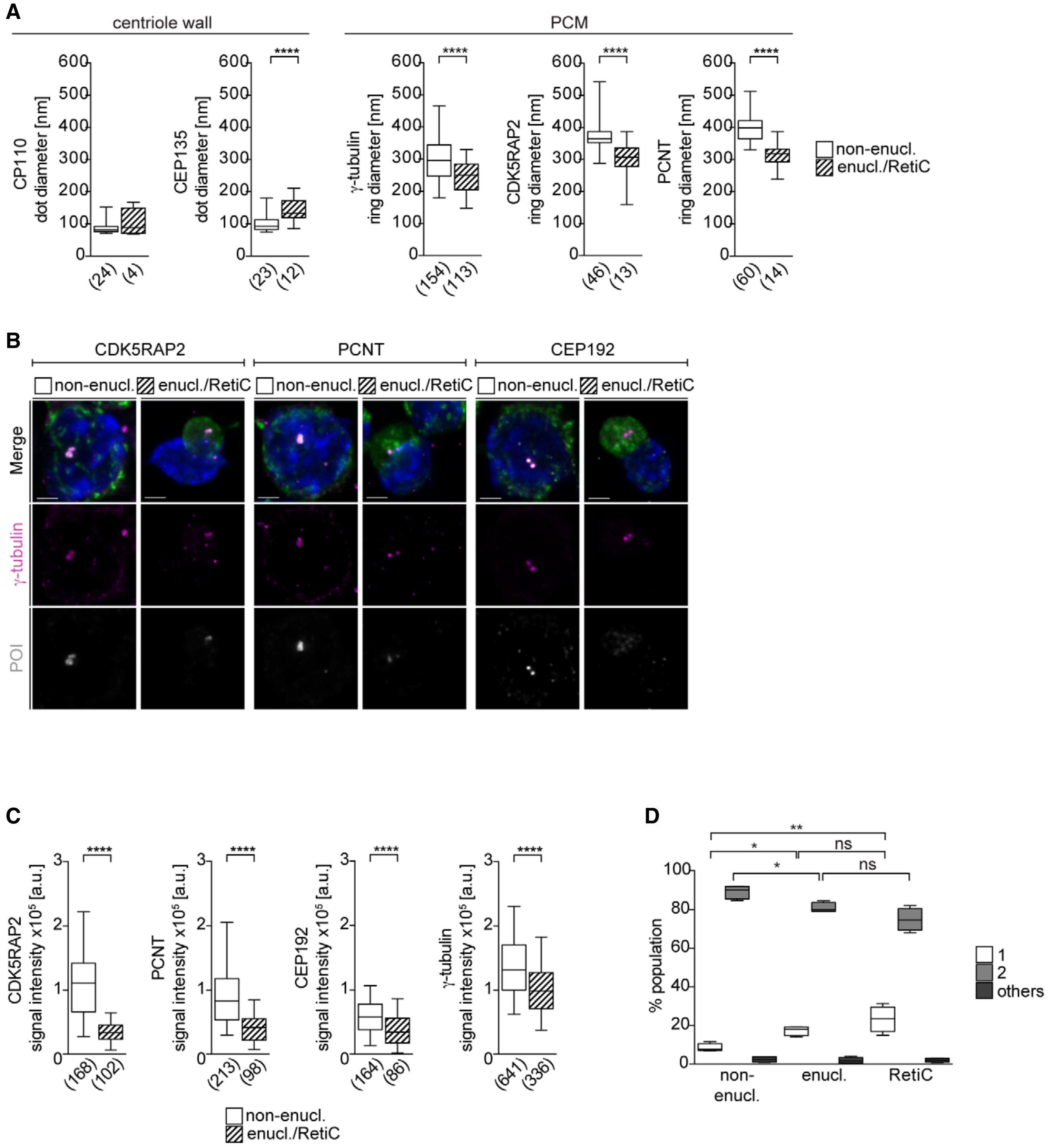
A
centriole wall
PCM
CP110 dot diameter [nm]
CEP135 dot diameter [nm]
γ-tubulin ring diameter [nm]
CDK5RAP2 ring diameter [nm]
PCNT ring diameter [nm]
****
(24) (4)
(23) (12)
(154) (113)
(46) (13)
(60) (14)
non-enucl.
enucl./RetiC
B
CDK5RAP2
PCNT
CEP192
Merge
γ-tubulin
POI
C
CDK5RAP2 signal intensity x10^5 [a.u.]
PCNT signal intensity x10^5 [a.u.]
CEP192 signal intensity x10^5 [a.u.]
γ-tubulin signal intensity x10^5 [a.u.]
(168) (102)
(213) (98)
(164) (86)
(641) (336)
D
% population
non-enucl.
enucl.
RetiC
**
*
ns
1
2
others

Figure EV2.

Figure EV3. Cell cycle analysis of *Cdk5rap2*^{null} erythroid progenitors during *ex vivo* differentiation.

A, B Quantification of *ex vivo* cultured BFU-E and CFU-E (A) as well as mature CFU-E (B) progenitor populations after 24 h (T24). Genotypes are as indicated. Number of embryos analyzed is shown in brackets.

C, D Quantification of apoptotic (AnnexinV^{pos}) TER119^{pos} cells at T24 (C) and T48 (D) of *ex vivo* culture. Genotypes are as indicated. Number of embryos analyzed is shown in brackets. T24 = 24 h. T48 = 48 h.

E Quantification of cell cycle profiles of *ex vivo* cultured enucleating EBs and TER119^{pos} cells after BrdU pulse for 30 min at 24 h (T24). Genotypes are as indicated. Number of embryos analyzed is shown in brackets.

F Immunoblot showing levels of cell cycle markers in *wild-type* erythroid progenitors after 24 (T24) and 48 (T48) hours of *ex vivo* culture.

G Immunoblot showing phospho-RB (S807/811) and cyclin A2 levels in *Cdk5rap2 wild-type* (WT), *heterozygous* (HET), and *null* erythroid progenitors at 48 h (T48) of *ex vivo* culture. GAPDH was used as loading control. ** indicates unspecific band.

H Quantification of mean protein levels from (G). Number of embryos analyzed is shown in brackets.

I Quantification of cell cycle profiles of *ex vivo* cultured TER119^{pos} cells upon CB treatment from Fig 4E. Number of litters analyzed is shown in brackets.

J Immunoblot showing phospho-RB (S807/811) and cyclin A2 levels in CB-treated erythroid progenitors at 48 h (T48) of *ex vivo* culture. GAPDH was used as loading control.

K Quantification of mean protein levels from (J). Number of litters analyzed is shown in brackets.

L Immunoblot showing phospho-RB (S807/811) and cyclin A2 levels in CB-treated erythroid progenitors at 24 (T24) and 48 (T48) hours of *ex vivo* culture. GAPDH was used as loading control.

M Quantification of mean protein levels from (L). Number of litters analyzed is shown in brackets.

N Immunoblot sowing cyclin B1 and securin levels in CB-treated erythroid progenitors at 48 h (T48) of *ex vivo* culture. GAPDH was used as loading control.

O Quantification of mean protein levels from (N). Number of litters analyzed is shown in brackets.

Data information: Box plots show 5th and 95th (whiskers) and 25th, 50th, and 75th percentiles (boxes). Bar graphs display mean ± s.d. Statistical analysis was based on the number of embryos (A-E) or number of litters (F and L). All statistical significances were determined by one-way ANOVA with Tukey's multiple comparisons test. $P \leq 0.05$, $**P \leq 0.01$, $***P \leq 0.001$, $****P \leq 0.0001$.

© 2022 The Authors

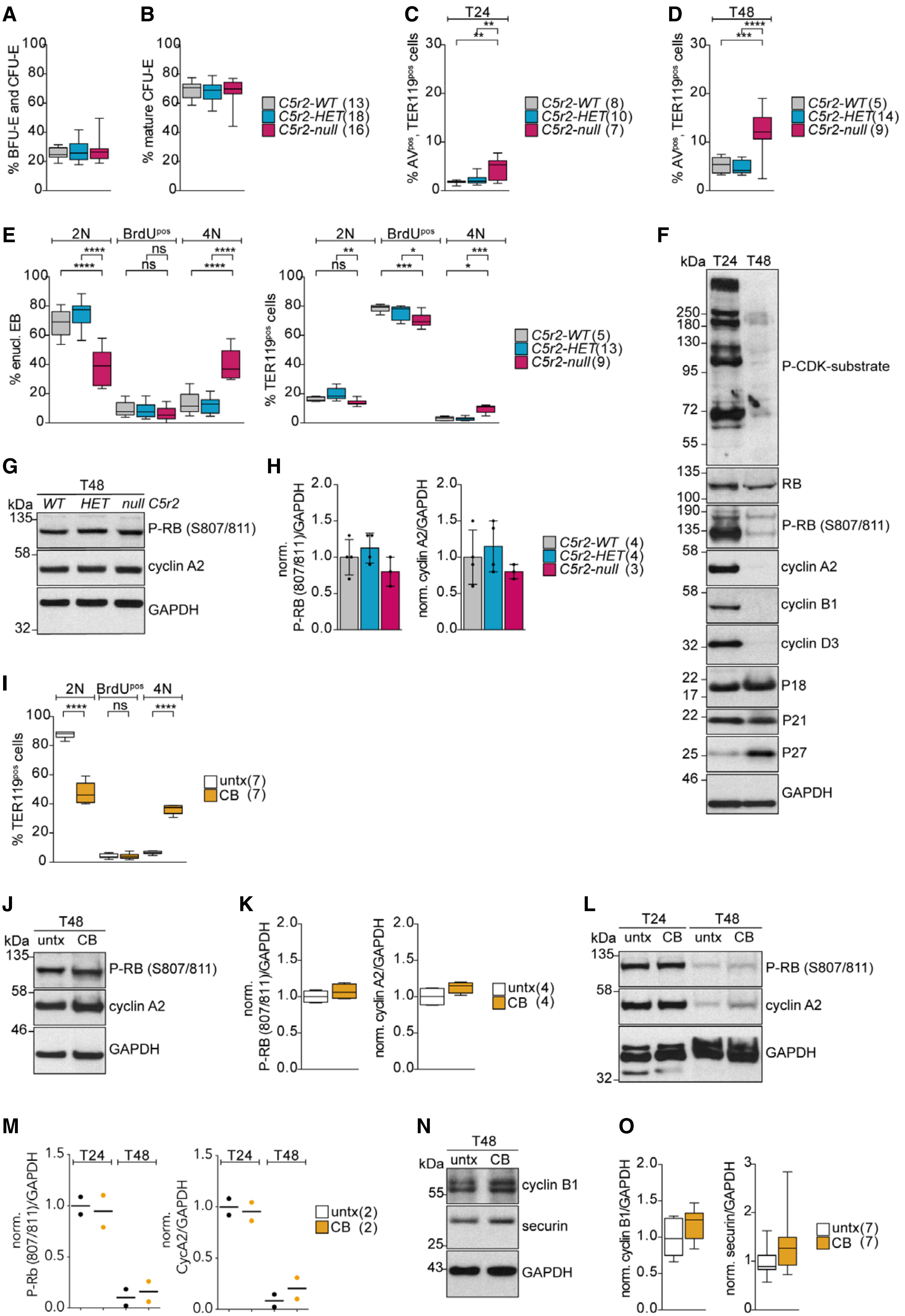
A
B
C
D
E
F
G
H
I
J
K
L
M
N
O
C5r2-WT (13)
C5r2-HET (18)
C5r2-null (16)
T24
T48
P-CDK-substrate
RB
P-RB (S807/811)
cyclin A2
cyclin B1
cyclin D3
P18
P21
P27
GAPDH
securin
untx
CB

Figure EV3.

Figure EV4. Mitotic spindle morphology in erythroid progenitors lacking centrosomes or CDK5RAP2.

A Immunofluorescence images of mitotic cells (untx and CB) with different spindle morphologies at 24 h (T24) of *ex vivo* culture. Cells were stained for α-tubulin (magenta), γ-tubulin (grey), pHH3 (green), and DNA (Hoechst, blue). Images are maximum-intensity projections of deconvolved z-stacks. Scale bar, 2 μm. See Fig 5B for quantification of spindle morphology.

B Immunofluorescence images of *Cdk5rap2*^{null} mitotic cells with different spindle morphologies at 36 h (T36) of *ex vivo* culture. Cells were stained for α-tubulin (magenta), γ-tubulin (grey), pHH3 (green), and DNA (Hoechst, blue). Images are maximum-intensity projections of deconvolved z-stacks. Scale bar, 2 μm. See Fig 5D for quantification of spindle morphology.

C Quantification of spindle morphology of mitotic *Cdk5rap2 WT*, *HET*, and *null* cells freshly isolated from E14.5 fetal liver (FL). One *Cdk5rap2*^{WT} embryo (72 mitotic cells), three *Cdk5rap2*^{HET} embryos (192 mitotic cells), and two *Cdk5rap2*^{null} embryos (147 mitotic cells) were analyzed.

D Immunofluorescence images mitotic mouse embryonic fibroblasts (MEFs) with different spindle morphologies. MEFs were stained for α-tubulin (magenta), γ-tubulin (grey), pHH3 (green), and DNA (Hoechst, blue). Images are maximum-intensity projections of deconvolved z-stacks. Scale bar, 4 μm.

E Quantification of spindle morphology in mitotic *Cdk5rap2 WT* and *null* mouse embryonic fibroblasts (MEFs) from (C). Per genotype 5 MEF lines with 58 (WT) or 70 (null) mitotic cells were analyzed.

Data information: Bar graphs display mean ± s.d.

 © 2022 The Authors

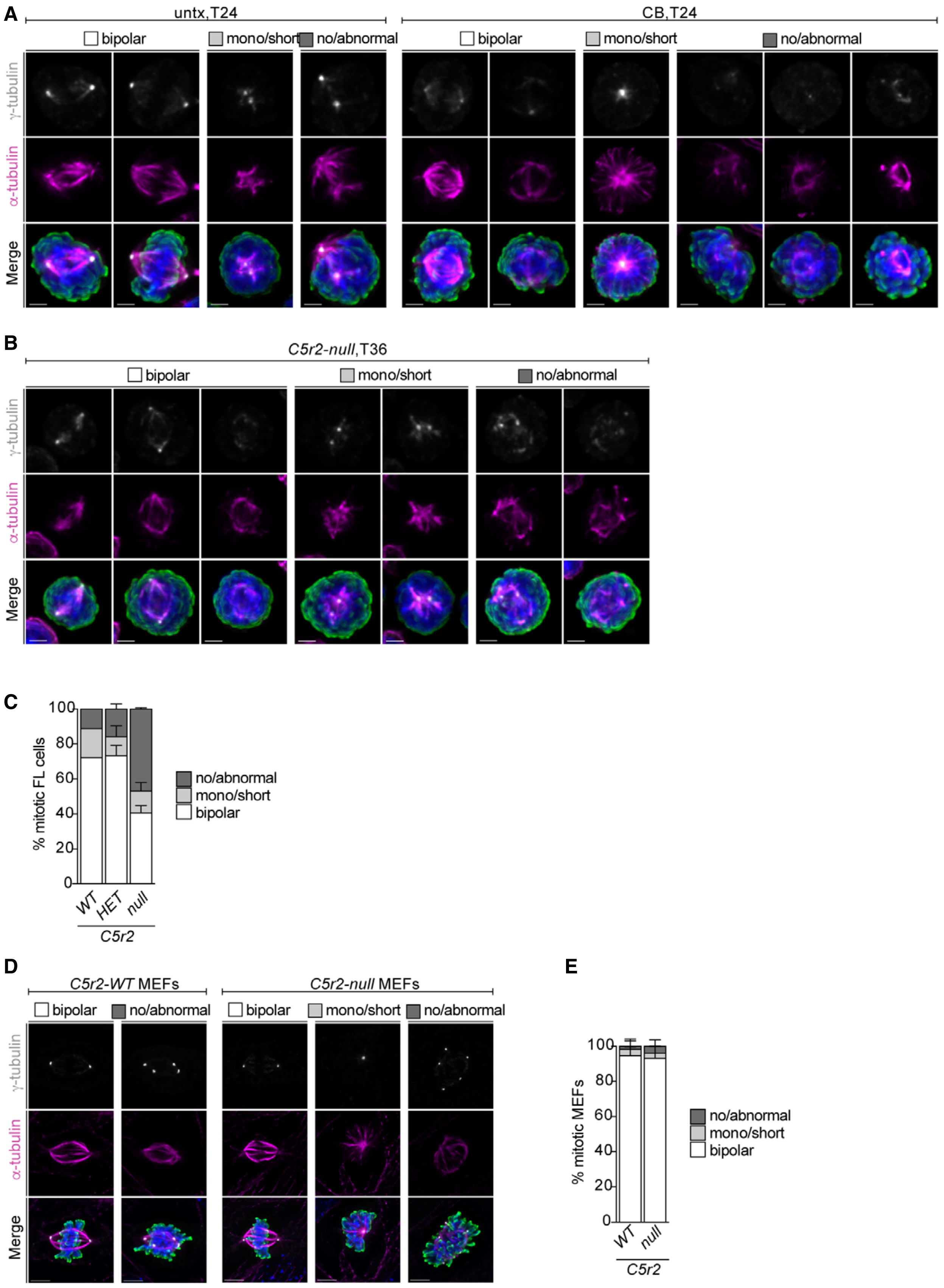
A
untx,T24
CB,T24
bipolar
mono/short
no/abnormal
γ-tubulin
α-tubulin
Merge
B
C5r2-null,T36
bipolar
mono/short
no/abnormal
C
% mitotic FL cells
100
80
60
40
20
0
WT
HET
null
C5r2
no/abnormal
mono/short
bipolar
D
C5r2-WT MEFs
C5r2-null MEFs
bipolar
mono/short
no/abnormal
E
% mitotic MEFs
WT
null
C5r2

Figure EV4.

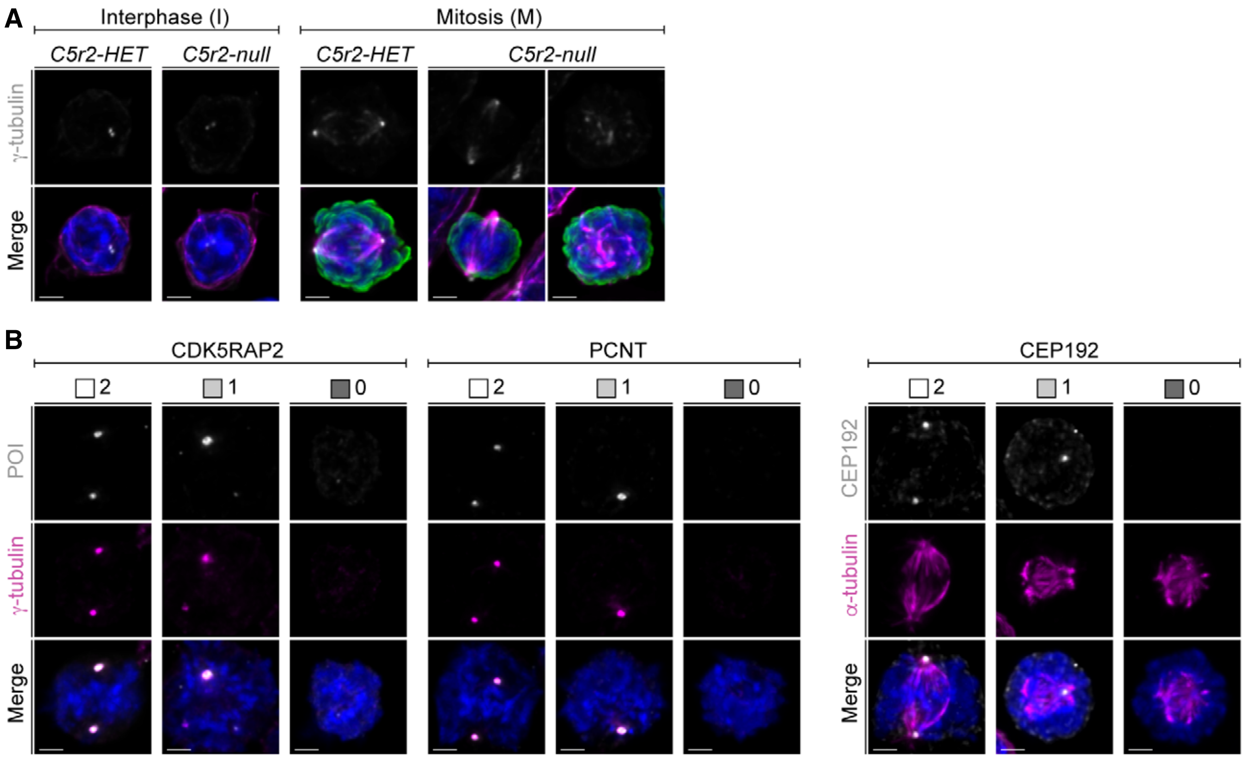

Figure EV5. Effect of centrinone treatment on PCM foci formation in erythroid progenitors during *ex vivo* differentiation.

A Representative immunofluorescence images of *ex vivo* cultured *Cdk5rap2 HET* and *null* cells in interphase (I) and mitosis (M). Cells were stained for α-tubulin (magenta), γ-tubulin (grey), pHH3 (green), and DNA (Hoechst, blue). Images are maximum-intensity projections of deconvolved z-stacks. Scale bar, 2 μm. See Fig 6F for quantification of mean centrosomal signal intensities of γ-tubulin.

B Representative immunofluorescence images of *ex vivo* cultured mitotic cells (untx and CB) with different PCM foci numbers. Cells were stained for γ-tubulin or α-tubulin (magenta), protein of interest (POI, CDK5RAP2, PCNT, or CEP192 in grey), and DNA (Hoechst, blue). Images are maximum-intensity projections of deconvolved z-stacks. Scale bar, 2 μm. See Fig 6G for quantification of PCM foci numbers.

Figure EV6. Terminal erythroid differentiation is impaired in *Trp53* and *Cdk5rap2* double mutants both *ex vivo* and *in vivo*.

A Immunoblot showing levels of TP53, P27, and P21 *ex vivo* cultured erythroid progenitors upon CB treatment. GAPDH was used as loading control.

B Quantification of mean protein levels from (A). Numbers in brackets represent number of embryos analyzed.

C Quantification of cell cycle profiles of *ex vivo* cultured *Trp53*^{HET} or *Trp53*^{null} TER119^{pos} cells following CB treatment from Fig 7C. Numbers in brackets represent number of embryos analyzed.

D Quantification of number of cell divisions at 48 h (T48) of *ex vivo* culture following CB treatment. *Ex vivo* cultured *Trp53*^{HET} or *Trp53*^{null} EBs were labeled with PKH26 to measure cell divisions. Number of embryos analyzed is shown in brackets.

E Quantification of number of cell divisions of CB-treated enucleating EBs with indicated DNA content from (D). Percentage of enucleating EBs in each category is shown below X-axis. Number of embryos analyzed is shown in brackets.

F Quantification of cell cycle profiles of *ex vivo* cultured TER119^{pos} cells from Fig 7E. Number of embryos analyzed is shown in brackets.

G Quantification of *ex vivo* cultured apoptotic (AnnexinV^{pos}) TER119^{pos} cells with indicated genotypes after 48 h (T48). Number of embryos analyzed is shown in brackets.

H Complete blood count analysis from adult mice with genotypes as indicated. The number of mice analyzed is shown in brackets. RBC = red blood cell. MCV = mean corpuscular volume.

Data information: Box plots show 5th and 95th (whiskers) and 25th, 50th, and 75th percentiles (boxes). Statistical analysis was based on the number of embryos (B-G) or number of mice (H). All statistical significances were determined by one-way ANOVA with Tukey's multiple comparisons test. *$P \leq 0.05$, **$P \leq 0.01$, ***$P \leq 0.001$, ****$P \leq 0.0001$.

▶

 © 2022 The Authors

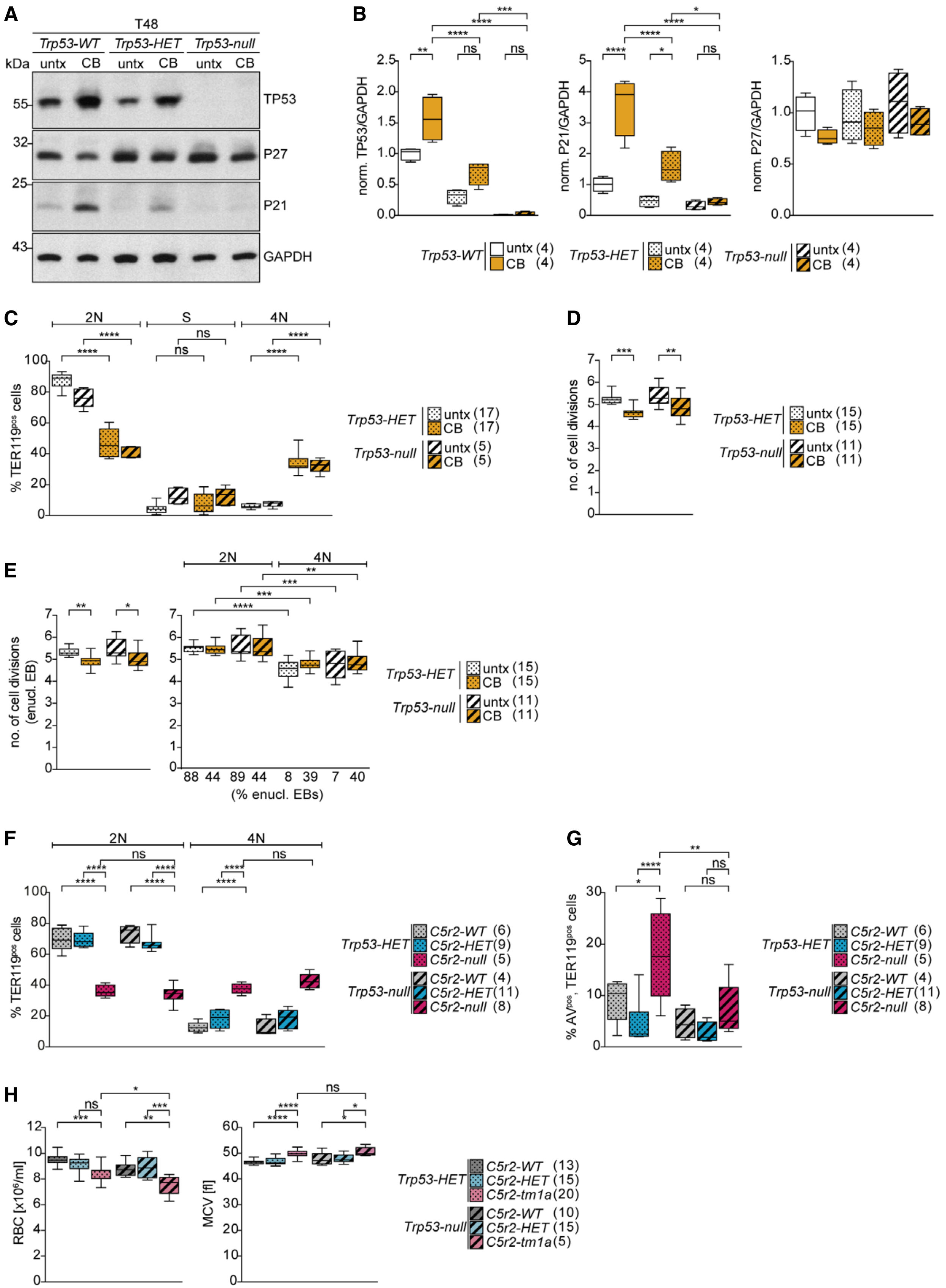
A
T48
Trp53-WT Trp53-HET Trp53-null
kDa untx CB untx CB untx CB
55
32
25
43
TP53
P27
P21
GAPDH
B
norm. TP53/GAPDH
norm. P21/GAPDH
norm. P27/GAPDH
Trp53-WT untx (4) CB (4)
Trp53-HET untx (4) CB (4)
Trp53-null untx (4) CB (4)
C
2N S 4N
% TER119pos cells
Trp53-HET untx (17) CB (17)
Trp53-null untx (5) CB (5)
D
no. of cell divisions
Trp53-HET untx (15) CB (15)
Trp53-null untx (11) CB (11)
E
no. of cell divisions (enucl. EB)
2N 4N
88 44 89 44 8 39 7 40
(% enucl. EBs)
Trp53-HET untx (15) CB (15)
Trp53-null untx (11) CB (11)
F
2N 4N
% TER119pos cells
Trp53-HET C5r2-WT (6) C5r2-HET(9) C5r2-null (5)
Trp53-null C5r2-WT (4) C5r2-HET(11) C5r2-null (8)
G
% AVpos, TER119pos cells
Trp53-HET C5r2-WT (6) C5r2-HET(9) C5r2-null (5)
Trp53-null C5r2-WT (4) C5r2-HET(11) C5r2-null (8)
H
RBC [x10⁶/ml]
MCV [fl]
Trp53-HET C5r2-WT (13) C5r2-HET (15) C5r2-tm1a(20)
Trp53-null C5r2-WT (10) C5r2-HET (15) C5r2-tm1a(5)

Figure EV6.
