# Supplementary material for: Centrosome function is critical during terminal erythroid differentiation
Source: EMBO J. 2022 Jun 9;41(14):e108739. doi: 10.15252/embj.2021108739 (PMC9289712; doi:10.15252/embj.2021108739)
Supplement: Supplementary file 2 — Movie EV1 [file EMBJ-41-e108739-s002.zip › Movie/LegendMovie.docx]

**Movie EV1:**

Tracking of erythroblast behavior for 48 hours. Movie shows cells in brightfield, the time interval is 3 minutes per frame at 100 frames per second. Time stamp is set to 00:00 hours at the start of the movie.
