# Supplementary material for: Centrosome function is critical during terminal erythroid differentiation
Source: EMBO J. 2022 Jun 9;41(14):e108739. doi: 10.15252/embj.2021108739 (PMC9289712; doi:10.15252/embj.2021108739)
Supplement: Supplementary file 3 [file EMBJ-41-e108739-s003.pdf]

THE EMBO JOURNAL

Centrosome function is critical during terminal erythroid differentiation

Fanni Gergely and Péter Tátrai
DOI: 10.15252/embj.2021108739

Corresponding author(s): Fanni Gergely (fanni.gergely@bioch.ox.ac.uk)

Review Timeline:

Submission Date:	18th May 21
Editorial Decision:	26th May 21
Appeal to editorial rejection:	27th May 21
Editorial Correspondence:	2nd June 21
Editorial Decision:	15th Jul 21
Appeal to post-review rejection:	22nd July 22
Editorial Decision:	3rd Sept 22
Revision Received:	3rd May 22
Accepted:	25th May 22

Editor: Hartmut Vodermaier & Ieva Gailite

Transaction Report:

(Note: With the exception of the correction of typographical or spelling errors that could be a source of ambiguity, letters and reports are not edited. Depending on transfer agreements, referee reports obtained elsewhere may or may not be included in this compilation. Referee reports are anonymous unless the Referee chooses to sign their reports.)

1st Editorial Decision 26th May 2021

Thank you for submitting your manuscript "Centrosome function is critical during terminal erythroid differentiation" for consideration by The EMBO Journal. I have taken over the handling of your manuscript, since Hartmut is away from the office at the moment. I would also like to sincerely apologise for the delay in the assessment of your manuscript due to the currently high manuscript submission rate to our journal. I have now read your study carefully and discussed it with my colleagues. After these discussions, I am sorry to say that we have decided not to pursue the publication of the manuscript at The EMBO Journal.

We that your study reports on the role of Cdk5rap2 and centrosomes in regulation of erythroblast division and enucleation, leading to defects in erythrocyte development and macrocytic anemia in cdk5rap2 mutant mice. The findings further show that Cdk5rap2 is required for bipolar spindle formation in the erythroblasts, thus identifying a tissue-specific function of Cdk5rap2 in red blood cell development. Finally, Cdk5rap2 mutant phenotypes in erythroblast maturation and differentiation were independent of p53 activation. While we certainly appreciate the high quality of the study and the novelty of the described role of centrosomes and Cdk5rap2 in particular in the late steps of erythroblast maturation and differentiation, we also found that more detailed insights into the molecular details of the tissue-specific role of Cdk5rap2 in erythroblasts would be needed, especially since its more general function in red blood cell development was described in earlier work. Therefore, I am afraid we concluded your current manuscript is not a sufficiently strong candidate for peer review and publication in The EMBO Journal.

Thank you for giving us the opportunity to consider this manuscript. I am sorry that I could not offer better news this time, and I sincerely hope for rapid publication of your study at another venue.

Thank you for giving us the opportunity to submit a formal response to the points outlined in your decision letter regarding our submission EMBOJ-2021-108739. In particular, we would like to elaborate on two issues you highlighted as key deficiencies of our study and explain why we believe that our manuscript already addresses these. You said that (1) *the more general function of* Cdk5rap2 *in red blood cell development was* already *described in earlier work*, which then (2) necessitates *more detailed insights into the molecular details of the tissue-specific role of Cdk5rap2 in erythroblasts.*

Point 1: We assume you refer to the **Hertwig's mouse model**. Indeed, elegant work during the 1970s-90s on this classical anemia model revealed impaired proliferation and/or differentiation of committed erythroid progenitors. In particular, the authors observed a 10% increase in aneuploidy in metaphase chromosome spreads of bone marrow and fetal liver cells, and suggested that the **death of aneuploid cells during differentiation was responsible for the reduction in reticulocyte numbers**. However, they could not provide an explanation for macrocytosis (i.e. large reticulocytes), nor did they probe the underlying molecular cause of aneuploidy in the progenitors. This is not surprising as the authors did not know the gene locus in this mouse model originally obtained via mutagenesis. The exon inversion in *Cdk5rap2* was reported only in 2010 by the Walsh group (Lizarraga et al) who used this Hertwig anemia strain to study Cdk5rap2 in neurodevelopment.

An *ex vivo* differentiation system (not yet established in the 90s) enabled us to perform in-depth analysis of the roles of Cdk5rap2 and centrosomes during terminal erythroid differentiation. We demonstrated frequent failure of mitotic spindle assembly in Cdk5rap2-deficient erythroblasts along with a dramatic rise of tetraploidy prior to enucleation. Tetraploidy appears in late-stage erythroblasts and not in the committed progenitors and is therefore unlikely to be linked to proliferative defects or aneuploidy of the progenitors.
If cells exit from mitosis without chromosome segregation, this will lead to loss of a cell generation and thus fewer reticulocytes. The resulting tetraploidy is responsible not only for an increase in cell size but tetraploid cells are also impaired in enucleation, thus further decreasing reticulocyte production. **Therefore, our findings provide an explanation for the origin of both features of macrocytic anemia: insufficient number and increased size of red blood cells.**
We find that blocking apoptosis by p53 deletion in Cdk5rap2-deficient erythroblasts does not restore normal reticulocyte production or size. Therefore, our study is **NOT** an obvious extension of the previous body of work. Our results have led us to entirely different conclusions; **instead of apoptosis, spindle assembly defects and tetraploidy are the key drivers of the macrocytic anemia phenotype.**
Lastly, our manuscript is not solely about Cdk5rap2 – **we describe a physiological role for the centrosome during erythroid differentiation** as well, which has never been reported before.

Point 2: We believe our study provides ample mechanistic insights into the contributions of centrosomes and Cdk5rap2 to late erythroid differentiation.

Briefly, we uncovered **spindle assembly defects in Cdk5rap2-deficient erythroblasts but not MEFs** (**Figure 5**). Erythroblasts depleted of centrosomes display identical spindle defects at comparable frequency, implying that centrosomes are important for spindle formation in late-stage erythroblasts with the centrosome component Cdk5rap2 playing a crucial role. However, based on work from our lab and others', Cdk5rap2 is dispensable for bipolar spindle formation in vertebrate and mammalian cells. This poses the question as to **why erythroblasts need Cdk5rap2**.

In most cell types centrosomes are known to mature at the onset of mitosis by recruiting additional gamma tubulin complexes that facilitate microtubule assembly during spindle formation. Although Cdk5rap2 can bind and activate gamma-tubulin (Choi et al., JCB), previous studies including ours (e.g. Gavilan et al, EMBORep, Barr et al., JCB) suggest that its depletion has a minor impact on centrosomal gamma tubulin levels. Consistent with robust centrosome maturation, **centrosomal gamma tubulin levels increased 3-fold in mitotic MEFs** when compared to interphase, and **even Cdk5rap2-deficient MEFs displayed an over 2-fold increase** in centrosomal gamma tubulin levels (**Figure 6, EV1**). Strikingly**, wild-type erythroblasts did not show any sign of centrosome maturation** with gamma tubulin levels identical between interphase and mitosis. As a result, Cdk5rap2-deficient mitotic centrosomes contain even less gamma tubulin than centrosomes of wild-type erythroblasts in interphase. These data together suggest that Cdk5rap2 contributes to mitotic gamma tubulin recruitment both in erythroblasts and MEFs; however, it is only in erythroblasts where spindle assembly is affected in the absence of Cdk5rap2 or centrosomes. Why is that?

Our data demonstrate that centrosomes are vital for spindle assembly in erythroblasts, which means that gamma tubulin-driven centrosomal microtubule nucleation is particularly important for spindle formation in these cells. However, lack of centrosome maturation in erythroblasts means that these centrosomes depend on their interphase gamma tubulin reservoir to produce spindle microtubules. This reservoir in late-stage erythroblasts seems barely sufficient to supply microtubules as ~7-8% wild-type cells also show abnormal spindles (**Figure 5**)and thus loss of Cdk5rap2 could push gamma tubulin levels (and possibly activity) below the threshold required for effective centrosomal microtubule production, resulting in abnormal spindles. As detailed under point 1, spindle defects will lead to mitotic failure and tetraploidy, ultimately precipitating the reticulocyte phenotypes.

I want to highlight one last point that distinguishes our paper from previous studies. Recently EMBO J published an excellent study by the Holland lab, which demonstrated how centrosome dysfunction triggered a p53-dependent mitotic surveillance pathway *in vivo* and how it impacted neurodevelopment. While p53 is activated in late-stage erythroblasts lacking Cdk5rap2 (or centrosomes), phenotypes such as tetraploidy or defects in number/size of reticulocytes persist in p53/Cdk5rap2 double null animals, implying that these arise independently of the mitotic surveillance pathway. In mouse models of centrosome dysfunction, nearly all phenotypes are triggered by p53 activation, making erythroid differentiation all the more interesting.

I have now received external expert input on your manuscript EMBOJ-2021-108739R-Q, "Centrosome function is critical during terminal erythroid differentiation". I am glad to say that their comments were positive, and I am sending your study out for external peer review. I will be in touch again once I have received a full set of referee reports, which unfortunately might take a bit more time than usual under the current circumstances.

Editorial Decision 15th Jul 2021

Thank you again for submitting your manuscript on Cdk5rap2/centrosome roles in erythroid differentiation to The EMBO Journal. Since my colleague Ieva Gailite is now on maternity leave, I have taken over its handling from her, and am now sending you the reports we have in the meantime received from three referees with expertise in centrosome biology and erythropoiesis. As you will see, the referees appreciate your cell-biological findings, but at the same time raise concerns regarding the in vivo/hematological relevance of the present results. Since the latter would in our view represent one of the key advances to warrant EMBO Journal publication in this case, and since the crucial points (listed in particular by the erythropoiesis expert, referee 3) may not be easy to decisively address within a reasonable time frame, I unfortunately do not see myself in the position to invite (and thus to some extent commit to) a revision for The EMBO Journal at this stage.

While I would not exclude the possibility of looking at a new submission, addressing all the specific points of referees 1 and 2 as well as the more substantive concerns of referee 3, once more at a future point, I would however like to suggest an alternative option for publishing this work more rapidly and with less extensive modifications in our sister journal, EMBO Reports. As it stands, not being able to conduct in-depth investigation of in vivo erythropoiesis in the bone marrow of Cdk5rap2-deficient mice (referee #3, specific comment 2) should not preclude publication there, although following up on the size of primary TER119+ cells (as suggested by referee #1, major point 1) and the tetraploidy of erythroblasts directly isolated from fetal liver (as recommended in point 1 of referee #3) would certainly strengthen the manuscript.

If you should be interested in this possibility, please simply transfer the manuscript (using the link below) at this point and prior to any revision - I would then alert my EMBO Reports counterpart, Dr. Deniz Senyilmaz Tiebe, who would be happy to further discuss all aspects of a revision with you, and send you a formal revision invitation for EMBO Reports.

I am sorry that I could not be more positive for The EMBO Journal at this stage, but would nevertheless like to thank you again for having had the opportunity to consider this work, and very much hope you find the possibility of transfer to EMBO reports worthwhile.

REFEREE REPORTS

****

Referee #1:

Centrosome function is critical during terminal erythroid differentiation.

Centrosomes are microtubule-organizing organelles that are important for forming the bipolar spindle in mitosis to allow for the accurate segregation of chromosomes into each daughter cell. The centrosome consists of a pair of centrioles and the surrounding pericentriolar material (PCM). Recruitment of PCM is increased in G2 phase to increase microtubule nucleation in preparation for spindle formation in mitosis. The PCM comprises several proteins, including Cdk5rap2, which is dispensable for spindle formation in cultured cells. However, the function of Cdk5rap2 has mainly been studied in cell lines, and its role in cycling

and differentiated cell types under physiological conditions in vivo is much less understood.

In the present study, Tischer et al. investigate the mechanism that causes macrocytic anemia in Cdk5rap2 deficient mice. Using ex vivo erythroid differentiation cultures, Tischer et al. uncover that Cdk5rap2 is critical for proper spindle formation during the late stages of erythropoiesis. Erytroblasts seem to have a higher dependency on PCM formation by Cdk5rap2 due to lower levels of PCNT, yTubulin, and CEP192. The loss of Cdk5rap2 results in impaired late stage mitotic divisions that lead to tetraploidization, providing a compelling explanation for the macrocytic anemia phenotype observed in Cdk5rap2 deficient mice. A similar set of phenotypes were observed when centrioles are depleted in differentiating erythroblasts. Importantly, the effects observed from loss of Cdk5rap2 or centrioles are independent of the previously described mitotic surveillance pathway.

The experiments in this manuscript use a physiologically relevant system to study novel functions of Cdk5rap2 in erythroid differentiation. The study reveals how PCM composition in different cell types can affect mitotic fidelity and confer cell-type-specific vulnerabilities. This nicely highlights the importance of applying and refining knowledge generated from work in immortalized cell lines to the biology of the thousands of cell types present in vivo. I consider this to be an important paper that will be of interest to the broad readership of EMBO Journal. I have listed below some alterations that the authors should consider before publication.

Major points:

• Fig. 1A-F are the only experiments carried out in vivo. I appreciate that studying detailed mechanism is very difficult in vivo and ex vivo cultures are the best system to do so. However, the authors should include an experiment or readout that bridges this gap. Is there any assay that can be used to directly compare the in vivo data in Fig. 1 with the culture system? For example, this could be done by analyzing sorted blood cells by ImageStream to show that directly isolated TER119+ cells also increase cell size, as seen in the ex vivo cultures.

• I appreciate the sophisticated analyses represented by box plots. However, representative images would help the reader to follow and evaluate the quality of the data. This is important for cell cycle profiles in Fig. 3B and H, and for the ImageStream based analysis in Fig. 5F-I. From the ImageStream examples in Fig 1H and 3E, it seems challenging to analyze the different mitotic stages based on the DNA shape. The authors should provide representative images of each mitotic stage.

• The data in Figure 6 provide a plausible and convincing explanation for why Cdk5rap2 is critical for faithful mitosis during late erythropoiesis. This intriguing finding shows that PCM composition differs between cell types. However, the critical experiments in Fig. 6B-F should be repeated to reach at least n=3 biological replicates for each genotype.

• The authors performed the appropriate statistical analyses for their data, but it is unclear whether they based the tests on the number of cells or the biological replicates (number of embryos or litters). This is not stated in the figure legends or the methods section and should be clarified.

• I appreciated that the authors address whether the increase in 4n cells is caused by a delay in G2/M phase. pRB and cyclin A2 levels appear similar across genotypes at 48h (EV Fig. 3H). At that time point, the cells have fully exited the cell cycle (according to EV Fig. G). The authors should include Cdk5rap2 deficient cells in both time points and probe for selected markers as in EV Fig. 3H to provide a more comprehensive result. Alternatively, an appropriate flow cytometry experiment can be performed staining for G2 markers.

Minor points:

• From the experimental data provided, it cannot be excluded that the additional band in the western blots in Fig. 1A and EV Fig. 1 represents a truncated version or isoform of Cdk5rap2. Thus, I don't feel it is correct to refer to the mouse strain as null.

• For non-specialists, it would help to define "anemia". The authors say that there is no clinical anemia but later state that the mouse has a macrocytic anemia phenotype (lines 116-128). How is this defined?

• Including a short introduction on TER119 would help - what it is used for, and which cell types express it?

• The authors write that wt erythroid progenitors divide 5 times whereas Cdk5rap2-null erythroid progenitors enucleate prematurely after 4 divisions (lines 168-184). Based on the presented data this seems to be an overstatement: there is a clear population of wt and Cdk5rap2-null cells that do not divide 5 or 4 times, respectively. The authors should rephrase this.

• The presentation of the data in Fig. 2D-I is not intuitive and hard to follow. It is difficult to reconcile the prolonged cell cycle duration of 8h in the fourth division in Fig. 2E. How can this be explained and why is it not seen in Cdk5rap2-null cells? The authors could consider changing the way the data is presented.

• From the labeling of Fig. 3B it is unclear if the authors quantified S-phase from the BrdU pulse experiment (3A) as in 3H, or based on DNA staining only.

• Fig. 4G shows a quantification of the nuclear area in Centrinone treated EBs. Interestingly, the untreated 4n enucleating EBs show a significant increase in nuclear size over the Centrinone condition. How can this be explained?

• Fig. 5 and 6: The color code in the figure does not match the figure legend (alpha-tubulin is in magenta).

Referee #2:

This paper uses an ex vivo differentiation system to reveal the critical role of the centrosome and its component CDK5RAP2 during erythropoiesis, particularly focusing on terminal erythroid differentiation. Interestingly, this study shows that late CDK5RAP2null erythroblasts or erythroblasts lacking centrosomes display defects in bipolar spindle formation, faithful chromosome segregation as well as cell cycle progression at the 4th division of differentiation. Thus, this mitosis is somehow different because the CDK5RAP2 and centrosome requirement was not observed in previous divisions of erythroid differentiation, embryonic fibroblasts or for example in most cultured cells such as RPE1 (studies by e.g. Chinen et al.). This difference may arise from the decreasing PCM at centrosomes (in particular CEP192) during erythroid differentiation making CDK5RAP2 essential for bipolar spindle formation. As a result of this spindle defect, CDK5RAP2null erythroblasts arrest in mitosis and eventually exit mitosis without chromosome segregation with 4N DNA content after the 4th division. The enlarged 4N nucleus hinders reticulocyte production because of reduced enucleation efficiency, which ultimately leads to macrocytic anaemia like phenotype in which lager and fewer RBC are present.
Despite previous understanding of microtubules in erythroid enucleation, the functional relevance of the MTOCs/centrosomes in terminal erythroid differentiation remained largely unknown. Hence this study provides novel and very important insights to centrosome function and CDK5RAP2 in erythroid differentiation and enucleation. The presented data are of high quality and with very good statistical evaluation. In summary, because the authors provided an understanding of centrosomes and CDK5RAP2 for the essential process of erythropoiesis and the disease connection of this study, I recommend this article for publication in EMBO J.

I only have minor comments that should be addressed by the authors.

1. Based on my knowledge, so far there is no or few human patient-based report of association of anaemia and CDKRAP2 mutations. It could be worth to discuss this aspect in the Discussion.
2. The paper speculates that premature drop in Cyclin B1 level may force cells into G1 without chromosome segregation. This, however, does not fit with data in Figure EV4E and F (no drop of Cyclin B). The rationale to use additional experiment with proteasome inhibitor MG132 to support this conclusion is to some extent weak (MG132 will not only affect Cyclin B1 but a number of targets).
3. Fig. 3F does not show dumbbell-like shaped nuclei as stated in the result part.
4. Some organization of the figures could be optimized. It will be easier to follow if the sub-figures appear in alphabetical orders, for instance in figure 3. It may also be good to label 2N, S and 4N in the lower panel of Figure 3B and Figure 3H.
5. In some cases the figures are mentioned in the text not in alphabetical order. E.g. Figure 6 and EV5 ABCD. The authors should improved this.
6. Figure 6D and 6E can be combined into one graph. It will facilitate the comparison between interphase and mitosis between and within different genotypes.
7. Similarly, I recommend to combine Figure 5F and 5H; and to combine Figure 5G and 5I for easier comparison between DMSO and Nocodazole treated groups.
8. Some terms and markers may need to be explained for readers who are not from the same field. E.g T24, T48, EV1, EV2... and what cells do marker TER119 mark.
9. Representative images maybe included for Figure 6F and 6G.
10. "Upon centrosome loss, cells underwent fewer cell divisions independent of their Trp53 genotype (Figs 5H and EV5B) and in each of these cases the decrease in number of cell division was seen only in enucleating 4N-EBs
392 (Figs 5I and EV5C). " The contents of Fig 5H and Fig 5I do not fit with the text.

Referee #3:

In this manuscript, the authors investigate role of Cdk5rap2, the conserved centrosome component, in regulating murine erythroid differentiation and the mechanistic basis for macrocytic anemia noted in Cdk5rap2-deficient mice. They document that the fetal-derived erythroid progenitors from Cdk5rap2-defcient mice generate fewer and larger reticulocytes that recapitulate

features of macrocytic anemia. In late-stage erythroblasts, but not in embryonic fibroblasts, loss ofCdk5rap2 or pharmacological depletion of centrosomes was shown to result in highly aberrant spindle morphologies and failure to complete mitosis resulting reduced numbers of erythroblasts and generation of tetraploid erythroblasts. Tetraploidy impeded the enucleation process and also resulted in generating larger sized reticulocytes. Based on these findings the authors implicate a critical role for CDK5RAP2 and centrosomes in spindle formation specifically during blood production. They propose that disruption of centrosome and spindle function could contribute to the emergence of macrocytic anemias caused by nutritional deficiency or exposure to chemotherapy. The reported findings further extend our understanding of the role of centrosome and spindle function in regulating erythropoiesis. The comprehensive reported findings are potentially very interesting and the imaging data is very informative.

There are, however, a number of concerns with the interpretation of the reported findings that raise concerns regarding their direct relevance to our understanding of emergence of macrocytic anemia's caused by nutritional deficiency or exposure to chemotherapy.

Specific Comments:

1. While the rationale of the use of the fetal-derived erythroid progenitors from Cdk5rap2-defcient mice for the reported findings is understandable, there is a concern since fetal derived erythropoiesis is different that of bone marrow erythropoiesis, especially in terms of cell size of resultant erythrocytes (Blood Advances 5: 16-25, 2021). Importantly, it will be critical to document that tetraploidy is a feature of erythroblast directly isolated from fetal liver since in vitro cultures, while useful are not optimal for faithful recapitulation of in vivo erythropoiesis. This is of particular concern for study of enucleation which is inefficient in the in vitro culture system especially fetal derived erythroid cells as reflected by that fact only ~15% of erythroblasts enucleated in the culture system.

2. While the findings from the in culture system are informative, it is essential to study in vivo erythropoiesis in bone marrow of the Cdk5rap2-defcient mice which are viable to recapitulate the observed erythroid phenotype. This is necessary since the noted macrocytosis is feature of adult mice. Without such data it will be difficult to critically validate the direct relevance of the reported findings to the documented macrocytosis in the Cdk5rap2-defcient mice.

3. The data on number of cell divisions documented in Figure 2B are intriguing indeed. However, it is a bit concerning that while the noted differences at 48 hours are statistically significant they could be arising from synchronous cell development at late stages of culture. This issue needs to be critically addressed.

4. There is reasonable evidence that orthochromatic erythroblasts that enucleated to generate reticulocytes and pyronocyte have a single centriole that segregated with reticulocyte. It will be helpful to comment on this issue based on the reported findings.

In summery, the reported findings while potentially interesting do not shed novel insights into improved mechanistic understanding of macrocytic anemia in humans

We carefully read your letter along with the reviewers' points and were very pleased about the largely positive and constructive comments we received from Reviewers 1 and 2. We were however deeply disappointed about Reviewer 3's critique, which at places was bordering on unfair. In addition, this Reviewer's comments were rather vague, making it difficult to understand whether his/her problem is with our study or the fetal liver system or the relevance of mouse models to human blood disorders. Nonetheless, below we addressed these issues and included new data (e.g. bone marrow) in support of our conclusions.

This study is multidisciplinary in nature and I realise that my group is not a specialist in erythropoiesis. However, we had valuable advice from colleagues at the Stem Cell Institute in Cambridge when we started this work. We also presented our results at the recent Gordon Research Conference on Red Cells (2019), where my postdoc's talk received great feedback from experts. Not one suggested that her results were irrelevant to anemia or that the fetal liver system was not suitable for our scientific questions. Quite the opposite; they were enthusiastic about the new insights our study provided. Following the receipt of the reviews, I seeked advice from a couple of colleagues who are red blood cell specialist in the Oxford MRC Haematology Unit, and they were also perplexed by the Reviewer's critique.

You very clearly set out the rationale for your decision and highlighted the comments you based this on. While we respect your opinion, we feel that we cannot leave the criticism by Reviewer 3 unchallenged given the amount of work we invested in this project and the quality of data we have generated. We have therefore decided to provide a point-by-point response to Reviewer 3's comments below.

Referee #3:

In this manuscript, the authors investigate role of Cdk5rap2, the conserved centrosome component, in regulating murine erythroid differentiation and the mechanistic basis for macrocytic anemia noted in Cdk5rap2-deficient mice. They document that the fetal-derived erythroid progenitors from Cdk5rap2-defcient mice generate fewer and larger reticulocytes that recapitulate features of macrocytic anemia. In late-stage erythroblasts, but not in embryonic fibroblasts, loss ofCdk5rap2 or pharmacological depletion of centrosomes was shown to result in highly aberrant spindle morphologies and failure to complete mitosis resulting reduced numbers of erythroblasts and generation of tetraploid erythroblasts. Tetraploidy impeded the enucleation process and also resulted in generating larger sized reticulocytes. Based on these findings the authors implicate a critical role for CDK5RAP2 and centrosomes in spindle formation specifically during blood production. They propose that disruption of centrosome and spindle function could contribute to the emergence of macrocytic anemias caused by nutritional deficiency or exposure to chemotherapy.

The reported findings further extend our understanding of the role of centrosome and spindle function in regulating erythropoiesis. The comprehensive reported findings are potentially very interesting and the imaging data is very informative.

The summary provided by the Reviewer is a very accurate reflection of our study, as it is nearly identical to our abstract. We are glad that he/she finds our results *potentially very interesting and the imaging data very informative*. However, rather than *extending our understanding* our study is the first to show a function for centrosomes in terminal erythroid differentiation.

There are, however, a number of concerns with the interpretation of the reported findings that raise concerns regarding their direct relevance to our understanding of emergence of macrocytic anemia's caused by nutritional deficiency or exposure to chemotherapy.

The Reviewer took a strong dislike to the final sentence of our abstract in which we proposed that impaired spindle function **could** contribute to other forms of macrocytic anemias. Because aberrant DNA replication (as in folate deficiency) or DNA repair (as in Fanconi anemia) can cause chromosome abnormalities, which can impair spindle formation and chromosome capture, we did not expect that highlighting this possibility (we used "**could**") would meet with such vehement criticism; we can remove this sentence if preferred. Nonetheless, we stand by the idea that aberrant DNA replication and increased DNA damage **could** lead to mitotic errors and tetraploidisation, thus contributing to macrocytosis.

Specific Comments:

1. While the rationale of the use of the fetal-derived erythroid progenitors from Cdk5rap2-defcient mice for the reported findings is understandable, there is a concern since fetal derived erythropoiesis is different that of bone marrow erythropoiesis, especially in terms of cell size of resultant erythrocytes (Blood Advances 5: 16-25, 2021).

We checked this citation above; the publication further supports that macrocytosis in Cdk5rap2 mutants is real, as not only the size (MCV) but also MCH content of red blood cells (RBCs) is elevated (Manuscript Figure 1F). According to this recent publication, it is vital to use age-matched controls to exclude age-dependent size variations in RBCs. This is exactly what we did throughout our study; each biological replicate of the *ex vivo* culture system is derived from littermate embryos, and all blood (and bone marrow) analyses were performed from age-matched animals.

*Importantly, it will be critical to document that tetraploidy is a feature of erythroblast directly isolated from fetal liver since in vitro cultures **(i),** while useful are not optimal for faithful recapitulation of in vivo erythropoiesis **(ii).** This is of particular concern for study of enucleation which is inefficient in the in vitro culture system especially fetal derived erythroid cells as reflected by that fact only ~15% of erythroblasts enucleated in the culture system **(iii).***

Below, we reply to each of the three points made by the Reviewer (i-iii).

i. According to suggestions by Reviewers 1 and 3, in our revision we will establish cell size and ploidy status of TER119-positive cells in the fetal liver. This is not a trivial task as late orthoEBs (TER119high, CD71low), which corresponds to the stage when tetraploidy becomes apparent in the *ex vivo* culture, represent only a very minor population of TER119-positive cells in the E13.5 fetal liver (**Response**

Figure 1A and 1D). As embryonic development progresses, orthoEB prevalence increases somewhat, and thus older embryos will be used for fetal liver collection.

ii. We strongly disagree with the Reviewer's point that fetal liver cultures are not optimal to study *in vivo* erythropoiesis. We selected this system as it is the most widely used model for mechanistic studies on terminal erythroid differentiation and enucleation.

The ex vivo culture of fetal liver has been successfully used to elucidate mechanisms responsible for adult blood phenotypes. A great example for a study on cell cycle and anemia is the following paper by the Lodish Lab:

- https://www.ncbi.nlm.nih.gov/pmc/articles/PMC3444733/

The anemic Cyclin D3 mouse model described here happens to be judged as an excellent model for macrocytosis in the Blood Advances paper cited by the reviewer.

Examples for additional mechanistic studies using the *ex vivo* fetal liver system:

- Role of dynein in terminal erythropoiesis: https://pubmed.ncbi.nlm.nih.gov/25241935/
- Enucleation: https://pubmed.ncbi.nlm.nih.gov/31945154/
- Trafficking and autophagic degradation: https://elifesciences.org/articles/58504
- Influence of trace metals on erythropoiesis: https://pubmed.ncbi.nlm.nih.gov/30122630/

iii. The reviewer is correct to say that at 48 hours ~15% TER119 positive cells are DNA negative, however, we detect another ~15% of enucleating cells the majority of which displaying extruded nuclei. Thus, at least 30% of TER119 positive cells enucleate during the time course, albeit *in vitro* reticulocytes (RetiC) may not detach as effectively from pyrenocytes as *in vivo*. Furthermore, at 36 hours, RetiC represent 7% of the culture, and this figure increases to 12% and 15% by 42 and 48 hours, respectively. It is unclear if RetiC accumulate over time as little is known of whether they survive in these culture conditions. Thus, RetiC formation may be a more frequent event than our data shows.

2. *While the findings from the in culture system are informative, it is essential to study in vivo erythropoiesis in bone marrow of the Cdk5rap2-defcient mice which are viable to recapitulate the observed erythroid phenotype. This is necessary since the noted macrocytosis is feature of adult mice. Without such data it will be difficult to critically validate the direct relevance of the reported findings to the documented macrocytosis in the Cdk5rap2-defcient mice.*

As suggested by the Reviewer, we started this project by looking at hematopoietic stem and progenitor populations in the bone marrow (see data in **Response Figure 1**). Despite the clear macrocytic anemia phenotype in the peripheral blood (in two independent transgenic models of Cdk5rap2), in the bone marrow we found no obvious defects in hematopoietic stem and progenitor populations in the absence of CDK5RAP2 (**Response Figure 1B).** Furthermore, FACS analysis revealed no changes in percentages of different erythroid populations in the *Cdk5rap2^{null}* bone marrow or fetal liver indicating that terminal erythroid differentiation was not blocked at a particular stage (**Response Figure 1C and 1D**). We also asked if the macrocytosis phenotype was present in the *Cdk5rap2^{null}* bone marrow, and to this end, performed Imagestream analysis. In the absence of CDK5RAP2, TER119-positive cells from the bone marrow exhibited a small increase in size, suggesting that macrocytosis appears during terminal erythroid differentiation (**Response Figure 1E**) as observed in the *ex vivo* culture as well (**Response Figure 1F**).
Our findings that progenitor populations were unaffected but size of TER119-positive cells was increased indicated possible defects during differentiation into late-stage EBs and enucleation. The bone marrow contains a highly heterogenous cell population and late-stage erythroblasts and reticulocytes seemed poorly represented. This prompted us to seek out an alternative model that would enable us to study the kinetics and dynamics of terminal erythroid differentiation in a more homogeneous population, and this is why we selected the fetal liver culture.

Briefly, erythroid progenitors (TER119 negative) are isolated from the fetal liver at E13.5 and induced to differentiate with Erythropoietin treatment. Cells complete ~5 cell divisions and enucleate by 48 hours in culture. During these 48 hours cell behaviour can be tracked through terminal erythroid differentiation in the presence of genetic/chemical perturbations. There are two additional advantages of the fetal liver

system; much greater number of biological repeats can be performed especially when the strain is homozygous sterile like Cdk5rap2. 5 homozygous fetal livers require 4 adult mice, whereas 5 homozygous adults require generation of up to 40 adult mice. This leads to more robust data from fewer animals. Moreover, Cdk5rap2^{null} adults exhibit mild proportional dwarfism, which could impact on bone marrow cellularity and have other physiological effects as well.
We did not include the bone marrow data in our submission as we thought that macrocytic anemia in Cdk5rap2 knockout mouse had already been well documented in the Hertwig's anemia model, and the EUCOMM model we are using clearly shows the same phenotype. However, we would be of course happy to include these results in the hope that it satisfies the Reviewer.

3. The data on number of cell divisions documented in Figure 2B are intriguing indeed. However, it is a bit concerning that while the noted differences at 48 hours are statistically significant they could be arising from synchronous cell development at late stages of culture. This issue needs to be critically addressed.

We do not know what the Reviewer means by synchronous cell development; it is not a term we have come across with. If the reviewer wants to know if late-stage wild type erythroblasts all divide at the same time (synchronously) compared to those lacking Cdk5rap2, then we can exclude this possibility based on the live cell data, which did not reveal a synchronous final division across the culture, albeit final division and enucleation occurred at similar times in cells that were siblings. Also, we did not notice any blockade at particular stages of terminal erythroid differentiation in the Cdk5rap2 mutants (**Response Figure 1D**) and so differentiation seems to occur with similar kinetics in mutants and wild-type.

4. There is reasonable evidence that orthochromatic erythroblasts that enucleated to generate reticulocytes and pyronocyte have a single centriole that segregated with reticulocyte. It will be helpful to comment on this issue based on the reported findings.

Previous publications reported the number of centrioles in enucleating EBs/RetiC based on selected EM images; they concluded that enucleating EBs contain 1-2 centrioles (Skutelsky, E and Danon D, 1970). Imagestream analysis revealed that in orthoEBs microtubules radiate from a γ-tubulin rich area, which is in line with the idea that functional centrosomes are sustained in late-stage EBs (Konstantinidis, et al., 2012). These papers reported observations without any quantitative data. Our quantification of centrosome numbers in Manuscript Figure 4C (or **Response Figure 1G**) shows that 80% of enucl. EBs and RetiC contain two γ-tubulin-positive dots, consistent with presence of a pair of disengaged centrioles. However, there is a small but significant increase in enucl. EBs and RetiC in cells with a single γtubulin, total prevalence only reaching ~20%. It is feasible that in these cells the two centrioles are too close to be resolved (or on top of each other) but we cannot exclude that some cells genuinely have a single centriole or two centrioles with only one incorporating PCM. Our comprehensive analysis however argues for presence of two disengaged centrioles in most enucl. EBs and RetiC.

In summery, the reported findings while potentially interesting do not shed novel insights into improved mechanistic understanding of macrocytic anemia in humans.

This is a rather harsh comment that shows a complete misunderstanding of the purpose of our study; the Reviewer criticises the final sentence of our abstract again. We set out to understand the role of centrosomes in erythroid differentiation and identified a cell type- and differentiation-specific function for the organelle in spindle formation. In addition, we find that Cdk5rap2 loss or centrosome depletion leads to tetraploidisation of late-stage erythroblasts resulting in larger reticulocytes that are a feature of macrocytosis. We do believe that our data provide significant and new mechanistic insights into terminal erythroid differentiation. In the study we used a mouse model and did not claim to directly work on macrocytic anemias in humans.

Response Figure 1

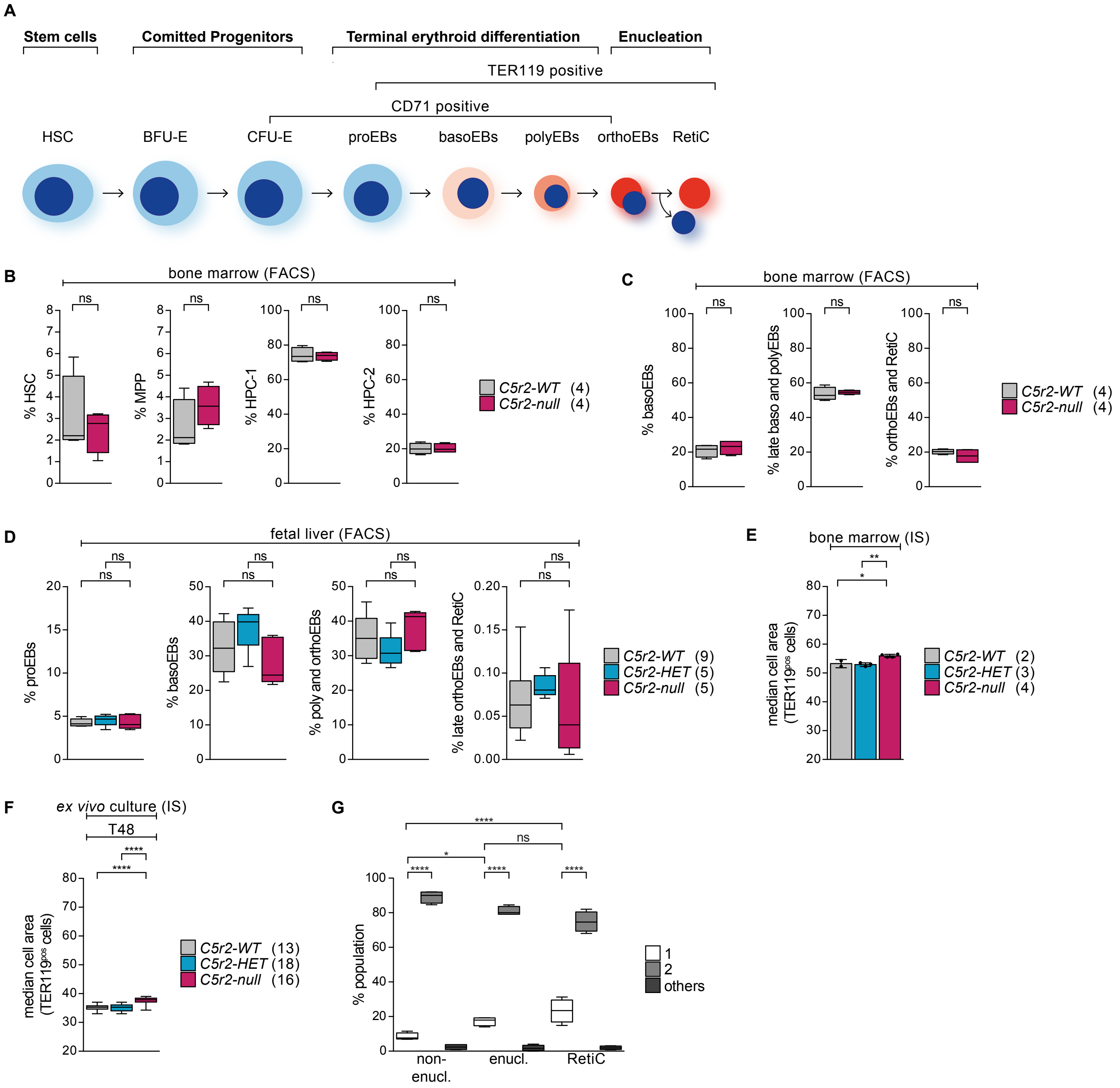

A Schematic showing stages of erythropoiesis and expression of surface markers CD71 and TER119.
B and C Quantification of hematopoietic stem and progenitor cells (A) and erythroblast stages (B) in bone marrow of 10-13 week old mice. Genotypes are as indicated. HSC = hematopoietic stem cells, MPP = multipotent hematopoietic progenitors, HPC = hematopoietic progenitor cells.
D Quantification of erythroblast stages in E13.5 fetal livers. Genotypes are as indicated.
E Quantification of size of TER119^{pos} cells in the bone marrow of 10-week old mice using Imagestream (IS). Genotypes are as indicated.
F Quantification of cell size in *ex vivo* cultured TER119^{pos} cells after 48 hours. Genotypes are as indicated.
G Centrosome number in *ex vivo* cultured non-enucl.EBs, enucl. EBs and RetiC. Four independent experiments with a total number of 2618 (non-enucl.), 715 (enucl.) and 877 (RetiC) were analysed. Statistical difference was determined by Two-way ANOVA with Tukey's multiple comparisons test.

Data information: The number in brackets correspond to the number of mice (B - D), embryos (E and F) or litters (G) analyzed. All box plots show 5th,95th (whiskers) and 25th, 50th and 75th percentile (boxes). Bar graphs display mean ± standard deviation. Statistical significance was determined by Mann-Whitney test (B and C), One-way ANOVA with Tukey's multiple comparisons test (D – F) or Two-way ANOVA with Tukey's multiple comparisons test (G).

Thank you for your patience during our consideration of the points you raised in response to the referee reports on your recent EMBO Journal submission on CDK5RAP2 and erythroid differentiation. I contacted an advisor with expertise in erythropoiesis to arbitrate on your answers to referee 3, and I am pleased to inform you that this arbitrator generally agreed with your arguments and found the study in principle suitable for publication. This is reflected in their comments copied below, as well as in the attached commented version of your point-by-point response - I hope you will be able to see these comments in the edited PDF.

As you will see, the arbitrator recommends a few additional analyses that would be helpful, in addition to the experiments you have already offered to include in response to referee 3 - in particular adding reticulocyte counts in peripheral blood, and looking for signs of stress erythropoiesis in the spleen. With these additions, we would be happy to further consider a manuscript revised along the lines proposed in your response letter for eventual publication in The EMBO Journal. I am therefore inviting you to resubmit your study once you have incorporated these revisions, together with a final point-by-point response letter answering the three original reports as well as the arbitrator's comments; we would then turn the study into a formal revision in our submission system.

When preparing your revision, please also take note of the editorial points listed below the arbitrator comments - the editorial process at the stage of resubmission should be greatly facilitated by their complete incorporation at this point, and by close adherence to our Author Guidelines for revised manuscripts.

Should you have any further questions regarding this revision, please do not hesitate to contact me. I look forward to receiving your revised study!

ARBITRATOR COMMENTS:

General comments:
On the whole, I agree with the authors. Their study is solid and use of fetal liver cell cultures is fully justified. Of course cultured cells are different from the in vivo environment, but Ref 3 forgets that ex vivo experiments often help to reveal cellular phenotypes that are not tractable in vivo. For instance, apoptotic cells are rapidly cleared in vivo and the number of differentiation divisions can only be assessed quantitatively in the ex vivo. I believe the authors have found the right balance between in vivo and ex vivo experiments.

Below, a few more remarks. Most of these things could be easily done and would help to define the erythrocyte macrocytosis (or anemia) of the animals better.

Specific comments:

Abstract:
"We propose that disruption of centrosome and spindle function could contribute to the emergence of macrocytic anemias caused by nutritional deficiency or exposure to chemotherapy."
I propose to rephrase this to:
"We propose that disruption of centrosome and spindle function could contribute to the emergence of erythrocyte macrocytosis, for instance due to nutritional deficiency or exposure to chemotherapy."

Results.
From the data presented in the paper I would conclude these mice are not anemic, they display erythrocyte macrocytosis. The animals could however have compensated anemia (see below).

It would be useful to add reticulocyte counts to Figure 1F. Increased reticulocyte counts are an indication of compensated anemia. In addition, measuring EPO levels in serum would be useful (there are ELISA kits for this).

Did the authors assess the spleen/body weight ratios of the animals? In mice, stress erythropoiesis takes place in the spleen. When activated, the spleen increases in size.

It would be very interesting to determine how the animals recover from induced anemia. Phenylhydrazine-induced anemia is often used to assess this. I expected this had been reported previously on the Hertwig's anemia mouse model, but I can't find anything in the literature. I accept that it is probably a regulatory bridge to far to add this to the paper.

Discussion.
"Despite elevated TP53 levels in Cdk5rap2null and centrinone-B-treated EBs, the phenotypes persisted in absence of Trp53, suggesting that the macrocytic anemia in adult mice might also be independent of TP53."
This should have been measured, this could be easily done if some of the compound knockout animals (Figure 7F) would have been allowed to come to term. A missed opportunity!

Response to Referees

We would like to thank all three Reviewers and the Arbitrator for their careful assessment of our manuscript. We appreciate their useful suggestions as well as their time and effort. Our replies below are in black whereas referees' comments are in blue. **Response Figure 1** is attached at the end of this document.

Referee #1:

Centrosomes are microtubule-organizing organelles that are important for forming the bipolar spindle in mitosis to allow for the accurate segregation of chromosomes into each daughter cell. The centrosome consists of a pair of centrioles and the surrounding pericentriolar material (PCM). Recruitment of PCM is increased in G2 phase to increase microtubule nucleation in preparation for spindle formation in mitosis. The PCM comprises several proteins, including Cdk5rap2, which is dispensable for spindle formation in cultured cells. However, the function of Cdk5rap2 has mainly been studied in cell lines, and its role in cycling and differentiated cell types under physiological conditions in vivo is much less understood.

In the present study, Tischer et al. investigate the mechanism that causes macrocytic anemia in Cdk5rap2 deficient mice. Using ex vivo erythroid differentiation cultures, Tischer et al. uncover that Cdk5rap2 is critical for proper spindle formation during the late stages of erythropoiesis. Erytroblasts seem to have a higher dependency on PCM formation by Cdk5rap2 due to lower levels of PCNT, yTubulin, and CEP192. The loss of Cdk5rap2 results in impaired late stage mitotic divisions that lead to tetraploidization, providing a compelling explanation for the macrocytic anemia phenotype observed in Cdk5rap2 deficient mice. A similar set of phenotypes were observed when centrioles are depleted in differentiating erythroblasts. Importantly, the effects observed from loss of Cdk5rap2 or centrioles are independent of the previously described mitotic surveillance pathway.

The experiments in this manuscript use a physiologically relevant system to study novel functions of Cdk5rap2 in erythroid differentiation. The study reveals how PCM composition in different cell types can affect mitotic fidelity and confer cell-type-specific vulnerabilities. This nicely highlights the importance of applying and refining knowledge generated from work in immortalized cell lines to the biology of the thousands of cell types present in vivo. I consider this to be an important paper that will be of interest to the broad readership of EMBO Journal. I have listed below some alterations that the authors should consider before publication.

We thank the Reviewer for his/her positive comments and highlighting that our paper is important and of interest to the broad readership of EMBO Journal. We included our point-by-point response below.

Major points:

• Fig. 1A-F are the only experiments carried out in vivo. I appreciate that studying detailed mechanism is very difficult in vivo and ex vivo cultures are the best system to do so. However, the authors should include an experiment or readout that bridges this gap. Is there any assay that can be used to directly compare the in vivo data in Fig. 1 with the culture system? For example, this could be done by analyzing sorted blood cells by ImageStream to show that directly isolated TER119+ cells also increase cell size, as seen in the ex vivo cultures.

We appreciate these suggestions, and in response, we performed a number of new experiments as detailed below.

1. We performed the experiment in foetal liver as suggested. In *ex vivo* cultures, the cell size difference was most obvious in enucleating EBs (**Fig. 3C**) and so we focused our efforts on the same population in freshly harvested FL. Imagestream analysis of FL from littermate control and CDK5RAP2-deficient embryos found ~3% of FL cells to be in the enucleating phase, as opposed to ~15% at 48 hours (T48) of *ex vivo* culture (**Response Fig. 1A**). The low percentage of enucleating cells suggests that the majority of FL cells at E14.5 correspond to an earlier differentiation stage than the (much more homogenous) *ex vivo* population at T48. This is further supported by the average size of TER119-positive cells in FL being comparable to that of *ex vivo* cultures at T24 (**Response Fig. 1B**

shows *ex vivo* vs FL data). In line with *ex vivo* culture at T24, we could not detect a change in size of CDK5RAP2null EBs in FL. Although there was a minor upshift in the size of enucleating cells in CDK5RAP2-deficient FL, this did not reach statistical significance (**Response Fig. 1C**). We noted that enucleating FL cells were ~20% larger than those in *ex vivo* cultures and displayed a greater variability in size (**Response Fig 1C**; see heterozygotes on right graph), indicating that we would have needed more homozygous embryos to reach a firm conclusion. Timed mating of 10 heterozygous females yielded only 4 litters, and in total 4 homozygous embryos, a much lower success rate than usual. Due to closure of our mouse colony on 1 Oct, we could not perform further timed matings.
In parallel experiments, we decided to investigate spindle morphology in these freshly harvested FL. *Cdk5rap2*^{null} FL cells exhibited a high prevalence of abnormal spindles of similar appearance to those observed in *ex vivo* culture (**Fig. EV4C**). This result strongly supports the notion that the mitotic defects observed in *ex vivo* also occur in whole FL without any culturing. We also expanded our discussion to include these results (from line 480).

2. In addition to foetal liver, we now include in-depth analysis of bone marrow of adult mice (**Fig.EV1G-I**). Our data reveals no obvious changes in hematopoietic stem cell and progenitor populations, and erythroid differentiation also seems unperturbed. We however observed an overall increase in size of TER119 positive cells in line with the macrocytosis phenotype (**Fig. EV1J**). As in foetal liver, percentage of enucleating cells was too low in the bone marrow (~3%) for meaningful downstream analysis.

• I appreciate the sophisticated analyses represented by box plots. However, representative images would help the reader to follow and evaluate the quality of the data. This is important for cell cycle profiles in Fig. 3B and H **(i)**, and for the ImageStream based analysis in Fig. 5F-I **(ii)**. From the ImageStream examples in Fig 1H and 3E, it seems challenging to analyze the different mitotic stages based on the DNA shape. The authors should provide representative images of each mitotic stage.

i. In **Fig 3A and G**, we have included exemplary cell cycle profiles of Cdk5rap2 wildtype and null TER119^{pos} cells from Imagestream analysis of BrdU pulse experiments. The same gating strategy was applied for all samples and the enucleating EB population.

ii. We have included representative ImageStream images of *ex vivo* cultured EBs at different mitotic stages in Fig 5E.

• The data in Figure 6 provide a plausible and convincing explanation for why Cdk5rap2 is critical for faithful mitosis during late erythropoiesis. This intriguing finding shows that PCM composition differs between cell types. However, the critical experiments in Fig. 6B-F should be repeated to reach at least n=3 biological replicates for each genotype.

We performed the following additional biological replicates:

i. **Fig 6C**: Additional repeat for PCM levels at centrosomes in EBs was performed and corresponding graph and section in results and figure legends were updated accordingly.

ii. **Fig 6D-F**: Additional repeat for immunofluorescence experiments was performed and graphs for mean PCNT signal at mitotic centrosomes in **Figure 6D** were updated. Experiments for y-tubulin signal intensities were repeated and quantification of mean signal intensities at interphase and mitotic centrosomes are shown in Figure 6E. The corresponding text in the results section and figure legend were updated accordingly.

iii. **Fig 6A**: In our revised manuscript, we have included new data where we quantify CEP192 and PCNT levels at interphase and mitotic centrosomes of MEFs. These further confirm the observed differences of centrosomes in MEFs and erythroblasts. The corresponding figure legend and text section were updated accordingly.

• The authors performed the appropriate statistical analyses for their data, but it is unclear whether they based

the tests on the number of cells or the biological replicates (number of embryos or litters). This is not stated in the figure legends or the methods section and should be clarified.

We now highlight the number of animals/cells used for each datasets not only in the corresponding figure legend but have also updated all our 'Data information' sections (at the end of each figure legend) with these important details.

• I appreciated that the authors address whether the increase in 4n cells is caused by a delay in G2/M phase. pRB and cyclin A2 levels appear similar across genotypes at 48h (EV Fig. 3H). At that time point, the cells have fully exited the cell cycle (according to EV Fig. G). The authors should include Cdk5rap2 deficient cells in both time points and probe for selected markers as in EV Fig. 3H to provide a more comprehensive result. Alternatively, an appropriate flow cytometry experiment can be performed staining for G2 markers.

Unfortunately, due to limited number of females with the correct genotype, and our misfortune with the timed matings as detailed above, we were unable to perform the experiment in CDK5RAP2-deficient cells. However, given the similarities between the phenotypes of CDK5RAP2- and centrosome-deficient EBs, we decided to use centrinone-treated erythroblasts to address this question. We now include immunoblots of phospho-Rb and cyclinA2 levels at both timepoints (T24 and T48) in **Figure EV3L and M**. Phospho-Rb and cyclin A2 levels decreased from T24 to T48 both in presence or absence of centrosomes. These results collectively argue against centrosome-depleted 4N EBs enucleating from G2.

Minor points:

• From the experimental data provided, it cannot be excluded that the additional band in the western blots in Fig. 1A and EV Fig. 1 represents a truncated version or isoform of Cdk5rap2. Thus, I don't feel it is correct to refer to the mouse strain as null.

In our original submission, we performed native PAGE analysis (**Fig. EV1D**) with our antibody to distinguish whether the lower migrating form in Fig 1A and EV1C was a genuine isoform of Cdk5rap2 or a non-specific band. As the antibody recognised a single CDK5RAP2 band in wildtype MEFs, and this band was absent in CDK5RAP2-deficient MEFs, we concluded that the additional band in SDS-PAGE was likely to be a non-specific protein recognised by the antibody under denaturing conditions. Nevertheless, we realise that given the size of the CDK5RAP2 gene (36 exons), and our antibody recognising only products containing exon 7, we cannot exclude that N-terminally truncated protein products are made. However, such truncated proteins could be made only from transcripts lacking exon 7 and exon 7 seems to be a common exon between known splice variants. We highlight these points in the manuscript (lines 106-117). Notwithstanding, if the reviewer still would like us to change terminology to mutant, delta or tm1b, we could accommodate this request in our final submission.

• For non-specialists, it would help to define "anemia". The authors say that there is no clinical anemia but later state that the mouse has a macrocytic anemia phenotype (lines 116-128). How is this defined?

In lines 120-126, we have included a definition of anemia and also highlighted which characteristic changes in the red blood cell count reflect the specific type (i.e. macrocytic, normochromic) of anemia observed in mice lacking Cdk5rap2.

• Including a short introduction on TER119 would help - what it is used for, and which cell types express it?

A short description of TER119 expression pattern was added to the introduction (line 51) and also highlighted in Fig 1H.

• The authors write that wt erythroid progenitors divide 5 times whereas Cdk5rap2-null erythroid progenitors enucleate prematurely after 4 divisions (lines 168-184). Based on the presented data this seems to be an overstatement: there is a clear population of wt and Cdk5rap2-null cells that do not divide 5 or 4 times, respectively. The authors should rephrase this.

We updated this section to provide a clearer explanation for the observed differences in number of divisions before enucleation and rephrased the conclusions accordingly (lines 198-214).

• The presentation of the data in Fig. 2D-I is not intuitive and hard to follow. It is difficult to reconcile the prolonged cell cycle duration of 8h in the fourth division in Fig. 2E. How can this be explained and why is it not seen in Cdk5rap2-null cells? The authors could consider changing the way the data is presented.

In response to these comments, we changed the order of these panels in Fig 2 to match their alphabetical appearance in the text and merged the wild-type and null cells into the same graph for easier visualisation (**Fig. 2D**). Furthermore, prompted by the Reviewer's comments we changed the schematic in **Fig 2C** and simplified presentation of our data of cell cycle durations altering the text accordingly. Previously, average cell cycle duration was calculated per event, which did not take into account whether cells continued to divide or enucleate (old Fig 2E, H). The updated graph in **Fig 2E** shows duration of each cell cycle relative to the enucleation event; cell cycle –1 (CC–1) precedes enucleation, and CC –2 precedes CC –1 and so on. The new graph clearly demonstrates that cell cycles are around 6 hours in both Cdk5rap2 wild-type and null EBs except for the duration of the final cell cycle preceding enucleation, which increases in both genotypes. We hope these changes have helped to clarify the message.

• From the labeling of Fig. 3B it is unclear if the authors quantified S-phase from the BrdU pulse experiment (3A) as in 3H, or based on DNA staining only.

In **Fig 3B** we used BrdU to determine S-phase population. For consistency, we now refer to S phase as "BrdU^{pos} population" whenever cell cycle profile is derived from BrdU pulse experiments (Fig 3, 4, 7, EV3 and EV5).

• Fig. 4G shows a quantification of the nuclear area in Centrinone treated EBs. Interestingly, the untreated 4n enucleating EBs show a significant increase in nuclear size over the Centrinone condition. How can this be explained?

We noticed this difference in nuclear area not only in centrinone-treated enucleating 4N-EBs but also in Cdk5rap2-deficient enucleating 4N-EBs (**Fig. 3D**). This phenotype appears only in the 4N and not the 2N population of centrosome- and CDK5RAP2-deficient enucleating cells and thus we can exclude the possibility of centrosomes having a general impact on chromatin condensation levels. It is tempting to speculate that the reduced nuclear area in these 4N cells reflects chromatin hypercondensation, which could be a consequence of mitotic delay/failed mitosis. The 4N population is much smaller in controls, and such cells may arise via a number of different mechanisms such as endoreduplication or cytokinesis failure. Alternatively, because chromatin condensation is a key feature of erythroid differentiation, slower enucleation kinetics of centrosome- and CDK5RAP2-deficient 4N cells may allow for greater chromatin condensation. We believe investigating this phenotype is beyond the scope of this work but we agree that this is an interesting point and now describe the phenomenon in the Results section (line 298).

• Fig. 5 and 6: The color code in the figure does not match the figure legend (alpha-tubulin is in magenta).

We have updated the colour code in the figure legend for **Fig 5** and **Fig EV4** to match colours of the representative cells shown in the figures.

Referee #2:

This paper uses an ex vivo differentiation system to reveal the critical role of the centrosome and its component CDK5RAP2 during erythropoiesis, particularly focusing on terminal erythroid differentiation. Interestingly, this study shows that late CDK5RAP2null erythroblasts or erythroblasts lacking centrosomes display defects in bipolar spindle formation, faithful chromosome segregation as well as cell cycle progression at the 4th division of differentiation. Thus, this mitosis is somehow different because the CDK5RAP2 and centrosome requirement was not observed in previous divisions of erythroid differentiation, embryonic fibroblasts or for example in most cultured cells such as RPE1 (studies by e.g. Chinen et al.). This difference may arise from the decreasing

PCM at centrosomes (in particular CEP192) during erythroid differentiation making CDK5RAP2 essential for bipolar spindle formation. As a result of this spindle defect, CDK5RAP2null erythroblasts arrest in mitosis and eventually exit mitosis without chromosome segregation with 4N DNA content after the 4th division. The enlarged 4N nucleus hinders reticulocyte production because of reduced enucleation efficiency, which ultimately leads to macrocytic anaemia like phenotype in which lager and fewer RBC are present.
Despite previous understanding of microtubules in erythroid enucleation, the functional relevance of the MTOCs/centrosomes in terminal erythroid differentiation remained largely unknown. Hence this study provides novel and very important insights to centrosome function and CDK5RAP2 in erythroid differentiation and enucleation. The presented data are of high quality and with very good statistical evaluation. In summary, because the authors provided an understanding of centrosomes and CDK5RAP2 for the essential process of erythropoiesis and the disease connection of this study, I recommend this article for publication in EMBO J.

I only have minor comments that should be addressed by the authors.

We would like to thank the Reviewer for the positive evaluation of our manuscript and the useful comments below. We are delighted that he/she finds it novel, high quality and important, stressing its suitability for publication in EMBO Journal.

Referee #2, Minor Points:

1. Based on my knowledge, so far there is no or few human patient-based report of association of anaemia and CDKRAP2 mutations. It could be worth to discuss this aspect in the Discussion.

The reviewer is correct, to the best of our knowledge no such analysis has been performed. We now mention this in the discussion (line 463).

2. The paper speculates that premature drop in Cyclin B1 level may force cells into G1 without chromosome segregation. This, however, does not fit with data in Figure EV4E and F (no drop of Cyclin B). The rationale to use additional experiment with proteasome inhibitor MG132 to support this conclusion is to some extent weak (MG132 will not only affect Cyclin B1 but a number of targets).

Prompted by these comments, we decided to remove the MG132 data. We agree with the Reviewer that MG132 may stabilise several components in addition to cyclin B1 (a possibility we mentioned in our first submission). We moved the cyclin B1 data to Fig EV3N where in line with other cell cycle markers it argues that 4N-EBs lacking centrosomes are not arrested in mitosis at T48.

3. Fig. 3F does not show dumbbell-like shaped nuclei as stated in the result part.

We thank the Reviewer for pointing out this deficiency. Reference to dumbbell-like shaped nuclei was replaced with "early stages" to match description used in **Fig 3F**.

4. Some organization of the figures could be optimized. It will be easier to follow if the sub-figures appear in alphabetical orders, for instance in figure 3 **(i)**. It may also be good to label 2N, S and 4N in the lower panel of Figure 3B and Figure 3H **(ii)**.

i. The sub-figures in **Fig. 3** and **EV3** were adjusted according to the alphabetical numbers of the panels.
ii. The labelling of cell cycle profiles in the lower panels of **Fig 3B, 3H and EV3E** were included.

5. In some cases the figures are mentioned in the text not in alphabetical order. E.g. Figure 6 and EV5 ABCD. The authors should improved this.

We have updated the results section to match the alphabetical order of the sub-panels in Figure 5. For Figure 2, 6, EV3 and EV5, we have updated the order of the sub-panels to match their alphabetical appearance in the text and adjusted figure legends accordingly.

6. Figure 6D and 6E can be combined into one graph. It will facilitate the comparison between interphase and mitosis between and within different genotypes.

Fig 6D and E show the PCNT signal at mitotic centrosomes at two different timepoints of the ex vivo culture (T24 and T36). We did not combine them into the same graph because the two timepoints were collected from different *ex vivo* cultures/embryos. However, prompted by this comment, we now show them side-by-side for easier comparison in the same figure panel.

7. Similarly, I recommend to combine Figure 5F and 5H; and to combine Figure 5G and 5I for easier comparison between DMSO and Nocodazole treated groups.

Again, we followed the Reviewer's suggestion and combined these into single graphs (now **Fig 5F and G**) and adjusted figure legends accordingly.

8. Some terms and markers may need to be explained for readers who are not from the same field. E.g T24, T48, EV1, EV2... and what cells do marker TER119 mark.

We now include relevant definitions in the main text and also in relevant figure legends. We changed the abbreviation for 'event' to 'ev' to avoid confusion with references to expanded view figures. A short description of TER119 expression pattern was added to the introduction and also highlighted in **Fig 1G**.

9. Representative images maybe included for Figure 6F and 6G.

We have included representative images for these in **Fig EV5A and B**, respectively.

10. "Upon centrosome loss, cells underwent fewer cell divisions independent of their Trp53 genotype (Figs 5H and EV5B) and in each of these cases the decrease in number of cell division was seen only in enucleating 4N-EBs 392 (Figs 5I and EV5C). " The contents of Fig 5H and Fig 5I do not fit with the text.

We thank the Reviewer for noticing these mistakes. This section was updated in the text.

Referee #3:

In this manuscript, the authors investigate role of Cdk5rap2, the conserved centrosome component, in regulating murine erythroid differentiation and the mechanistic basis for macrocytic anemia noted in Cdk5rap2-deficient mice. They document that the fetal-derived erythroid progenitors from Cdk5rap2-defcient mice generate fewer and larger reticulocytes that recapitulate features of macrocytic anemia. In late-stage erythroblasts, but not in embryonic fibroblasts, loss ofCdk5rap2 or pharmacological depletion of centrosomes was shown to result in highly aberrant spindle morphologies and failure to complete mitosis resulting reduced numbers of erythroblasts and generation of tetraploid erythroblasts. Tetraploidy impeded the enucleation process and also resulted in generating larger sized reticulocytes. Based on these findings the authors implicate a critical role for CDK5RAP2 and centrosomes in spindle formation specifically during blood production. They propose that disruption of centrosome and spindle function could contribute to the emergence of macrocytic aneamias caused by nutritional deficiency or exposure to chemotherapy. The reported findings further extend our understanding of the role of centrosome and spindle function in regulating erythropoiesis. The comprehensive reported findings are potentially very interesting and the imaging data is very informative.

We appreciate that the Reviewer considers our findings potentially very interesting and informative.

There are, however, a number of concerns with the interpretation of the reported findings that raise concerns regarding their direct relevance to our understanding of emergence of macrocytic anemia's caused by nutritional deficiency or exposure to chemotherapy.

In our abstract we proposed that impaired spindle function **could** contribute to other forms of macrocytic anemias. This statement is based on reports that aberrant DNA replication (as in folate deficiency) or DNA repair (as in Fanconi anemia) can cause chromosome abnormalities, which can impair spindle formation and chromosome capture. We did not expect that highlighting this possibility (we used "**could**") would meet with such strong criticism. Although, we stand by the idea that aberrant DNA replication and increased DNA damage **could** lead to mitotic errors and

tetraploidisation and thus contribute to macrocytosis. Prompted by this comment and the Arbitrator' suggestion, we have changed the final sentence to:

"We propose that disruption of centrosome and spindle function could contribute to the emergence of macrocytic anemias, for instance due to nutritional deficiency or exposure to chemotherapy."

Specific Comments:

1. While the rationale of the use of the fetal-derived erythroid progenitors from Cdk5rap2-defcient mice for the reported findings is understandable, there is a concern since fetal derived erythropoiesis is different that of bone marrow erythropoiesis, especially in terms of cell size of resultant erythrocytes (Blood Advances 5: 16-25, 2021).

Foetal and adult erythropoiesis are both considered definitive erythropoiesis, and that is why we believed the former to be an adequate model system for our study. We also checked the citation above; the publication further supports that macrocytosis in Cdk5rap2 mutants is real, as not only the size (MCV) but also MCH content of red blood cells (RBCs) is elevated (**Fig 1F**). According to this recent publication, it is vital to use age-matched controls to exclude age-dependent size variations in RBCs. This is exactly what we did throughout our study; each biological replicate of the *ex vivo* culture system is derived from littermate embryos, and all blood (and bone marrow) analyses were performed from age-matched animals.

Importantly, it will be critical to document that tetraploidy is a feature of erythroblast directly isolated from fetal liver since in vitro cultures**,** while useful are not optimal for faithful recapitulation of in vivo erythropoiesis**.** This is of particular concern for study of enucleation which is inefficient in the in vitro culture system especially fetal derived erythroid cells as reflected by that fact only ~15% of erythroblasts enucleated in the culture system**.**

In response to the comments about *ex vivo* culture system:

We disagree with the Reviewer's point that foetal liver cultures are not optimal to study *in vivo* erythropoiesis. We selected this system as it is the most widely used model for mechanistic studies on terminal erythroid differentiation and enucleation.
The reviewer is correct to say that at 48 hours only 15% TER119 positive cells are DNA negative, however, we detect another ~10-15% of enucleating cells the majority of which displaying extruded nuclei. Thus, at least 25-30% of TER119 positive cells enucleate during the time course, albeit *in vitro* reticulocytes (RetiC) may not detach as effectively from pyrenocytes as *in vivo*. Furthermore, at 36 hours, RetiC represent 7% of the culture, and this figure increases to 12% and 15% by 42 and 48 hours, respectively. It is unclear if RetiC accumulate over time as little is known of whether they survive in these culture conditions. Thus, RetiC formation may be a more frequent event than our data shows.

Foetal liver (FL) experiments:

We performed experiments on freshly harvested foetal liver (FL) as suggested. In the *ex vivo* culture at T48, enucleating cells represented ~15% of the TER119-positive population and ~40% of these enucleating cells were 4N/tetraploid according to cell cycle analysis with Imagestream (based on DNA content and BrdU pulse labelling). We would have liked to conduct the same analysis on FL but our animal licence did not permit intraperitoneal injections of pregnant mothers, thereby excluding *in vivo* BrdU labelling of embryos. ImageStream analysis of freshly harvested liver from E14.5 embryos revealed only ~3% of cells to be in the enucleating phase (**Response Fig 1A**). This low frequency together with our inability to label with BrdU meant that we could not fit a reliable cell cycle curve to our data. Furthermore, TER119pos FL cells were of comparable size to those at the 24 hr timepoint (T24) of *ex vivo* culture (**Response Fig 1B**) when tetraploidy is not yet prominent (**Fig. EV3E**, right graph). As an alternative approach, we investigated spindle morphology in freshly harvested whole FL. We found a marked increase in abnormal spindles similar to those observed in the *ex vivo* culture,

which strongly argues that the mitotic defects observed in *ex vivo* are also present in FL (**Fig. EV4C).** We have extended our discussion to include these results (line 480).

2. While the findings from the in culture system are informative, it is essential to study in vivo erythropoiesis in bone marrow of the Cdk5rap2-defcient mice which are viable to recapitulate the observed erythroid phenotype. This is necessary since the noted macrocytosis is feature of adult mice. Without such data it will be difficult to critically validate the direct relevance of the reported findings to the documented macrocytosis in the Cdk5rap2-defcient mice.

As suggested by the Reviewer, we started this project by looking at hematopoietic stem and progenitor populations in the bone marrow and now include this data in **Fig EV1G**. Despite the clear macrocytic anemia phenotype in the peripheral blood (in two independent transgenic models of Cdk5rap2), in the bone marrow we found no obvious defects in hematopoietic stem and progenitor populations in the absence of CDK5RAP2**.** Furthermore, FACS analysis revealed no changes in percentages of different erythroid populations in the *Cdk5rap2^{null}* bone marrow or foetal liver indicating that terminal erythroid differentiation was not blocked at a particular stage (**Fig EV1H**). We also asked if the macrocytosis phenotype was present in the *Cdk5rap2^{null}* bone marrow, and to this end, performed Imagestream analysis. In the absence of CDK5RAP2, TER119-positive cells from the bone marrow exhibited a small increase in size, suggesting that macrocytosis appears during terminal erythroid differentiation (**Fig EV1H**) as observed in the *ex vivo* culture**.** Serum EPO levels are raised in Cdk5rap2 null animals, consistent with ongoing compensatory erythrocytosis, which may explain why anemia in adult mice is subclinical (**Fig 1G**).

Our findings that progenitor populations were unaffected but size of TER119-positive cells was increased indicated possible defects during differentiation into late-stage EBs and enucleation. The bone marrow and foetal liver contains a highly heterogenous cell population and late-stage erythroblasts and reticulocytes seemed poorly represented. This prompted us to seek out an alternative model that would enable us to study the kinetics and dynamics of terminal erythroid differentiation in a more homogeneous population, and this is why we selected the foetal liver culture.

Briefly, erythroid progenitors (TER119 negative) are isolated from the foetal liver at E13.5 and induced to differentiate with Erythropoietin treatment. Cells complete ~5 divisions and enucleate by 48 hours in culture. During these 48 hours cell behaviour can be tracked through terminal erythroid differentiation in the presence of genetic/chemical perturbations. There are two additional advantages of the foetal liver system; much greater number of biological repeats can be performed especially when the strain is homozygous sterile like Cdk5rap2. 5 homozygous foetal livers require 3 adult mice, whereas 5 homozygous adults require generation of up to 40 adult mice. This leads to more robust data from fewer animals. Moreover, Cdk5rap2^{null} adults exhibit mild proportional dwarfism, which could impact on bone marrow cellularity and have other physiological effects as well.

3. The data on number of cell divisions documented in Figure 2B are intriguing indeed. However, it is a bit concerning that while the noted differences at 48 hours are statistically significant they could be arising from synchronous cell development at late stages of culture. This issue needs to be critically addressed.

We are not entirely sure what the Reviewer refers to as synchronous cell development; it is not a term we have come across with. If the Reviewer wants to know if late-stage wildtype erythroblasts all divide at the same time (synchronously) compared to those lacking Cdk5rap2, then we can exclude this possibility based on the live cell data, which did not reveal a synchronous final division across the culture, albeit final division and enucleation occurred at similar times in cells that were siblings. Also, we did not notice any blockade at particular stages of terminal erythroid differentiation in CDK5RAP2-deficient foetal liver (**Fig EV1I**) and so differentiation seems to occur with similar kinetics in knockouts and wildtypes.

4. There is reasonable evidence that orthochromatic erythroblasts that enucleated to generate reticulocytes and pyronocyte have a single centriole that segregated with reticulocyte. It will be helpful to comment on this issue based on the reported findings.

Previous publications reported the number of centrioles in enucleating EBs/RetiC based on selected EM images; they concluded that enucleating EBs contain 1-2 centrioles (Skutelsky, E and Danon D, 1970). Imagestream analysis revealed that in orthoEBs microtubules radiate from a γ-tubulin rich area, which is in line with the idea that functional centrosomes are sustained in late-stage EBs (Konstantinidis, et al., 2012). These papers reported observations without any quantitative data. Our quantification of centrosome numbers in the new **Figure EV2D** shows that 80% of enucl. EBs and RetiC contain two γ-tubulin-positive dots, consistent with presence of a pair of disengaged centrioles. However, there is a small but significant increase in enucl. EBs and RetiC with a single γ tubulin focus. It is feasible that in these cells the two centrioles are too close to be resolved (or on top of each other) but we cannot exclude that some cells genuinely have a single centriole or two centrioles with only one incorporating PCM. Our comprehensive analysis however argues for presence of two disengaged centrioles in most enucl. EBs and RetiC.

In summery, the reported findings while potentially interesting do not shed novel insights into improved mechanistic understanding of macrocytic anemia in humans.

The Reviewer here primarily criticises the final sentence of our abstract, which we have now changed. We set out to understand the role of centrosomes in erythroid differentiation and identified a cell type- and differentiation-specific function for the organelle in spindle formation. In addition, we find that CDK5RAP2 loss or centrosome depletion leads to tetraploidisation of late-stage erythroblasts resulting in larger reticulocytes that are a feature of macrocytosis. We do believe that our data provide significant and new mechanistic insights into terminal erythroid differentiation. In the study we used a mouse model and did not claim to directly work on macrocytic anaemias in humans.

ARBITRATOR COMMENTS:

General comments:
On the whole, I agree with the authors. Their study is solid and use of fetal liver cell cultures is fully justified. Of course cultured cells are different from the in vivo environment, but Ref 3 forgets that ex vivo experiments often help to reveal cellular phenotypes that are not tractable in vivo. For instance, apoptotic cells are rapidly cleared in vivo and the number of differentiation divisions can only be assessed quantitatively in the ex vivo. I believe the authors have found the right balance between in vivo and ex vivo experiments.

Below, a few more remarks. Most of these things could be easily done and would help to define the erythrocyte macrocytosis (or anemia) of the animals better.

We would like to thank the Arbitrator for his/her time and careful evaluation of our manuscript and response to Reviewer 3, and also for the valuable comments that helped us improve the manuscript.

Specific comments:

Abstract:
"We propose that disruption of centrosome and spindle function could contribute to the emergence of macrocytic anemias caused by nutritional deficiency or exposure to chemotherapy."
I propose to rephrase this to:
"We propose that disruption of centrosome and spindle function could contribute to the emergence of erythrocyte macrocytosis, for instance due to nutritional deficiency or exposure to chemotherapy."

We have rephrased this sentence to: "We propose that disruption of centrosome and spindle function could contribute to the emergence of macrocytic anemia, for instance due to nutritional deficiency or exposure to chemotherapy."

Results.
From the data presented in the paper I would conclude these mice are not anemic, they display erythrocyte macrocytosis. The animals could however have compensated anemia (see below).

It would be useful to add reticulocyte counts to Figure 1F. Increased reticulocyte counts are an indication of compensated anemia. In addition, measuring EPO levels in serum would be useful (there are ELISA kits for this).
Did the authors assess the spleen/body weight ratios of the animals? In mice, stress erythropoiesis takes place in the spleen. When activated, the spleen increases in size.

We fully understand the Arbitrator's point about calling the blood phenotype in the mutant mouse anemia. The reason why we referred to this strain as anemic was two-fold. First, there appears to be two definitions of anemia; *Anaemia is a condition in which the number of red blood cells* ***or*** *the haemoglobin concentration within them is lower than normal (by WHO).* We called Cdk5rap2 mutants anemic based on their low RBC count. Second, the classical "Hertwig's anemia" strain was found to carry an exon inversion in Cdk5rap2 and thus we thought adopting the same terminology made sense. In an/an animals haemoglobin levels are also only marginally reduced. Larger blood cells in Cdk5rap2-deficient mice contain more haemoglobin, hence making up for the overall reduction in RBC numbers.

Although our study was not designed to study compensatory erythropoiesis and stress erythropoiesis in adult mice, we wanted to perform some of the additional experiments suggested. Since *Cdk5rap2^{null}* homozygous animals are sterile, and our focus was on *ex vivo* system using the foetal liver, over the past years we prioritised production and use of heterozygous over homozygous animals.
Nonetheless, we have managed to obtain 3 homozygous animals (6-12 weeks) between submitting the manuscript in June and closing down our mouse colony (1 Oct 21) due to my move to Oxford. We realise limitations of using such low numbers of animals but we decided to proceed with these to address questions regarding compensatory mechanisms. We were advised that reticulocyte counts would be too variable in these young animals, so instead, we performed measurements of serum EPO levels following cardiac bleed. EPO levels were considerably higher in homozygous mice than in their wild-type and heterozygous counterparts, consistent with ongoing stress/compensatory erythropoiesis. We could not obtain meaningful data on spleen/body weight ratios from these 3 mice because the variation was just too great across this small cohort that were neither age nor sex matched.
We also note that increased EPO levels in our *Cdk5rap2^{null}* mice are in line with histopathology data available on International Mouse Phenotyping Consortia, which shows mild spleen and bone marrow hyperplasia and extramedullary hematopoiesis in the Cdk5rap2*tm1a* mouse strain.
https://www.mousephenotype.org/data/genes/MGI:2384875#histopath

In summary, given the evidence for compensatory mechanisms, we would prefer using the term anemia to describe our mouse phenotype but if the change is deemed essential, we would alter the relevant parts in our final version.

It would be very interesting to determine how the animals recover from induced anemia. Phenylhydrazine-induced anemia is often used to assess this. I expected this had been reported previously on the Hertwig's anemia mouse model, but I can't find anything in the literature. I accept that it is probably a regulatory bridge to far to add this to the paper.

We agree that this would be an interesting experiment, but as correctly guessed by the Reviewer, we were unable to perform the experiments he/she recommends as our animal licence does not cover treatments with Phenylhydrazine.

"Despite elevated TP53 levels in Cdk5rap2null and centrinone-B-treated EBs, the phenotypes persisted in absence of Trp53, suggesting that the macrocytic anemia in adult mice might also be independent of TP53." This should have been measured, this could be easily done if some of the compound knockout animals (Figure 7F) would have been allowed to come to term. A missed opportunity!

We cannot agree more with the Reviewer, and of course this was on our to-do list. Earlier in the project, we managed to obtain a small cohort of mice in the *Cdk5rap2^{tm1a}* genetic background; data collected from these revealed no obvious rescue of the blood phenotypes upon combined loss in CDK5RAP2 and TRP53. However, only 5 double homozygous mice (4 males and one female) were obtained after a whole year of breeding (over 200 mice in total) and so we decided to pause this

program and shift emphasis on to the tm1b/null strain, which we used throughout our paper. Unfortunately, this tm1b-p53 breeding program had to be stopped abruptly due to Covid. The low yield of double homozygotes was due to sterility of the Cdk5rap2 strain, low female fertility of the p53 strain, and the shorter breeding window in the latter due to tumour development. Therefore, we also attempted several rounds of IVFs; although we were successful in obtaining embryos from some of these (**Fig 6 and EV6**), we failed to get viable litters. Prompted by the Reviewer's comments, however, we have decided to include the data from the *Cdk5rap2*^{tm1a} strain (**Fig EV6H**) and describe them in the results section (lines 427-430).

Response Figure 1

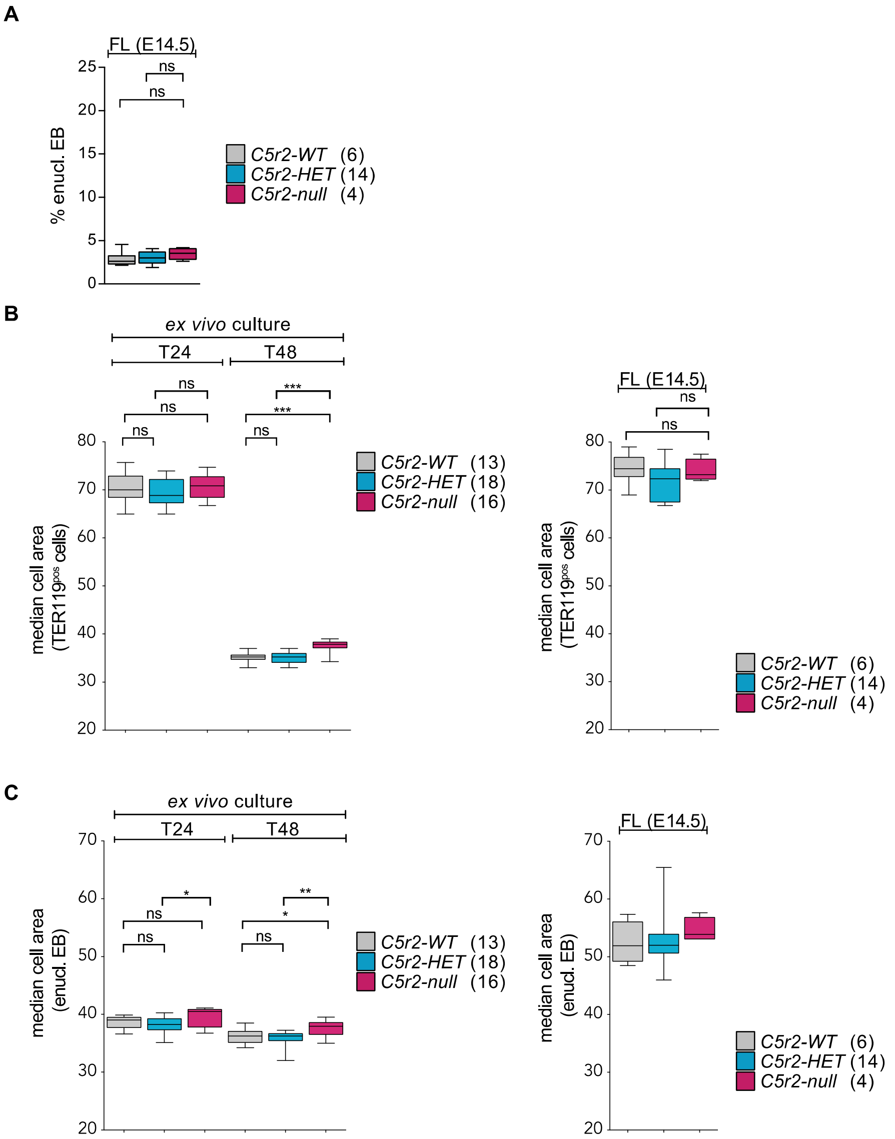

Response Figure 1

A Frequency of enucleating EBs in E14.5 foetal liver (FL).

B Comparison of cell size of TER119-positive cells between ex vivo culture and E14.5 foetal liver (FL).

C Comparison of cell size of enucleating EBs between ex vivo culture and E14.5 foetal liver (FL). Note that at E14.5 TER119pos FL cells are similar in size to cells at T24 of ex vivo culture. Furthermore, enucleating cells are also ~20% larger in FL and display a wider size distribution than in ex vivo culture.

Data information: The number in brackets correspond to the number of embryos analyzed (A - C). All box plots show 5th,95th (whiskers) and 25th, 50th and 75th percentile (boxes). Statistical significance was determined by One-way ANOVA with Tukey's multiple comparisons test (A, B right) or Kruskal Wallis with Dunn's multiple comparisons (B left and C).

Accepted **25th May 2022**

Thank you for submitting your final revised manuscript for our consideration. I have now once more gone through your responses to the original referees, as well as the answers and data provided in response to the arbitrating advisor, and I am pleased to inform you that I have now accepted the study for publication in The EMBO Journal.

EMBO PRESS

YOU MUST COMPLETE ALL CELLS WITH A PINK BACKGROUND ↓

PLEASE NOTE THAT THIS CHECKLIST WILL BE PUBLISHED ALONGSIDE YOUR PAPER

Corresponding Author Name: Fanni Gergely

Journal Submitted to: The EMBO Journal
Manuscript Number: EMBOJ-2021-108739R-Q

USEFUL LINKS FOR COMPLETING THIS FORM

http://www.antibodypedia.com	Antibodypedia
http://1degreebio.org	1DegreeBio
http://www.equator-network.org/reporting-guidelines/improving-bioscience-research-repor	ARRIVE Guidelines
http://grants.nih.gov/grants/olaw/olaw.htm	NIH Guidelines in animal use
http://www.mrc.ac.uk/Ourresearch/Ethicsresearchguidance/Useofanimals/index.htm	MRC Guidelines on animal use
http://ClinicalTrials.gov	Clinical Trial registration
http://www.consort-statement.org	CONSORT Flow Diagram
http://www.consort-statement.org/checklists/view/32-consort/66-title	CONSORT Check List
http://www.equator-network.org/reporting-guidelines/reporting-recommendations-for-tumo	REMARK Reporting Guidelines (marker prognostic studies)
http://datadryad.org	Dryad
http://figshare.com	Figshare
http://www.ncbi.nlm.nih.gov/gap	dbGAP
http://www.ebi.ac.uk/ega	EGA
http://biomodels.net/	Biomodels Database
http://biomodels.net/miriam/	MIRIAM Guidelines
http://jjj.biochem.sun.ac.za	JWS Online
https://osp.od.nih.gov/biosafety-biosecurity-and-emerging-biotechnology/	Biosecurity Documents from NIH
http://www.selectagents.gov/	List of Select Agents

Reporting Checklist For Life Sciences Articles (Rev. June 2017)

This checklist is used to ensure good reporting standards and to improve the reproducibility of published results. These guidelines are consistent with the Principles and Guidelines for Reporting Preclinical Research issued by the NIH in 2014. Please follow the journal's authorship guidelines in preparing your manuscript.

A- Figures

1. Data

The data shown in figures should satisfy the following conditions:

- ➔ the data were obtained and processed according to the field's best practice and are presented to reflect the results of the experiments in an accurate and unbiased manner.
- ➔ figure panels include only data points, measurements or observations that can be compared to each other in a scientifically meaningful way.
- ➔ graphs include clearly labeled error bars for independent experiments and sample sizes. Unless justified, error bars should not be shown for technical replicates.
- ➔ if n< 5, the individual data points from each experiment should be plotted and any statistical test employed should be justified
- ➔ Source Data should be included to report the data underlying graphs. Please follow the guidelines set out in the author ship guidelines on Data Presentation.

2. Captions

Each figure caption should contain the following information, for each panel where they are relevant:

- ➔ a specification of the experimental system investigated (eg cell line, species name).
- ➔ the assay(s) and method(s) used to carry out the reported observations and measurements
- ➔ an explicit mention of the biological and chemical entity(ies) that are being measured.
- ➔ an explicit mention of the biological and chemical entity(ies) that are altered/varied/perturbed in a controlled manner.

- ➔ the exact sample size (n) for each experimental group/condition, given as a number, not a range;
- ➔ a description of the sample collection allowing the reader to understand whether the samples represent technical or biological replicates (including how many animals, litters, cultures, etc.).
- ➔ a statement of how many times the experiment shown was independently replicated in the laboratory.
- ➔ definitions of statistical methods and measures:
  - common tests, such as t-test (please specify whether paired vs. unpaired), simple χ^2 tests, Wilcoxon and Mann-Whitney tests, can be unambiguously identified by name only, but more complex techniques should be described in the methods section;
  - are tests one-sided or two-sided?
  - are there adjustments for multiple comparisons?
  - exact statistical test results, e.g., P values = x but not P values < x;
  - definition of 'center values' as median or average;
  - definition of error bars as s.d. or s.e.m.

Any descriptions too long for the figure legend should be included in the methods section and/or with the source data.

In the pink boxes below, please ensure that the answers to the following questions are reported in the manuscript itself. Every question should be answered. If the question is not relevant to your research, please write NA (non applicable). We encourage you to include a specific subsection in the methods section for statistics, reagents, animal models and human subjects.

B- Statistics and general methods

Please fill out these boxes ↓ (Do not worry if you cannot see all your text once you press return)

1.a. How was the sample size chosen to ensure adequate power to detect a pre-specified effect size?	Sample sizes were not predetermined. For every experiment, we report data from a minimum of 3 embryos for each genotype except for a few exceptions where we were not able to obtain additional embryos. Number of embryos analysed is indicated for each experiment in the relevant Figure legend.
1.b. For animal studies, include a statement about sample size estimate even if no statistical methods were used.	To obtain robust data, we aimed to perform each experiment in fetal livers from at least 5 homozygous embryos, which normally required 3 pregnant females because a single litter usually provided 1-3 homozygous, 1-3 wild-type and 3-6 heterozygous embryos.
2. Describe inclusion/exclusion criteria if samples or animals were excluded from the analysis. Were the criteria pre-established?	Due to sterility of Cdk5rap2 null males and females, only heterozygous animals could be mated, resulting in 0-3 homozygous embryos per litter. Whenever feasible, we selected litters with at least 2 homozygous embryos for our analyses to ensure robust results and minimise differences between sample sizes. Because heterozygous Cdk5rap2 animals displayed no blood phenotypes, we had no preference for number of wild-type embryos per litter as heterozygotes could act as robust controls and crosses produced these in abundance.
3. Were any steps taken to minimize the effects of subjective bias when allocating animals/samples to treatment (e.g. randomization procedure)? If yes, please describe.	n/a
For animal studies, include a statement about randomization even if no randomization was used.	No randomisation was used.
4.a. Were any steps taken to minimize the effects of subjective bias during group allocation or/and when assessing results (e.g. blinding of the investigator)? If yes please describe.	Ex vivo erythroid progenitor cultures derived from embryos of the same litter were processed side-by-side. Cultured erythroid progenitors originating from the same litter were analysed simultaneously via largely automated platforms (FACS and Imagestream) with identical gating strategy.
4.b. For animal studies, include a statement about blinding even if no blinding was done	In this study animals receieved no treatment.
5. For every figure, are statistical tests justified as appropriate?	Yes, description of all relevant statistical tests is included in figure legends. Statistical significance was determined only for comparable datasets acquired under similar conditions.
Do the data meet the assumptions of the tests (e.g., normal distribution)? Describe any methods used to assess it.	Datasets were tested for normality (by d'Agostino or Shapiro-Wilk tests) and variance (Brown/Forsythe or Bartlett's). For data with normal distribution, we used One-way ANOVA with Tukey's multiple comparison. Unequal sample size was common in our datasets (due to genotype variations in litters) but this did not affect outcome because Prism performs Tukey-Kramer test, which allows for unequal sample sizes. For datasets with non normal distribution we employed non-parametric Mann–Whitney U test (pairwise) or Kruskal Wallis test with Dunn's multiple comparisons.

Is there an estimate of variation within each group of data?	Bar charts are presented with standard deviation and box plots provide information on data variability.
Is the variance similar between the groups that are being statistically compared?	Yes, variance was tested as above.

C- Reagents

6. To show that antibodies were profiled for use in the system under study (assay and species), provide a citation, catalog number and/or clone number, supplementary information or reference to an antibody validation profile. e.g., Antibodypedia (see link list at top right), 1DegreeBio (see link list at top right).	The following antibodies were used for flow cytometry staining: B220-FITC (1:300, 561877, BD), CD3e-FITC (1:300, 11-0031-81, eBioscience), CD4-FITC (1:300, 561831, BD), CD8a-FITC (1:300, 561966, BD), Mac1-FITC (1:300, 561688, BD), GR1-FITC (1:300, 11-5931-81, eBioscience), CD11c-FITC (1:300, 11-0114-81, eBioscience), FcεRIα-FITC (1:300, 11-5898-81, eBioscience), TER119-FITC (1:300, 561032, BD), CD41-biotin (1:400, 13-0411-81, eBioscience), CD48-biotin (1:400, 103409, BioLegend), CD150-PE (1:200, 115903, BioLegend), cKit-PerCEP-Cy5.5 (1:200, 105823, BioLegend), Sca1-PE-Cy7 (1:200, 25-5981-81, eBioscience), CD71-PE (1:200, 553267, BD), TER119-APC (1:200, 116212, BioLegend), Streptavidin-BV421 (1:200, 405226, BioLegend) and Streptavidin-AlexaFluor 488 (1:200, 405235, Biolegend).
7. Identify the source of cell lines and report if they were recently authenticated (e.g., by STR profiling) and tested for mycoplasma contamination.	N/A (only freshly harvested mouse cells were used)

* for all hyperlinks, please see the table at the top right of the document

D- Animal Models

8. Report species, strain, gender, age of animals and genetic modification status where applicable. Please detail housing and husbandry conditions and the source of animals.	Mus musculus. Mice used in this study were of the C57BL/6 background. Cdk5rap2tm1a mice (EUCOMM) were kindly provided by David Adams (Wellcome Sanger Institute). Cdk5rap2tm1b mice were generated by crossing Cdk5rap2tm1a mice with PGK-Cre mice. Trp53tm1Tyj/J was used to create Cdk5rap2tm1b;Trp53 double mice.
9. For experiments involving live vertebrates, include a statement of compliance with ethical regulations and identify the committee(s) approving the experiments.	All animal procedures were performed in accordance with the Animal Welfare and Ethical Body of the CRUK Cambridge Institute (CRUK CI, University of Cambridge), and UK Home Office regulations (in accordance with UK law, Animals Scientific Procedures Act 1986).
10. We recommend consulting the ARRIVE guidelines (see link list at top right) (PLoS Biol. 8(6), e1000412, 2010) to ensure that other relevant aspects of animal studies are adequately reported. See author guidelines, under 'Reporting Guidelines'. See also: NIH (see link list at top right) and MRC (see link list at top right) recommendations. Please confirm compliance.	We have done so.

E- Human Subjects

11. Identify the committee(s) approving the study protocol.	n/a
12. Include a statement confirming that informed consent was obtained from all subjects and that the experiments conformed to the principles set out in the WMA Declaration of Helsinki and the Department of Health and Human Services Belmont Report.	n/a
13. For publication of patient photos, include a statement confirming that consent to publish was obtained.	n/a
14. Report any restrictions on the availability (and/or on the use) of human data or samples.	n/a
15. Report the clinical trial registration number (at ClinicalTrials.gov or equivalent), where applicable.	n/a
16. For phase II and III randomized controlled trials, please refer to the CONSORT flow diagram (see link list at top right) and submit the CONSORT checklist (see link list at top right) with your submission. See author guidelines, under 'Reporting Guidelines'. Please confirm you have submitted this list.	n/a
17. For tumor marker prognostic studies, we recommend that you follow the REMARK reporting guidelines (see link list at top right). See author guidelines, under 'Reporting Guidelines'. Please confirm you have followed these guidelines.	n/a

F- Data Accessibility

18: Provide a "Data Availability" section at the end of the Materials & Methods, listing the accession codes for data generated in this study and deposited in a public database (e.g. RNA-Seq data: Gene Expression Omnibus GSE39462, Proteomics data: PRIDE PXD000208 etc.) Please refer to our author guidelines for 'Data Deposition'.<br><br>Data deposition in a public repository is mandatory for:<br>a. Protein, DNA and RNA sequences<br>b. Macromolecular structures<br>c. Crystallographic data for small molecules<br>d. Functional genomics data<br>e. Proteomics and molecular interactions	n/a
19. Deposition is strongly recommended for any datasets that are central and integral to the study; please consider the journal's data policy. If no structured public repository exists for a given data type, we encourage the provision of datasets in the manuscript as a Supplementary Document (see author guidelines under 'Expanded View' or in unstructured repositories such as Dryad (see link list at top right) or Figshare (see link list at top right).	n/a
20. Access to human clinical and genomic datasets should be provided with as few restrictions as possible while respecting ethical obligations to the patients and relevant medical and legal issues. If practically possible and compatible with the individual consent agreement used in the study, such data should be deposited in one of the major public access-controlled repositories such as dbGAP (see link list at top right) or EGA (see link list at top right).	n/a
21. Computational models that are central and integral to a study should be shared without restrictions and provided in a machine-readable form. The relevant accession numbers or links should be provided. When possible, standardized format (SBML, CellML) should be used instead of scripts (e.g. MATLAB). Authors are strongly encouraged to follow the MIRIAM guidelines (see link list at top right) and deposit their model in a public database such as Biomodels (see link list at top right) or JWS Online (see link list at top right). If computer source code is provided with the paper, it should be deposited in a public repository or included in supplementary information.	n/a

G- Dual use research of concern

22. Could your study fall under dual use research restrictions? Please check biosecurity documents (see link list at top right) and list of select agents and toxins (APHIS/CDC) (see link list at top right). According to our biosecurity guidelines, provide a statement only if it could.	n/a
